# Metabolite profiling reveals slow and uncoordinated adjustment of C_4_ photosynthesis to sudden changes in irradiance

**DOI:** 10.1093/plphys/kiaf508

**Published:** 2025-10-13

**Authors:** Stéphanie Arrivault, David Barbosa Medeiros, Cristina Rodrigues Gabriel Sales, Manuela Guenther, Johannes Kromdijk, Alisdair R Fernie, Mark Stitt

**Affiliations:** Max Planck Institute of Molecular Plant Physiology, Am Muehlenberg 1, Potsdam-Golm 14476, Germany; Max Planck Institute of Molecular Plant Physiology, Am Muehlenberg 1, Potsdam-Golm 14476, Germany; Syngenta Seeds Ltda. Rodovia BR 452, Caixa Postal 585, Uberlândia—MG 38407-049, Brazil; Department of Plant Sciences, University of Cambridge, Downing Street, Cambridge CB2 3EA, UK; Max Planck Institute of Molecular Plant Physiology, Am Muehlenberg 1, Potsdam-Golm 14476, Germany; Department of Plant Sciences, University of Cambridge, Downing Street, Cambridge CB2 3EA, UK; Max Planck Institute of Molecular Plant Physiology, Am Muehlenberg 1, Potsdam-Golm 14476, Germany; Max Planck Institute of Molecular Plant Physiology, Am Muehlenberg 1, Potsdam-Golm 14476, Germany

## Abstract

In the field, plants continually experience changes in irradiance. Research in C_3_ species has revealed that while the Calvin–Benson cycle (CBC) adjusts rapidly to changing irradiance, there is substantial loss of photosynthetic efficiency due to slow adjustment of energy dissipation and stomatal conductance. Less is known about the impact of changing irradiance on photosynthetic efficiency in C_4_ species. We subjected maize (*Zea mays*) to a sudden increase or decrease of irradiance in the nonsaturating range and performed time-resolved measurement of photosynthetic rate and profiling of metabolites from the CBC, the CO_2_-concentrating mechanism (CCM), the energy shuttle, photorespiration, and end-product biosynthesis. After a decrease in irradiance, photosynthesis is transiently buffered by energy delivered from transformations in the large metabolite pools in the energy shuttle and CCM. During the subsequent decline in photosynthesis, metabolism transitions to a suboptimal state for photosynthesis in low irradiance, from which it takes several minutes to recover. One reason is that end-product synthesis depletes the metabolite pools that drive intercellular shuttles and time is required to replenish these pools. After an increase in irradiance, there is an initial rapid rise of photosynthesis linked to build-up of CBC intermediates and, probably, activation of enzymes in the CBC and the CCM. This is followed by a further slow rise of photosynthesis linked to gradual accumulation of the large metabolite pools that drive intercellular shuttles. In addition, in both transitions, transient imbalances between pumping and utilization of CO_2_ lead to further losses in photosynthetic efficiency.

## Teaser

Uncoordinated metabolic responses in the CO_2_ concentration cycle and CO_2_ reassimilation by the Calvin–Benson cycle lead to loss of photosynthetic efficiency during light transitions in maize.

## Introduction

Plants in the field experience daily changes of irradiance due to changing sun elevation and shadowing by fixed objects and irregular fluctuations due to clouds and wind movement in canopies (Townsend et al. 2018). The response to changing irradiance is an important factor for photosynthetic efficiency in the field, especially in dense crop canopies (Taylor and Long 2017; Vialet-Chabrand et al. 2017; Townsend et al. 2018; Tanaka et al. 2019).

The response of photosynthetic carbon (C) metabolism to changing irradiance in C_3_ species was investigated in the last century. Wild species that live in the understory of woods and forests where occasional sun flecks provide a large part of total intercepted irradiance show an extreme adaptation. In low light (LL), their Calvin–Benson cycle (CBC) is poised to maintain a very large pool of 3-phosphoglycerate (3PGA), allowing full use of the NADPH and ATP produced during a brief sun fleck without this requiring an increase in flux around the CBC (Pearcy 1990; Pearcy et al. 2005). It typically takes 1 to 2 min to establish steady-state CBC flux after a switch from darkness or LL to higher light (Woodrow and Walker 1980; Laing et al. 1981; Wirtz et al. 1982; Woodrow et al. 1983, 1985; Sage et al. 1987; Mott and Woodrow 2000). During this short lag, CBC intermediates rise and CBC enzymes are posttranslationally activated. In the case of Rubisco, the speed depends on the abundance of Rubisco activase (Woodrow et al. 1996; Mott et al. 1997; Hammond et al. 1998), with a trade-off between abundance of activase, which determines how quickly Rubisco activation responds to changed irradiance, and of Rubisco, which impacts on the rate of photosynthesis under stable irradiance (Woodrow and Mott 1989; Yamori et al. 2012; Carmo-Silva and Salvucci 2013; Kaiser et al. 2016). Activation of plastidic fructose 1,6-bisphosphatase (FBPase) and sedoheptulose 1,7-bisphosphatase (SBPase) by thioredoxin is promoted by rising substrate levels (Woodrow and Walker 1980; Laing et al. 1981; Woodrow et al. 1985; Scheibe 1991; Faske et al. 1995; Stitt et al. 2010; Michelet et al. 2013; Knuesting and Scheibe 2018). In addition, regulation of end-product synthesis poises CBC metabolites at levels that allow a rapid rise in CBC flux when irradiance suddenly increases (Stitt et al. 2010, 2021). After a decrease in irradiance, CO_2_ assimilation decreases abruptly, even when low O_2_ is used to suppress the postillumination photorespiratory burst (Lee et al. 2022; Arce Cubas et al. 2023a). This reflects the extremely rapid turnover of the small pools of NADPH, ATP (about 0.1 s), and CBC intermediates (0.1 to 1 s) (Stitt et al. 1980; Arrivault et al. 2009). The almost instantaneous decline of NADPH and ATP arrests 3PGA reduction, and within 1 to 5 s falling levels of triose-phosphate (triose-P) and other CBC intermediates restrict ribulose-1,5-bisphosphate (RuBP) regeneration and CO_2_ fixation (Prinsley et al. 1986a, 1986b; Stitt et al. 1989). CBC enzymes are inactivated over the next minutes (Woodrow and Walker 1980; Laing et al. 1981; Wirtz et al. 1982; Woodrow et al. 1983; 1985; Sage et al. 1987; Woodrow and Berry 1988; Mott and Woodrow 2000). Overall, in C_3_ plants, the CBC adjusts rather quickly to changes in irradiance. The research focus has shifted to processes that adjust slowly and have a larger impact on photosynthetic performance in changing light like energy dissipation (Kromdijk et al. 2016; Taylor and Long 2017; Tanaka et al. 2019) and stomatal conductance (Lawson et al. 2012; Vialet-Chabrand et al. 2017; Deans et al. 2019).

Less is known about the response of C_4_ photosynthesis to changing irradiance. C_4_ photosynthesis evolved multiple times about 30 million years ago in response to a change in climate, including a drop in atmospheric CO_2_ (Christin et al. 2008; Edwards et al. 2010). This exerted strong selective pressure because Rubisco catalyzes a competing side reaction with O_2_ (Lorimer 1981; Tcherkez et al. 2006), leading to the formation of 2-phosphoglycolate (2PG) that is salvaged by the energetically wasteful process of photorespiration (Osmond 1981; Foyer et al. 2009; Betti et al. 2016; Walker et al. 2016). In C_4_ photosynthesis, oxygenation is partly suppressed by a biochemical CO_2_-concentrating mechanism (CCM) (Osmond and Harris 1971; Hatch 2002; von Caemmerer and Furbank 2003; Sage et al. 2011; Schlüter and Weber 2020). The CCM starts in the mesophyll cells (MCs) where phospho*enol*pyruvate carboxylase (PEPC) incorporates bicarbonate to form 4-carbon (4C) metabolites. These move to the bundle sheath cells (BSCs) and are decarboxylated to generate a high concentration of CO_2_ (Hatch and Osmond 1976; Edwards and Walker 1983; von Caemmerer and Furbank 2003; Weber and von Caemmerer 2010; Schlüter and Weber 2020). This CO_2_ is refixed by Rubisco, which with the rest of the CBC is located in the BSC. The 3-carbon (3C) products of decarboxylation move back to the MC and are used to regenerate phospho*enol*pyruvate (PEP). Intercellular shuttling of 4C and 3C metabolites occurs largely by diffusion (Hatch and Osmond 1976), requiring large pools to generate the necessary intercellular concentration gradients (Leegood 1985; Stitt and Heldt 1985a; Arrivault et al. 2017).

Decarboxylation can occur via NADP-malic enzyme (NADP-ME) in the chloroplast, NAD-malic enzyme (NAD-ME) in the mitochondria, or PEP carboxykinase (PEPK) in the cytosol. Species differ in the major route of C_4_ acid decarboxylation, and this affects which 4C and 3C metabolites move between the 2 cell types, as well as energy requirements in the MC and BSC (Hatch and Osmond 1976; Edwards and Walker 1983; Hatch 1987, 2002; Bräutigam et al. 2018). Decarboxylation by NADP-ME is linked to a malate/pyruvate shuttle, whereas decarboxylation by NAD-ME or PEPCK requires exchange of other metabolites including aspartate, alanine, and possibly PEP. Furthermore, decarboxylation via NADP-ME delivers NADPH to BSC chloroplasts at a rate covering about half the NADPH consumed in the CBC. Many NADP-ME species including maize have dimorphic BSC chloroplasts with strongly decreased photosystem II and NADPH production (Woo et al. 1970; Laetsch 1974; Munekage 2016). The resulting shortfall in NADPH is compensated by a second intercellular “energy” shuttle in which 3PGA moves to the MC and is reduced to triose-P that returns to the BSC. Intercellular movement again occurs by diffusion and requires large pools of 3PGA and triose-P to generate intercellular concentration gradients (Leegood 1985; Stitt and Heldt 1985a; Arrivault et al. 2017). Based on the distribution of capacity for 3PGA reduction (Furbank et al. 1983) and considerations of energy balance, other C_4_ subtypes may operate an analogous shuttle (Hatch 1987; Bräutigam et al. 2014; von Caemmerer and Furbank 2016). However, experimental evidence for the necessary intercellular concentration gradients is missing.

Efficient operation of C_4_ photosynthesis requires coordinated flux in the CCM and the CBC (Furbank et al. 1990; von Caemmerer 2000; Kromdijk et al. 2014). If CO_2_ transfer is too slow, the CO_2_ concentration in the BSC (C_BSC_) will fall and photorespiration will increase. C_4_ plants have substantial activities of enzymes for photorespiration (Osmond and Harris 1971; Onishi and Kanai 1983; Ueno et al. 2005) and carry out photorespiration, although 5- to 10-fold slower than C_3_ plants (Volk and Jackson 1972; de Veau and Burris 1989; Laisk and Edwards 1998; Carmo-Silva et al. 2008; Mallmann et al. 2014; Weissmann et al. 2016; Arrivault et al. 2017; Medeiros et al. 2022). On the other hand, excessive CO_2_ transfer will drive C_BSC_ up, increasing back-leakage to the MC. It is estimated that 13% to 30% of the CO_2_ released in the BSC leaks back to the MC (Farquhar 1983; Hatch et al. 1995; Kromdijk et al. 2014; Bellasio and Griffiths 2014b; Wang et al. 2022) with corresponding wastage of energy.

Even in stable conditions, C_4_ photosynthesis utilizes light less efficiently in low than high irradiance, as revealed by a lower quantum yield (Tazoe et al. 2008; Pengelly et al. 2010; Ubierna et al. 2013; Pignon et al. 2017; Sales et al. 2023). Analysis of ^13^CO_2_ labeling kinetics (Medeiros et al. 2022) revealed several contributing factors including excess flux at PEPC and more back-leakage of CO_2_ (see also Ubierna et al. 2013; Kromdijk et al. 2014), restriction of NADP-ME and a switch toward other decarboxylation routes, less effective use of metabolite pools to drive intercellular shuttles, and higher rates of photorespiration.

In addition to its inherent inefficiency in low irradiance, the topology of C_4_ photosynthesis may render it especially vulnerable to changes in irradiance. Firstly, NADPH and ATP are consumed by numerous reactions in 2 pathways, the CCM and CBC, that are distributed between 2 cell types, each with its own photosynthetic electron transport system. Changing irradiance may lead to local imbalances in redox and energy status that unbalance fluxes in the CCM and CBC. Secondly, the temporal dynamics of metabolite pools differ greatly between the CBC and the CCM. The pools of most CBC intermediates are small with half-times of the order of <0.1 to 1 s (Stitt and Zhu 2014; Arrivault et al. 2017), resembling C_3_ photosynthesis (Stitt et al. 1980; Arrivault et al. 2009). CBC flux per se in C_4_ plants is therefore likely to respond rapidly to a change in irradiance, like in C_3_ plants (see above). In contrast, metabolite pools in the energy shuttle and CCM are large with a relatively slow turnover time (∼10 s, Stitt and Zhu 2014; Arrivault et al. 2017). They are also larger in high irradiance than low irradiance (Usuda 1987; Leegood and von Caemmerer 1988, 1989; Doncaster et al. 1989; Ubierna et al. 2013) presumably to generate larger concentration gradients to drive faster intercellular diffusion. This has interesting theoretical implications for the response to a sudden change in irradiance. On the one hand, after an increase in irradiance, the rise of CO_2_ assimilation might be delayed by slow build-up of energy shuttle and CCM pools. These pools indeed increase slowly during transitions from darkness to light (Furbank and Leegood 1984; Usuda 1985), but there have been no studies in light transitions between different light intensities. On the other hand, these large pools might buffer against a sudden decrease in irradiance. In C_3_ plants, CO_2_ fixation is inhibited almost immediately after a decrease in irradiance, due to the very short half-lives of ATP and NADPH (see above). As pointed out by Stitt and Zhu (2014) (see also Slattery et al. 2018), in C_4_ photosynthesis, conversion of the large pools of triose-P and malate to 3PGA and oxaloacetate could provide enough NADPH and ATP to support rapid CO_2_ fixation for several seconds after a decrease in irradiance. Whereas CO_2_ assimilation decreases abruptly after a drop in irradiance in C_3_ species (see above), the decline is slower in C_4_ species (Laisk and Edwards 1998; Lee et al. 2022; Arce Cubas et al. 2023a). It is, however, unknown if this reflects metabolic buffering and continued CO_2_ assimilation by the CBC, or just continued formation of C_4_ acids by PEPC.

Changes in irradiance lead to posttranslational activation of enzymes. After a dark–light transition, activation of PEPC requires 10 to 15 min (Tirumala Devi and Raghavendra 1992), of pyruvate, phosphate dikinase (PPDK) 2 to 6 min (Roeske and Chollet 1989; Om et al. 2022), and of NADP-MDH up to 10 min (Ashton et al. 1990). Posttranslational activation typically modifies the kinetic properties of enzymes (Doncaster and Leegood 1987; Ashton et al. 1990; Vidal et al. 2002). Enzymes are also posttranslationally regulated by changing light intensity (Chen et al. 2014). However, little is known about the interaction between metabolite levels and posttranslational activation of enzymes under changing irradiance.

Although C_4_ species are classified into subtypes depending on the main route, decarboxylation pathways often operate concomitantly (Hatch 1971; Furbank 2011; Bräutigam et al. 2014; Wang et al. 2014b; Weissmann et al. 2016; Arrivault et al. 2017). This may provide added flexibility (Furbank 2011; Bellasio and Griffiths 2014a; Wang et al. 2014b). Analyses of pool sizes (Usuda 1987; Leegood and von Caemmerer 1989), enzyme activities (Sales et al. 2018), and ^13^CO_2_ labeling kinetics (Medeiros et al. 2022) in steady-state conditions in the NADP-ME species maize indicate that the contribution of other decarboxylation routes increases under low irradiance. It is unknown if this impacts efficiency during light transitions.

There have been few experimental studies of C_4_ photosynthesis during light transitions (see above) and even fewer under fluctuating light. These are differing scenarios, especially when light is fluctuating rapidly (Arce Cubas et al. 2023a), as is typically the case in a canopy. Several studies in fluctuating light regimes reported similar or larger decreases of photosynthetic efficiency in C_4_ than C_3_ species (Kubásek et al. 2013; Li et al. 2021; Lee et al. 2022). As already mentioned, after switching to LL, CO_2_ assimilation declines more slowly in C_4_ than C_3_ plants. This implies that if there is a large decrease in photosynthetic efficiency in C_4_ plants under fluctuating regimes, this is probably due to slow recovery after switching to high light. This was observed for panels of C_3_ and C_4_ species after a sudden increase in irradiance (Kubásek et al. 2013) and under fluctuating light (Li et al. 2021; Lee et al. 2022) but not for 3 pairs of phylogenetically related species under fluctuating light (Arce Cubas et al. 2023a). Apart from this open question, a common theme in many of these studies was that after a change in irradiance, the response of CO_2_ assimilation in C_4_ plants was multiphasic. However, the underlying metabolic factors were not investigated.

Thus, it is an open question what mechanisms impact the efficiency of C_4_ photosynthesis during changes in irradiance. We report experiments in which we subjected maize plants to a sudden decrease or increase in irradiance, monitored the rate of CO_2_ fixation, and harvested material for detailed time-series metabolite profiling. The overall goal was to provide a systems-level overview of the temporal response of C_4_ metabolism to a sudden decrease or increase in irradiance. Specific aims were to (i) assess whether large pools of shuttle metabolites transiently buffer photosynthesis against a sudden drop in irradiance, (ii) assess whether slow build-up of shuttle metabolite pools delays the response to a sudden increase in irradiance, (iii) ask if the contribution of decarboxylation routes changes, and (iv) ask if sudden changes in irradiance perturb the balance between the CCM and CBC. In a parallel study (Sales et al. 2025), we investigated *A_n_* and electron transport in maize exposed to light fluctuating at differing frequencies and profiles metabolites at selected times to learn if detailed analysis of single transitions provides insights into responses in fluctuating regimes.

## Results

### CO_2_ assimilation

**Figure 1.**
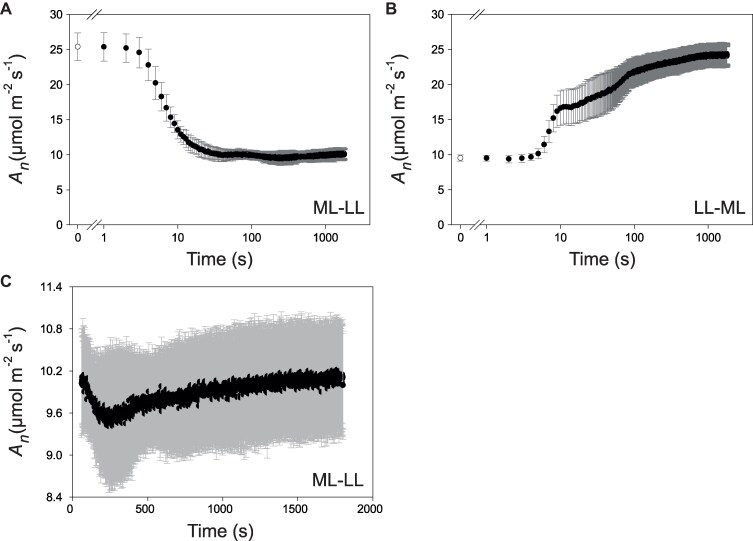
CO_2_ assimilation. **A)** ML–LL transition. Plants were illuminated at growth irradiance (=ML) for at least 4 h and then transferred to 160 *µ*mol photons m^−2^ s^−1^. **B)** Response a LL–ML transition. Plants were illuminated from the start of the light period at 160 *µ*mol photons m^−2^ s^−1^ for at least 4 h before transfer to 550 *µ*mol photons m^−2^ s^−1^. *X* axes correspond to time on a log scale. As gas exchange measurements shortly after the light transitions violate the steady-state assumption underlying default rate equations, dynamic equations were implemented (see Material and methods). Corrected values are shown for each second. Data shown in **A)** and **B)** are mean ± Sd (*n* = 5 for ML–LL, *n* = 6 for LL–ML). The time points in white correspond to time 0, before the light transition. The gap in the *x* axes is for a better visualization of the first 2 time points. **C)** Expansion of the ML–LL response from 60 s onward to show the slight recovery of *A_n_*. The display shows mean ± Sd (*n* = 5). *X* axis corresponds to time on a linear scale. The relatively high Sd is due to differences in *A_n_* between the 5 independent replicates (see Supplementary Data Set 1). Significance was analyzed using a paired *t*-test to compare *A_n_* at different times in a given replicate and separate this from differences in *A_n_* between replicates. The increase between the trough and the end of the transition was significant (*P* = 0.002, *n* = 5, paired *t*-test comparing, for each replicate, the average *A_n_* between 250 and 259 s with average *A_n_* between 1791 and 1800 s). See Supplementary Fig. S1, B and F, for plots of **A)** and **B)** on a linear time scale, Supplementary Fig. S1, C and G, for curve fitting to different time spans of the ML–LL and LL–ML responses, and Supplementary Fig. S1, D, E, H, and I for stomatal conductance and *Ci.* The original data are provided in Supplementary Data Set 1A. The rates given here on an area basis (µmol CO_2_ m^−2^ s^−1^) can be converted to rate on an FW basis (nmol CO_2_ g^−1^ FW s^−1^) using a specific leaf area of 0.0055 m^−2^ g^−1^ (Medeiros et al. 2022).

We investigated the response to a relatively small change of irradiance in the limiting range, in order to focus on changes in C metabolism and minimize superimposed responses linked with activation and relaxation of energy dissipation mechanisms that occur in shifts between oversaturating and very low irradiance (see Stitt et al. 1989). Plants were grown at 550 *µ*mol photons m^−2^ s^−1^. In stable irradiation, net CO_2_ assimilation (*A_n_*) rose steeply up to about 800 *µ*mol photons m^−2^ s^−1^ and more gradually as irradiance was increased further (Supplementary Fig. S1A). Transitions were performed between 550 *µ*mol photons m^−2^ s^−1^, moderate irradiance (moderate light [ML]), and 160 *µ*mol photons m^−2^ s^−1^, low irradiance (LL). Steady-state *A_n_* at ML and LL was about 25 and 10 *µ*mol CO_2_ m^−2^ s^−1^, respectively, or 135 and 53 nmol CO_2_ g^−1^ FW s^−1^, (estimated using a specific leaf area of 0.0055 m^−2^ g^−1^; Medeiros et al. 2022), corresponding to about 74% and 29%, respectively, of the maximum rate of photosynthesis (Supplementary Fig. S1A). On the day of the experiment, plants were illuminated at growth irradiance (=ML) for at least 4 h and then transferred to 160 *µ*mol photons m^−2^ s^−1^ (ML–LL) or were illuminated from the start of the light period at 160 *µ*mol photons m^−2^ s^−1^ for at least 4 h before transfer to 550 *µ*mol photons m^−2^ s^−1^ (LL–ML). Gas exchange parameters were logged every second, starting 15 min before and continuing until 30 min after the light transition. As gas exchange measurements shortly after light transitions strongly violate the steady-state assumption underlying default rate equations, dynamic equations were implemented (Saathoff and Welles 2021). Figure 1, A and B shows the response of *A_n_* with time on a log scale (see Supplementary Fig. S1, B and F, for a linear time scale and Supplementary Data Set 1 for data).

In the ML–LL transition (Fig. 1A), *A_n_* started to fall at 4 s, was significantly lower at 5 s (*P* = 0.006, Student's t-test), continued to decline until ∼250 s, and then recovered slightly until 1,800 s (Fig. 1C). The initial decrease was quasi-exponential with a t_0.5_ of about 11 s (Supplementary Fig. S1C). After 5, 10, and 15 s in LL, *A_n_* was 80%, 53%, and 44%, respectively, of that in ML, compared to a value of 40% at the end of the transition. A slow initial decay of *A_n_* was also seen by Sales et al. (2025) in maize after a decrease in irradiance in a 300 or a 30 s fluctuating light regime. At the trough, *A_n_* was ∼6% below the steady-state rate in LL. The subsequent recovery was significant (*P* = 0.002, paired *t*-test, legend of Fig. 1C) and equivalent to 7.6% of Δ*A_n_* (the difference between *A_n_* in ML and *A_n_* after 1,800 s in LL). Stomatal conductance (*g_s_*) responded more slowly, and internal CO_2_ concentration (*Ci*) rose by 190% to a peak at ∼30 s and then declined until ∼700 s, when *Ci* was 35% higher than in ML (Supplementary Fig. S1, D and E).

In the LL–ML treatment (Fig. 1B; Supplementary Fig. S1F), *A_n_* showed no significant change for 5 s. An initial fast rise starting at 6 s (20% above LL, *P* = 0.003, Student's *t*-test) was followed by a plateau between ∼11 and 15 s, a further rise until ∼90 s, and a further slow rise until the end of measurements. These phases were curve fitted (Supplementary Fig. S1G) and accounted for about 49%, 0$, 31% and 20% of the overall rise in *A_n_*, respectively. *g_s_* increased gradually between 30 and 360 s, and *Ci* declined 3-fold to a minimum at 50 s before rising 1.7-fold in the next 240 s (Supplementary Fig. S1, H and I). Essentially, similar responses occurred during a 300 s light fluctuation at 25 °C in maize in Sales et al. (2025), except that the 5 s lag before *A_n_* started to rise was less apparent.

### Global analysis of metabolism

Underlying changes in metabolism were investigated in separately grown plants. Gas exchange was also measured in these plants. For technical reasons, the correction for time lag could not be performed, but the essential features of response of *A_n_* were reproduced (see Supplementary Text Section S1 and Fig. S1). Replicate samples were collected before (0 s) and 5, 10, 15, 30, 60, 120, 300, 600, 1,200 and 1,800 s after transfer to the new irradiance (*n* = 4, except for time 0 of LL–ML, *n* = 10). Analysis by LC–MS/MS, GC–MS, and enzymatic assay allowed quantification of 36 metabolites (Supplementary Data Set 2).

**Figure 2.**
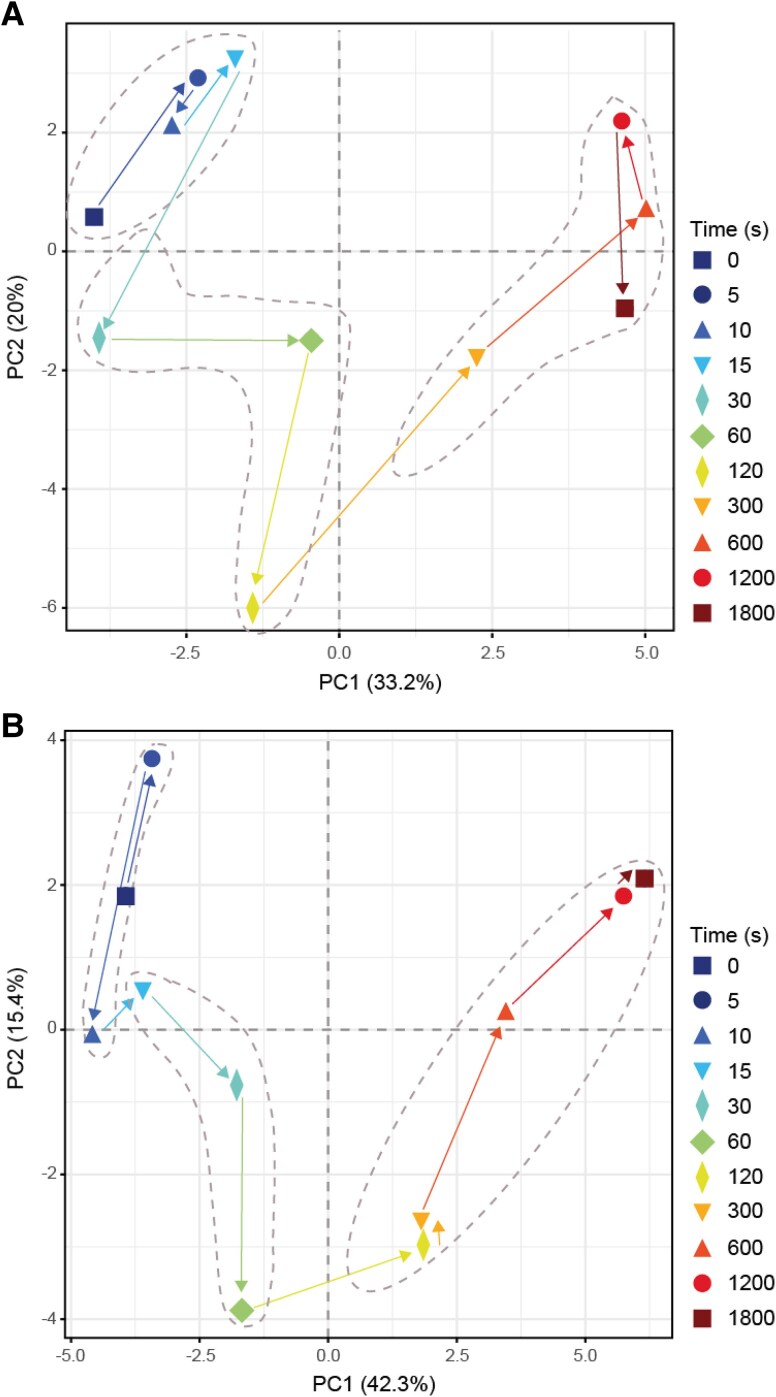
PC analysis. **A)** Analysis for a ML–LL transition and **B)** analysis for a LL–ML transition. The ML–LL and LL–ML experiments were performed 1 wk apart on separately grown batches of plants. Samples were collected in a randomized manner between 4 and 6 h into the light period. Means for each time point were used to perform the analyses (*n* = 4 to 5 for ML–LL and *n* = 4, except for time 0 s where *n* = 10 for LL–ML). The time points are identified by color and symbol (see insert), and the arrows show the time sequence in the transition. Different phases are indicated by dotted gray ellipses. Data are provided in Supplementary Data Set 2. Plots showing loadings of metabolites in PC1 and PC2 are shown in Supplementary Fig. S2.

To provide a global overview, we performed principal component (PC) analysis with all metabolites and *A_n_* (Fig. 2; Supplementary Fig. S2). In the ML–LL transition (Fig. 2A), PC1 and PC2 accounted for 33% and 20% of total variation, respectively. Visual inspection pointed to 3 sequential responses; an initial (0 to 15 s) shift to a more positive value in PC2 (time points 5, 10, and 15 s are close to each other), followed (15 to 120 s) by a large shift to negative values in PC2, followed (120 s onward) by a large shift to positive values in PC1 and reversal of the changes in PC2. The first phase corresponds to the initial delayed decrease of *A_n_*, the second phase mainly to the continuing slow decline of *A_n_* that was completed by 250 s, and the third phase to the subsequent small recovery of *A_n_* (see Fig. 1C and Supplementary Fig. S1L). PC1 differentiated steady-state metabolite profiles in ML and LL and captured gradual changes during adjustment to LL, while PC2 captured transient changes. Inspection of loadings (Supplementary Fig. S2A) reveals that PC1 was driven by intermediates in the CBC (mainly ribulose 5-phosphate + xylulose 5-phosphate [Ru5P + Xu5P], ribose 5-phosphate [R5P], and ribulose 1,5-bisphosphate [RuBP]), starch synthesis (ADP-glucose [ADPG]), the CCM (pyruvate, alanine) and photorespiration (glycine, glycerate) as well as 2-oxoglutarate (2OG), all of which align with *A_n_*, and by contrasting responses of aspartate, glutamate, and fumarate. There was no large contribution from fructose 1,6-bisphosphate (FBP) or sedoheptulose 1,7-bisphosphate (SBP). PC2 was mainly driven by 3PGA, sedoheptulose 7-phosphate (S7P), fructose 6-phosphate (F6P), Ru5P + Xu5P, and ADPG, which align with *A_n_*, and reciprocal changes of FBP, SBP, RuBP, 2OG, and alanine. It should be noted that the response of malate is not informative, because most of the malate in maize leaves is not involved in C_4_ photosynthesis, including pools in the vacuole, nonphotosynthetic cells, and midrib (Leegood 1985; Stitt and Heldt 1985a; Weissmann et al. 2016; Arrivault et al. 2017). These mask any changes in the malate pool that is directly involved in the CCM.

In the LL–ML shift (Fig. 2B), PC1 and PC2 accounted for 42% and 15% of total variation, respectively. Three sequential responses could be distinguished. The first (between 0 and 5 s) (less well-defined as it depends on 1 time point) was associated with a shift to more positive values in PC2, the second (5 to 60 s) by a large shift to negative values in PC2, and the third (60 s onward) by a strong shift to positive values in PC1 and a reversal of the changes in PC2. The first phase (0 to 5 s) corresponds with the time where *A_n_* did not change. The second phase corresponds to the rapid rise of *A_n_* between ∼6 and 11 s, the plateau from 11 to 15 s (time points 10 and 15 s are close to each other), and the subsequent rise until ∼90 s. The third phase corresponds to the slow subsequent rise in *A_n_* (see Fig. 1B and Supplementary Fig. S1G). PC1 again differentiated steady-state metabolite profiles in LL and ML and captured gradual changes during the adjustment to ML, whike PC2 captured transient changes. Inspection of loadings (Supplementary Fig. S2B) revealed that PC1 was driven by changes of most CBC intermediates (3PGA, DHAP, SBP, S7P, R5P, Ru5P + Xu5P, RuBP), some CCM intermediates (pyruvate, alanine, PEP), glycine, and 2OG, which all aligned with *A_n_*, and contrasting responses of F6P, 2PG, aspartate, glutamate, and fumarate and to a lesser extent F6P. PC2 was mainly driven by glucose 6-phosphate (G6P), sugars and serine, and contrasting changes of 3PGA, RuBP, FBP, ADPG, PEP, pyruvate, glycerate, and 2PG.

### Individual metabolites and estimation of metabolite ratios and sizes of pathway pools

**Figure 3.**
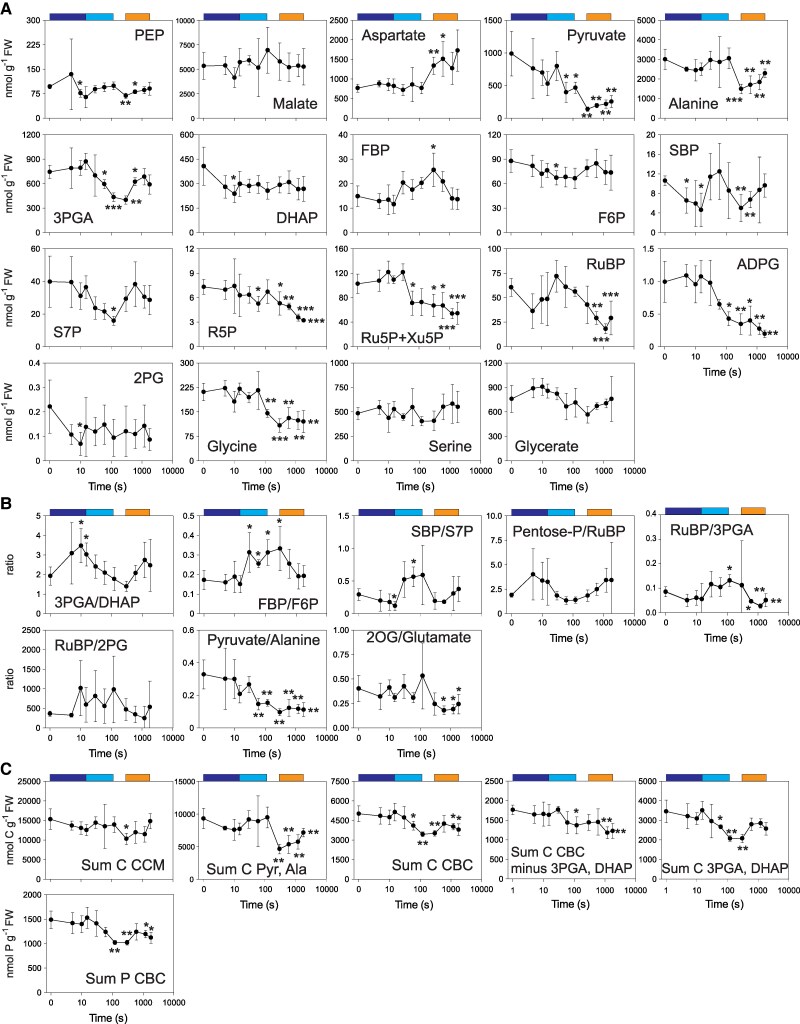
Changes of individual metabolites, metabolite ratios, and sets of metabolites in a ML–LL transition. **A)** Amounts of metabolites (nmol g^−1^ FW) involved in CCM, CBC (including ADPG), and photorespiratory pathway, **B)** ratios of metabolite amounts, and **C)** sum of C in CCM and sums of C and P in CBC (nmol C g^−1^ FW and nmol P g^−1^ FW). In **C)** metabolites summed for CCM were PEP, aspartate, pyruvate, and alanine (malate was not included because the active pool could not be defined). Data is shown as mean ± Sd (*n* = 4 to 5). Significant differences (*t*-test) to time zero (i.e. ML) are indicated by stars (*P < 0.05; **P < 0.01; ***P < 0.001). Time is shown on a log scale. The colored boxes above the graphs indicate the segments used to perform additional *t*-tests (Supplementary Fig. S3B). The original data are provided in Supplementary Data Set 2. Further metabolite levels are shown in Supplementary Fig. S3A.

**Figure 4.**
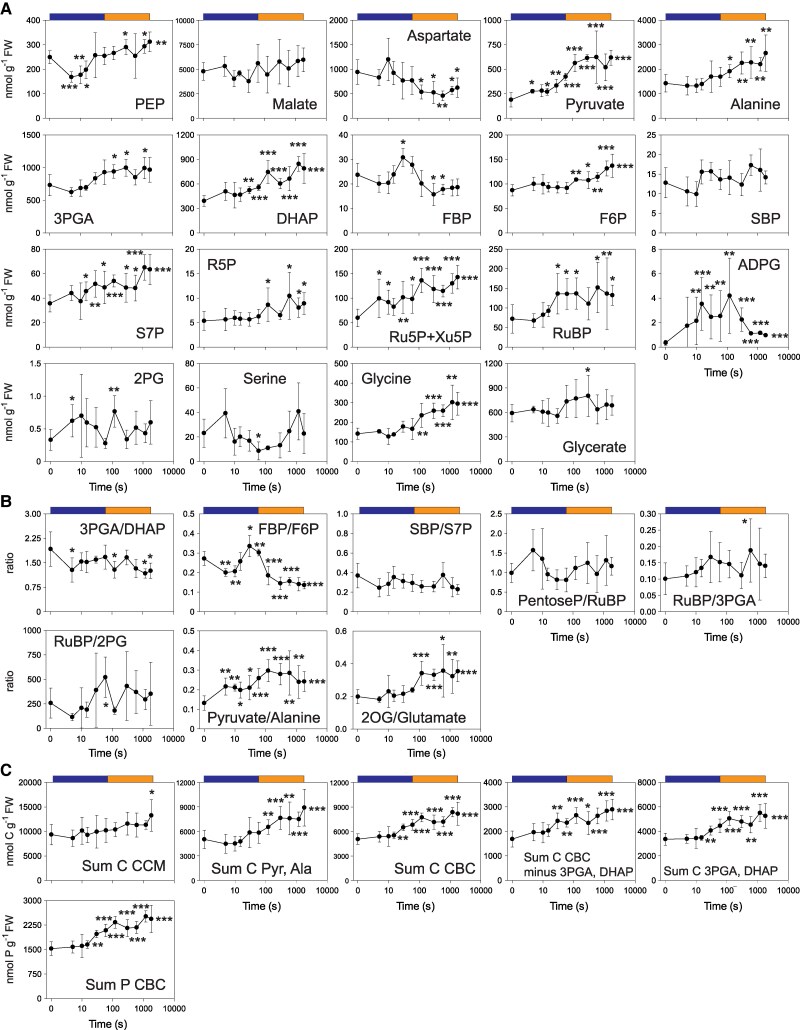
Changes of the levels of individual metabolite ratios and sets of metabolites in a LL–ML transition. **A)** Amounts of metabolites (nmol g^−1^ FW) involved in CCM, CBC (including ADPG), and photorespiratory pathway, **B)** ratios of metabolite amounts, and **C)** sum of C in CCM and sums of C and P in CBC (nmol C g^−1^ FW and nmol P g^−1^ FW). In **C)** metabolites summed for CCM were PEP, aspartate, pyruvate, and alanine (malate was not included because the active pool could not be defined). Data shown are means ± Sd (*n* = 4, except for time 0 s where *n* = 10). Significant differences (*t*-test) to time zero (i.e. ML) are indicated by stars (*P < 0.05; **P < 0.01; ***P < 0.001). Time is shown on a log scale. The colored boxes above the graphs indicate the segments used to perform additional *t*-tests (Supplementary Fig. S4B). The original data are provided in Supplementary Data Set 2. Further metabolite levels are shown in Supplementary Fig. S4A.

We next inspected the temporal responses of individual metabolites. Detailed time courses are shown for ML–LL in Fig. 3A and Supplementary Fig. S3A and for LL–ML in Fig. 4 and Supplementary Fig. S4A, and statistically significant changes are summarized in. Supplementary Figs. S3B and S4B. We tested for changes compared to time zero and for changes within later time intervals (defined from the PC analysis in Fig. 2). We did this because testing against zero might not capture important changes in later parts of the multiphasic responses.

We also investigated changes of metabolite ratios (Figs. 3B and 4B). These provide information about poising, the extent to which a particular reaction is restricting flux in an entire pathway. We estimated metabolite ratios for 3PGA/DHAP (indicative of the supply of NADPH and ATP; Dietz and Heber 1984), FBP/F6P, SBP/S7P, and pentose-P (sum of R5P, Ru5P + Xu5P)/RuBP (indicative of resistance to flux at FBPase, SBPase, and phosphoribulokinase, [PRK], respectively), and RuBP/3PGA and RuBP/2PG (indicative of resistance to flux at the Rubisco carboxylase and oxygenase reactions, respectively). We estimated pyruvate/alanine ratios, which provide indirect evidence about the contribution of NADP-ME and other decarboxylation routes, and the 2OG/glutamate ratio, which provides information about availability of amino groups and organic acid acceptors. In addition, we summed the amount of C in sets of metabolites that are involved in the CBC and in the CCM, as well as the amount of P in CBC intermediates (Figs. 3C and 4C). The CBC pool was also subdivided into 2 sets of metabolites: 3PGA and DHAP, which are involved in the energy shuttle, and intermediates involved in RuBP regeneration. Malate was excluded from the metabolites used to estimate the CCM pool for the reasons given above. Metabolite ratios and summed C in pathways are collectively referred to as “metabolic traits.”

There is scatter in the levels of several metabolites in C_4_ species including maize (see also Leegood and von Caemmerer 1988, 1989). We therefore performed further experiments in which we sampled larger numbers of replicates at 0, an early (10 s), and a late (1,200 s) time point after a ML–LL or a LL–ML transition (Supplementary Fig. S5, A and B, and Data Set 3). Most metabolites showed qualitatively consistent responses to those in the detailed time course, but a small number differed (Supplementary Fig. S5, C and D; in ML–LL, the RuBP/2PG ratio at 10 s and the RuBP/3PGA and RuBP/2PG ratios at 20 min; in LL–ML, ADPG at 10 s, FBP and SBP at 10 and 1,200 s, and fumarate, fructose, and raffinose at 1,200 s).

The next 2 sections present the temporal responses of metabolite levels and metabolic traits. This is done in the context of the phases identified in gas exchange and the PC analyses.

### Metabolic responses in the ML–LL transition

The first phase of the ML–LL response was from 5 to 15 s, corresponding to the delayed decline of *A_n_* (Fig. 1A). There was a slight increase in 3PGA, a large decrease (∼108 nmol·g^−1^ FW) in DHAP, and a large increase of the 3PGA/DHAP ratio (Fig. 3, A and B; significant for DHAP and the 3PGA/DHAP ratio; see Supplementary Fig. S3B), showing that substantial amounts of triose-P are oxidized after the drop in irradiance. Similar changes were seen by Sales et al. (2025) after a switch from high light to LL in a 300 s fluctuating light regime. There was a small significant decrease of SBP (Fig. 3A) and the SBP/S7P ratio (Fig. 3, A and B) but not of FBP or the FBP/F6P ratio. RuBP, total C and P in CBC intermediates (Fig. 3C), and ADPG remained high (Fig. 3A), consistent with CBC operation continuing for a short time after switching to LL. There was a rapid significant drop in 2PG (Fig. 3A; Supplementary Fig. S3B), the RuBP/2PG ratio remained unaltered or rose and the RuBP/3PGA ratio showed a nonsignificant decrease (Fig. 3B), consistent with slower RuBP oxygenation relative to carboxylation and, by implication, continued operation of the CCM. There were no significant changes in CCM metabolites except for a decline of PEP at 10 s, while pyruvate showed a nonsignificant downward trend.

The next phase of the ML–LL transition (until ∼120 s) corresponded to the further slow decline in *A_n_*. This was associated with a significant decrease of 3PGA (Fig. 3A; Supplementary Fig. S3B) and the 3PGA/DHAP ratio (Fig. 3B; Supplementary Fig. S3B; see analysis for the 15 to 120 s time segment), significant increases of the FBP/F6P and SBP/S7P ratios (Fig. 3B; Supplementary Fig. S3B), a significant decline of S7P and pentose-P, a significant decline in ADPG (Fig. 3A; Supplementary Fig. S3B), and a significant decline in total C and P in CBC intermediates (Fig. 3C; Supplementary Fig. S3B). RuBP levels showed a nonsignificant decline before recovering to a level like that in ML. Pyruvate declined significantly, but PEP, alanine, and the summed CCM metabolite pool initially remained high, trended downward by 120 s, and were significantly lower by 300 s (Fig. 3, A and C). In photorespiration, 2PG remained low, the RuBP/2PG ratio was high, and glycine started to decline (Fig. 3A; Supplementary Fig. S3B). Overall, CBC flux falls due to a shortfall of energy and, possibly, inactivation of CBC enzymes, and C is drained from the CBC and energy shuttle and, after a delay, from the CCM.

The third phase corresponded to the small but significant recovery of *A_n_* from ∼250 s onward. There was an increase of 3PGA and the 3PGA/DHAP ratio (Fig. 3, A and B; significant when tested in this phase; Supplementary Fig. S3B), a decrease of the FBP/F6P ratio (significant when tested in this phase), and a significant decrease of pentose-P, RuBP, and the RuBP/3PGA ratio (Fig. 3B; Supplementary Fig. S3B), while total C and P in CBC intermediates rose slightly (Fig. 3C; significant when tested in this time segment; Supplementary Fig. S3B). Pyruvate and alanine decreased between 120 and 300 s and then rose. Aspartate, which did not change in the first part of the transient, increased markedly from 300 s onward (Fig. 3A; significant when tested in this phase; Supplementary Fig. S3B). Fumarate increased (Supplementary Fig. S3A; significant from 300 s onward). As malate and fumarate are interconverted in a reversible reaction catalyzed by fumarase (see also Weissmann et al. 2016), this provides indirect evidence for an increase of a metabolically active malate pool. There was a marked and significant decrease in the 2OG/glutamate ratio (Fig. 3B; Supplementary Fig. S3B). Overall, the slight recovery of *A_n_* was associated with small adjustments in the CBC, a decrease of RuBP, and an increase of pools in the energy shuttle and CCM.

### Metabolic responses in the LL–ML transition

After increase in irradiance, rapid metabolic adjustments occurred within 5 s, before any detectable increase of *A_n_* (Fig. 1B). There was a trend to lower 3PGA, a significant rise in DHAP, and a significant decrease of the 3PGA/DHAP ratio (Fig. 4, A and B; Supplementary Fig. S4B), reflecting an almost immediate increase in availability of ATP and NADPH for 3PGA reduction. There was a significant decrease in the FBP/F6P ratio (Fig. 4B) due to small nonsignificant reciprocal changes of FBP and F6P (Fig. 4A). Reciprocal changes of DHAP and FBP may point to a restriction at aldolase or to complexities introduced by intercellular compartmentation (see Discussion). In the CCM, there was a significant ∼40% decrease in PEP and a significant increase in pyruvate (Fig. 4A; Supplementary Fig. S4B), pointing to increased PEPC activity, faster decarboxylation by NADP-ME, and a temporary restriction of flux at PPDK.

The second phase defined by PC analysis corresponded to the initial rapid increase of *A_n_* from 5 s onward, the plateau between 11 and 15 s, and another rise of *A_n_* until 90 s (Fig. 1B). The initial rise of *A_n_* and subsequent plateau were not accompanied by marked changes in metabolite levels. The initial decline of the 3PGA/DHAP ratio started to revert by 10 s, reflecting increased use of light energy for CO_2_ assimilation. In the CCM, PEP remained low and pyruvate slightly elevated. Between 15 and 90 s, *A_n_* rose at an accelerating pace (Fig. 1B). Time point 90 s was not collected for metabolite analyses, but already by 60 s, there was a coordinated increase in the levels of many CBC metabolites including 3PGA, DHAP, FBP, F6P, S7P, pentose-P, and RuBP (Fig. 4A, all significant; see Supplementary Fig. S4B). There was a significant ∼2-fold increase in the sum of C and of P in CBC intermediates (Fig. 4C; Supplementary Fig. S4B). The FBP/F6P ratio rose at 15 to 30 and subsequently decreased, indicating a transient restriction at FBPase (Fig. 4B; Supplementary Fig. S4B). Together, these observations point to the increase in CBC flux being linked both to a general increase in the pool sizes of CBC intermediates and, possibly, activation of CBC enzymes.

By 15 s, there was a highly significant 10-fold increase in ADPG (Fig. 4A; Supplementary Fig. S4B) and a decrease in G6P (Supplementary Fig. S5; significant at 30 and 60 s; Supplementary Fig. S4B). In C_3_ species such as spinach and barley (Stitt et al. 1980, 1983; Gerhardt et al. 1987), changes in total G6P reflect the behavior of the cytosolic G6P pool because the majority of the G6P is located in the cytosol, while F6P is more evenly distributed between chloroplast and cytosol. Assuming a similar distribution in maize, the decline in total G6P is consistent with a decline of G6P in the cytosol in the MC where sucrose synthesis occurs (Furbank et al. 1985). This is consistent with a delay in activation of flux at cytosolic FBPase, which might explain the strong rise in ADPG. Any delay in activating end-product synthesis will support build-up of the CBC and CCM metabolite pools (see Discussion).

In the CCM, after initially declining, PEP started to recover from 15 s onward (Fig. 4A, highly significant; see Supplementary Fig. S4B). The response of PEP qualitatively resembled but was larger than that of 3PGA. Similar changes were seen under fluctuating light in Sales et al. (2025). The larger relative decrease of PEP will favor flux from 3PGA to PEP via phosphoglycerate mutase and enolase (see Supplementary Text Section S2 for more details). From 15 s onward, there was an increase in pyruvate and an upward trend for alanine (Fig. 4A; significant for pyruvate but not for alanine until 120 s; see Supplementary Fig. S4B). This points to increased movement of C from the CBC via PEP into CCM intermediate pools. The 2OG:glutamate ratio started to rise, mainly due to an increase in 2OG (Fig. 4B; Supplementary Fig. S4; changes until 60 s were nonsignificant but the trend is confirmed by significant changes at 120 s).

From 90 s onward, *A_n_* continued to rise although more slowly, with this phase contributing ∼20% of the total increase in *A_n_*. Within the CBC, there was a further significant rise of DHAP, F6P, and S7P but not of 3PGA or RuBP, while FBP declined (Fig. 4A; Supplementary Fig. S4B; resulting in a further small increase of summed C or P in CBC intermediates (Fig. 4C; Supplementary Fig. S4B). There was a progressive rise in G6P and UDPG (Supplementary Fig. S5; significant for both when tested in this time segment; Supplementary Fig. S4B), indicative of increased flux over the cytosolic FBPase to sucrose (see above). There was a 5-fold decrease in ADPG, indicative of slower starch synthesis (Fig. 4A). The most marked changes after 120 s were further significant increases in PEP, pyruvate, and alanine (Fig. 4A) and summed CCM metabolites (Fig. 4C; Supplementary Fig. S4B). There was no consistent change in 2PG levels or the RuBP/2PG ratio, but there was a progressive rise in glycine (Fig. 4, A and B).

### Correlation analysis of *A_n_* with metabolite levels and metabolic traits

**Figure 5.**
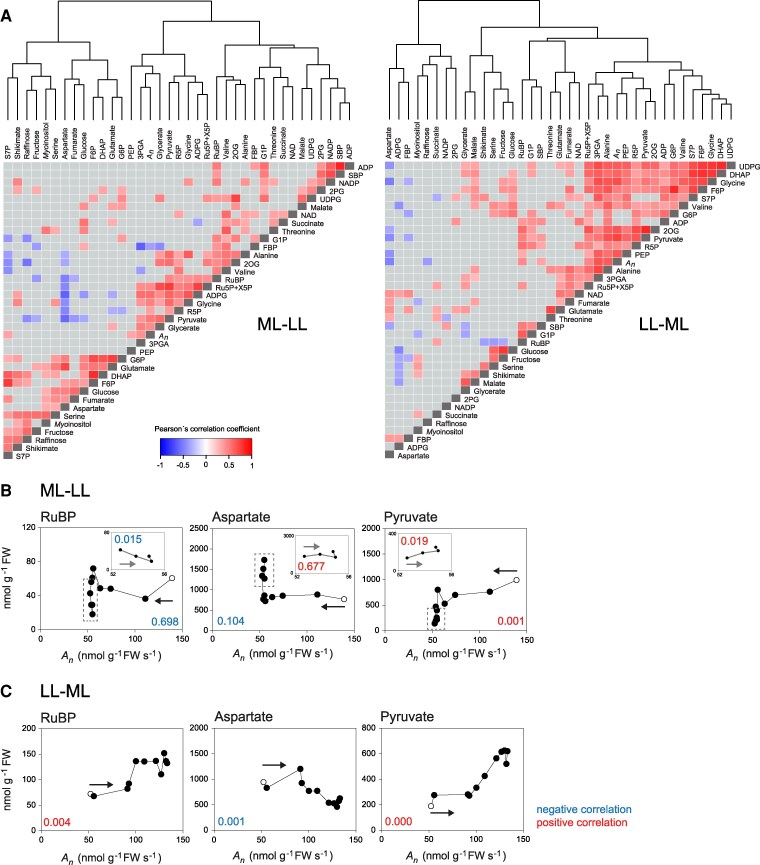
Correlations between metabolites and *A_n_.*  **A)** Heatmaps showing Pearson’s correlation coefficients for metabolites and CO_2_ assimilation (*A_n_*) in the ML–LL transition (left-hand side) and LL–ML transition (right-hand side). Pearson's correlation coefficients were calculated using individual samples at all time points after the transition to LL or the transition to ML (time zero excluded). *A_n_* was taken from Supplementary Data Set 1. Significant correlations (*P* < 0.05) are colored red (positive) and blue (negative) (see insert), while correlations that were not significant are shown in gray. Self-correlations are identified in dark gray. The hierarchical clustering is shown. Larger displays to aid identification of individual metabolites are provided in Supplementary Fig. S6, A and B, and a summary of R^2^ and *P* values in Supplementary Fig. S6C. **B)** Response of selected metabolites (*y* axis) in the ML–LL transition versus *A_n_* (*x* axis). **C)** Response of selected metabolites (*y* axis) in the LL–ML transition versus *A_n_* (*x* axis). For clearer visualization, the means are shown (*n* = 3 to 10). In **B)**, the open symbol is the value at ML, and the filled symbols are values after transition to LL, with the time sequence running from left to right (as indicated by a black arrow). The insert in each display shows time points between 300 and 1800 s (dotted gray box in the main panel), with an expanded scale for *A_n_* (52 to 56 *µ*mol CO_2_ g^−1^ FW s^−1^) to visualize correlations during the slight recovery of *A_n_* after 250 s (see Supplementary Fig. S1C). In the insert, the time sequence runs from right to left (indicated by a gray arrow). In **C)**, the open symbol is the value in LL, and the filled symbols are values after different times in ML, the time sequence runs from right to left (rising rate of photosynthesis with time). In both **A)** and **B)**, slope direction and *P* values were calculated by linear regressions using individual samples at all time points after the transition (time zero excluded) and in the case of **A)**, also from 300 to 1800 s (in insert). The *P* value is colored according to the slope direction (red and blue for positive and negative correlations, respectively). Additional responses of metabolites versus *A_n_* are provided in Supplementary Figs. S7A and S8A. The original data are provided in Supplementary Data Set 2.

**Figure 6.**
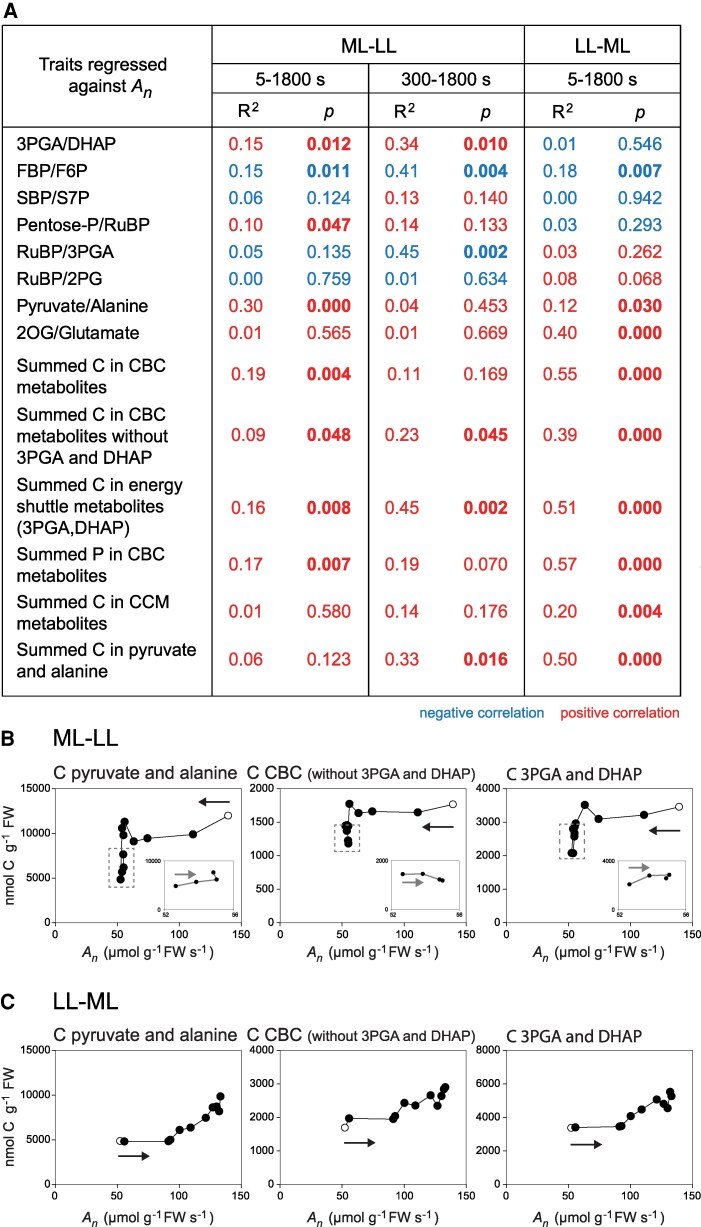
Correlations between metabolic traits and *A_n_*. **A)** Summary of R^2^, slope directions (slope d.), and *P* values. These were calculated by linear regressions using individual samples (*n* = 3 to 5) at all points after the transition (time zero excluded). Additional values were calculated from 300 to 1800 s for the ML–LL transition. Positive and negative correlations are indicated by color (red and blue, respectively). Those with significant *P* values (*P* < 0.05) are in bold. **B)** Response of summed C pools (*y* axis) in the ML–LL transition versus *A_n_* (*x* axis). **C)** Response of summed C pools (*y* axis) in the LL–ML transition versus *A_n_* (*x* axis). In **B)** and **C)**, C or P in the CBC is the sum of C or P in 3PGA, DHAP, FBP, F6P, SBP, S7P, R5P, Xu5P + Ru5P, and RuBP, summed C in CBC (without 3PGA and DHAP) is for all the above metabolites excluding 3PGA and DHAP, and summed C in the CCM is the sum of C in aspartate, pyruvate, alanine, and PEP (malate was excluded because the active pool could not be defined). Also shown is summed C in energy shuttle metabolites (3PGA and DHAP) and summed C in pyruvate and alanine as representative for the C in the CCM pool. These plots were made and presented as described in the legend of Fig. 5. Additional responses of metabolic traits versus *A_n_* are provided in Supplementary Figs. S7, B and C, and S8, B and C. The original data are provided in Supplementary Data Set 2.

**Figure 7.**
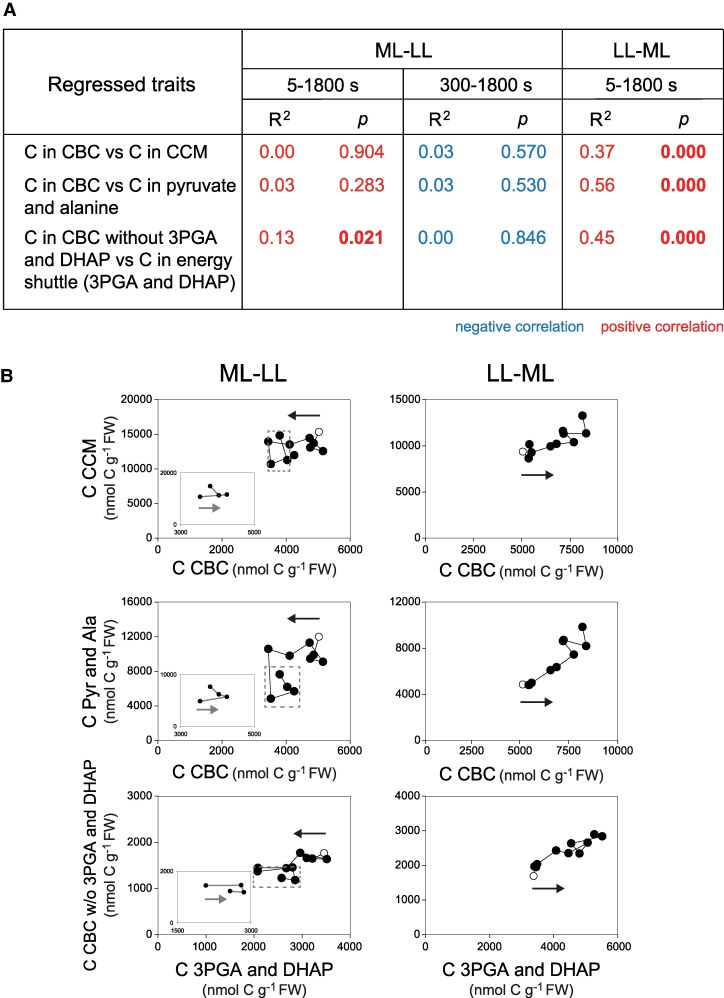
Correlations between C pools in the CBC and in the CCM. **A)** Summary of R^2^, slope directions (slope d.), and *P* values. These were calculated by linear regressions using individual samples (*n* = 3 to 5) at all points after the transition (time zero excluded). Additional values were calculated from 300 to 1800 s for the ML–LL transition. Positive and negative correlations are indicated by color (red and blue, respectively). Those with significant *P* values (*P* < 0.05) are in bold. **B)** Individual regression plots in the ML–LL and LL–ML transitions. These plots were done and presented as described in the legend of Fig. 5. The original data are provided in Supplementary Data Set 2.

The above analysis pointed to the metabolic responses in the transitions involving a combination of gradual and transient changes. To further explore the responses, we performed hierarchical clustering and determined Pearson’s correlation coefficients across each transition to search for sets of metabolites that change in a coordinated way and, in particular, to detect metabolites that correlate with *A_n_* (see Fig. 5A for an overview and Supplementary Fig. S6, A and B, for expanded plots). Correlation analyses were also performed between *A_n_* and metabolic traits (Fig. 6A). Plots of the relationship between *A_n_* and selected metabolites or metabolic traits are provided in Figs. 5, B and C, and 6B, and plots for all metabolites and metabolic traits are provided in Supplementary Figs. S7, B and C, and S8, B and C. We also compared selected metabolic traits with each other (Fig. 7). All of these statistical analyses focused on adjustment to a new irradiance and excluded time zero (ie the value before changing the irradiance).

In the ML–LL transition, a small subset of CBC intermediates (3PGA, R5P, pentose-P), the starch synthesis intermediate ADPG, 2 photorespiratory intermediates (glycine, glycerate), and 2 CCM intermediates (PEP, pyruvate) clustered together, correlated significantly with each other, and also clustered with and correlated significantly with *A_n_* (Fig. 5A; Supplementary Figs. S6, A and C, and S7, B and C). Most metabolites in this small cluster had strong loadings in PC1 of the PC analysis (Supplementary Fig. S2A). RuBP was only loosely associated, and DHAP, FBP, and SBP clustered elsewhere. *A_n_* was significantly and positively correlated with the 3PGA/DHAP ratio (Fig. 6A), pointing to the balance between the supply and consumption of NADPH and/or ATP changing in favor of energy consumption during the adjustment to low irradiance. *A_n_* was positively and significantly correlated with summed C and summed P in CBC intermediates and the energy shuttle intermediates (3PGA, DHAP) as well as with summed C in other CBC metabolites (Fig. 6A). However, *A_n_* was unrelated to summed C in CCM intermediates or summed C in pyruvate plus alanine. Furthermore, summed C in CBC intermediates changed independently of summed C in CCM intermediates or summed C in pyruvate plus alanine (Fig. 7). Aspartate showed negative correlations with *A_n_* and many metabolites (Fig. 5A; Supplementary Fig. S5A)

Individual plots of *A_n_* against individual metabolites or metabolite traits also revealed rather diverse responses in the ML–LL transition (see Fig. 6A and Supplementary Fig. S6C for a summary and statistical analysis and inserts in Figs. 5B and 6B and Supplementary Fig. S7 for the plots). This was especially so when the response during the fall of *A_n_*, until ∼300 s, is compared with the response during the slight recovery of *A_n_* from 300 s onward. As *A_n_* fell, most metabolites declined (ie correlated positively with *A_n_*). During the slight recovery, some metabolites rose (ie correlated positively with *A_n_*), but many declined (ie correlated negatively with *A_n_*). There was decrease of FBP and the FBP/F6P ratio, a decrease of RuBP and the RuBP/3PGA ratio, a decline of summed C in CBC metabolites when 3PGA and DHAP were excluded (Figs. 5B and 6B), and an increase of the 3PGA/DHAP ratio. There was an increase of summed C in energy shuttle metabolites (Fig. 6B), and of PEP (Supplementary Fig. S7A), alanine (Supplementary Fig. S7A), summed C in pyruvate plus alanine (Fig. 6B), and summed C in all CCM intermediates (Supplementary Fig. S7C) and, after a decrease until 300 s, a slight recovery of pyruvate (Fig. 5B). Many of these changes were significant when tested in the 300 to 1,800 s time segment (Supplementary Fig. S6C) or when 600, 1,200 and 1,800 s were individually tested against 300 s (Supplementary Fig. S3B). As *A_n_* rose from 39.7 to 41.8 nmol CO_2_ g^−1^ FW s^−1^ (Supplementary Fig. S1C), energy shuttle and CCM pools increased by ∼4,760 nmol C g^−1^ FW s^−1^. This is equivalent to ∼90 s of photosynthesis in LL, ∼58 s of the difference in photosynthesis between ML and LL (Δ*A_n_*), ∼10% of the CO_2_ fixed during the entire recovery, and more in the first 900 to 1,000 s by when the recovery was largely completed.

A differing picture emerged for the LL–ML transition, where a much larger set of CBC (3PGA, DHAP, F6P, S7P, pentose-P), CCM (PEP, pyruvate, alanine), photorespiratory (glycine) intermediates, and 2OG clustered together, correlated significantly with each other, and also clustered with and correlated significantly with *A_n_* (Fig. 5A; Supplementary Fig. S6B). These metabolites had strong loadings in PC1 of the PC analysis (Supplementary Fig. S2B). There was a slight separation of RuBP, and SBP, and, especially, FBP clustered elsewhere.

Plots of selected metabolites and metabolic traits against *A_n_* (Fig. 5C; Supplementary Fig. S8, A to C; see Supplementary S6C for a summary) revealed a progressive increase in the level of many metabolites as *A_n_* rose in the LL–ML transition. Notable exceptions included RuBP that plateaued in the last half of the rise in *A_n_* (Fig. 5C; Supplementary Fig. S7A), 2PG that showed a rapid initial rise and subsequent fluctuations (Fig. 4A), and FBP and SBP that changed independently of *A_n_* (Supplementary Fig. S8A). The FBP/F6P ratio and SBP/S7P ratio rose to a maximum at an intermediate *A_n_* of 75 to 89 nmol CO_2_ g^−1^ FW s^−1^ and then declined as *A_n_* rose further (Supplementary Fig. S8B; significant for the FBP/F6P ratio; see Supplementary Fig. S4B). This points to FBPase and, possibly, SBPase initially restricting CBC flux and this restriction being subsequently relaxed. There was a weak positive correlation between *A_n_* and the RuBP/2PG ratio in the total transient, but somewhat above the significance cut-off (Fig. 6A; *P* = 0.068); for plot see Supplementary Fig. S8B). Aspartate showed a progressive decrease as *A_n_* rose (Fig. 5C) and was negatively correlated with many metabolites (Fig. 5A; Supplementary Fig. S6, B and C).

As a consequence of the rise of many metabolites, *A_n_* was significantly positively correlated across the entire LL–ML transition with summed C in CBC intermediates, summed C in metabolites in the energy shuttle, summed C in pyruvate plus alanine, and summed C in CCM metabolites (malate was excluded for reason explained above) (Fig. 6A; see Fig. 6B and Supplementary Fig. S8C for more plots and Supplementary Fig. S6C for a summary). Furthermore, summed CBC metabolites correlated strongly and positively with summed CCM metabolites and with summed pyruvate plus alanine. Summed CBC metabolites excluding 3PGA and DHAP also correlated strongly with summed 3PGA plus DHAP (Fig. 7). The rather coordinated increase in CBC, energy shuttle, and CCM pool sizes in the LL–ML transition contrasts with the less coordinated response in the ML–LL transition.

There were nevertheless differences of emphasis between the early and later parts of the LL–ML transition. As *A_n_* rose to 87 nmol CO_2_ g^−1^ FW s^−1^, RuBP and metabolites involved in RuBP regeneration rose (Fig. 6C). As *A_n_* rose further to 130 nmol CO_2_ g^−1^ FW s^−1^, CBC intermediates fluctuated but did not shown any general increase and RuBP decreased (Figs. 4A and 5C). In contrast, metabolites involved in the energy shuttle (3PGA, DHAP) showed little change as *A_n_* rose to 87 nmol CO_2_ g^−1^ FW s^−1^ but increased markedly as *A_n_* rose further (Fig. 6C; Supplementary Fig. S8, A and C). The pyruvate and alanine pool rose slightly as *A_n_* rose to 87 nmol CO_2_ g^−1^ FW s^−1^ but rose strongly as *A_n_* rose further (Fig. 6C). Thus, the first part of the rise of *A_n_* was mainly associated with build-up of CBC pools involved in RuBP regeneration and the second part with build-up of the energy shuttle and CCM pools.

Overall, the rise of *A_n_* from about 50 to 130 nmol CO_2_ g^−1^ FW s^−1^ was accompanied by an increase of summed C in the CBC, energy shuttle, and CCM of about 7,000 nmol C g^−1^ FW. This is equivalent to about 54 s of *A_n_* in ML and considerably more of the lower *A_n_* that prevailed for the first part of the transition.

Some trends were common to the ML–LL and LL–ML transitions (Fig. 6). There was a strong negative correlation of aspartate and positive correlation of the pyruvate/alanine ratio with *A_n_* in both transitions, consistent with the contribution of NADP-ME relative to other decarboxylases declining in low irradiance and increasing in high irradiance, with this shift occurring gradually over the entire transition (see Figs. 3 and 4 and Supplementary Figs. S7 and S8). The 2OG/glutamate ratio was strongly positively correlated with *A_n_* in the LL–ML transition and showed a weak positive trend with *A_n_* in the ML–LL transition, pointing to the balance between amino and carboxylic groups changing during these transitions.

## Discussion

### Response of C_4_ metabolism to changes in irradiance is slow, complex and uncoordinated

C_4_ photosynthesis faces multiple challenges under changing irradiation, including coordinating fluxes in the CCM and the CBC, balancing energy provision and consumption in 2 cell types, and adjusting the size of the large metabolite pools that drive intercellular shuttles. To better understand their impact, we carried out time-resolved measurements of *A_n_* and metabolites in maize leaves after a sudden increase or decrease in irradiance. *A_n_* showed a multiphasic response in both transitions. PC analysis of the global metabolite response also revealed sequential phases, which matched major features in the response of *A_n_*. These phases were further investigated by scrutinizing the responses of individual metabolites and metabolic traits such as metabolite ratios and summed pools in the energy shuttle and the CCM (Figs. 3 and 4; Supplementary Figs. S3 and S4) and by analyzing relationships between *A_n_* and metabolite levels or metabolic traits (Figs. 5 and 6; Supplementary Figs. S6 to S8).

**Figure 8.**
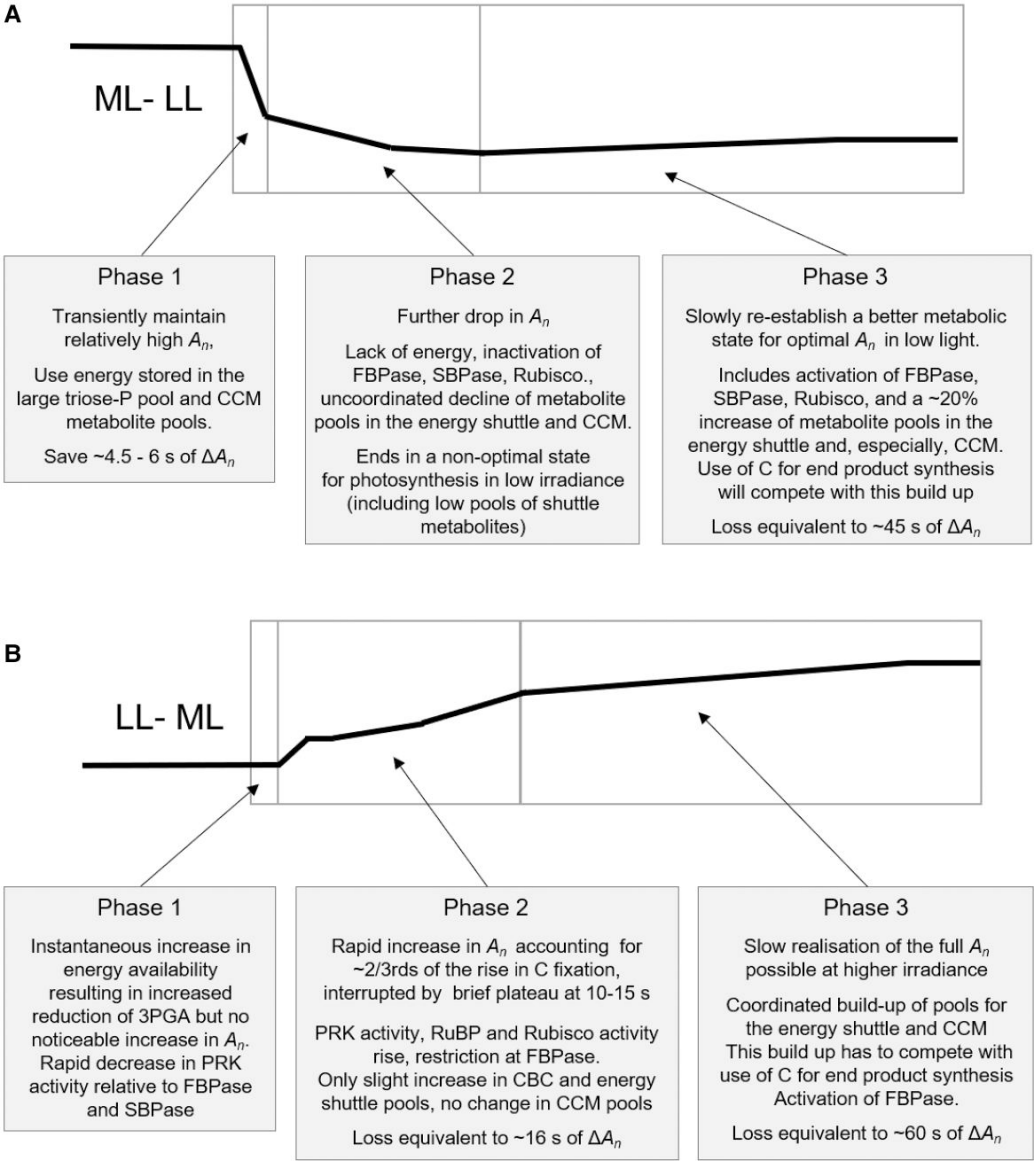
Schematic summary of the adjustment of C_4_ photosynthesis in maize to a change in irradiance in the nonsaturating range. **A)** Adjustment to a decrease in irradiance and **B)** adjustment to an increase in irradiance: The displays show schematically the change of *A_n_* in each phase, and the boxes summarize the main accompanying changes in metabolism. The loss of *A_n_* in a given phase was estimated as follows. In the ML–LL transition, *A_n_* above (Phase 1) or below (Phase 3) steady-state *A_n_* in LL was integrated over the phase and subtracted from the Δ*A_n_* (the difference between *A_n_* in ML and LL). In the LL–ML transition, *A_n_* below steady-state *A_n_* in ML was integrated over Phase 2 or Phase 3 and subtracted from Δ*A_n_*.

The responses are schematically summarized in Fig. 8. The response after a decrease in irradiance was especially complex (Fig. 8A). The main phases included transient buffering of photosynthesis by energy released by transformation of large pools of energy and CCM intermediates and a transition to a suboptimal state at ∼250 s due to overdepletion of energy shuttle and CCM pools, followed by slow partial recovery of *A_n_* as these pools gradually recovered. After an increase in irradiance (Fig. 8B), the increase in *A_n_* took several minutes including an initial short delay, then a short rise, a plateau, and a further rise until about 90 s *A_n_* that was associated with rapid build-up of CBC pools and possibly enzyme activation and a slower rise from 90 s onward that accounted for the last ∼20% of the gain in *A_n_* and was associated with gradual build-up of pools in the energy shuttle and CCM. Overall, our study provides experimental support for earlier, mainly theopoetical, ideas about what might decrease the efficiency of C_4_ photosynthesis during changes in irradiance. It also reveals that the responses of the CBC, energy shuttle, and the CCM are not only delayed but also in part uncoordinated.

### Response of *A_n_* in our study often resembles those in previous studies of light switches or fluctuating light

Our study was performed with the fourth fully elongated leaf of 3-wk-old maize growing in a controlled climate chamber. Leaf structure and responses to fluctuating light change with plant development, with leaves from older plants after the miR156-dependent vegetative phase change having a higher investment of biomass, a slower rise of *A_n_* after a switch from darkness to light, and a larger loss in C gain under strongly fluctuating light environments (Lawrence et al. 2022). Further, the leaves we used experienced no prior shading. This prehistory may affect the response to changes in irradiance; for example, PEPCK activity and flux through aspartate-based routes is lower in top leaves than in subtended leaves that experience fluctuating shade (Arp et al. 2021). We also investigated single transitions, rather than light fluctuations where the starting point for each adjustment depends on the frequency of the fluctuation (Arce Cubas et al. 2023a). Also, to reduce complications due to slow relaxation of energy dissipation, we worked in an irradiance range that was limiting for photosynthesis. Thus, the response of *A_n_* and the contribution of various metabolic factors in our standardized treatments cannot be automatically transferred to plants in the field. That said, they provide a starting point to interpret responses in the myriads of combinations of plant stage, prehistory, and light regime that occur in the field. Indeed, many aspects of the response of *A_n_* in our standardized transitions resemble those in published studies of sudden transitions or fluctuating light.

In earlier studies where irradiance was decreased, C assimilation typically continued for a time either after darkening (Laisk and Edwards 1998) or after a short light fleck (Krall and Pearcy 1993) or after the switch to LL in a fluctuating regime (Lee et al. 2022; Arce Cubas et al. 2023a; Sales et al. 2025). A trough of A_n_ followed by a slight recovery has been reported for a sudden high light to LL transition with maize (Doncaster et al. 1989) and in some but not all studies with fluctuating light (Li et al. 2021; Lee et al. 2022; Arce Cubas et al. 2023a; Sales et al. 2025). This may reflect differences in poising, but also whether the time in LL was long enough to capture the slow recovery.

In earlier studies where irradiance was increased, *A_n_* rose slowly over minutes, both after sudden dark–light transition (Furbank and Leegood 1984; Usuda 1985; Lawrence et al. 2022; Arce Cubas et al. 2023b) and after a sudden switch from low to high light (Kubásek et al. 2013). This response was often biphasic with a second slow component (Li et al. 2021; Lee et al. 2022) resembling that seen from 90 s onward in our LL–ML transition. In a detailed study of maize in fluctuating light, Sales et al. (2025) observed a similar 2-phase response of *A_n_* after switching to high light in a 300 s fluctuating regime. Earlier studies also reported complex transients immediately after a switch from darkness to light (Laisk and Edwards 1998) or a switch from low to high light (Krall and Pearcy 1993), or under fluctuating light including an interruption at about 10 s, which in fluctuating light persisted until almost 30 s (Sales et al. 2025), or to a plateau after about 60 s (Lee et al. 2022; Arce Cubas et al. 2023a).

The main metabolic events during the response of *A_n_* in our ML–LL and LL–ML transitions are discussed in more detail the following and in Supplementary Text Sections S2 to S6. They include changes in metabolite pool sizes and possible changes in enzyme activation inferred from changes in metabolite levels. Although the inferred changes in enzyme activation were not directly confirmed by direct measurements, in some cases, they match changes in enzyme activation reported in earlier studies. A major feature in both transitions is net movement of C between the CCM pool and the energy shuttle and CBC intermediate pools, or vice versa. This involves flux between 3PGA and PEP, via the near-equilibrium reactions catalyzed by phosphoglycerate mutase and enolase. A detailed theoretical analysis is provided in Supplementary Text (Section S2).

### Response of the CBC and CCM to a sudden decrease in irradiance

Our results and those of Sales et al. (2025) under fluctuating light confirm the prediction of Stitt and Zhu (2014) that the large pools of metabolites in the energy shuttle and the CCM temporarily buffer C_4_ photosynthesis against a sudden shortfall of ATP and NADPH from the light reactions. Crucially, the observed changes in metabolite levels in our study show, firstly, that CBC operation continues for a time and, secondly, that the decline of DHAP was quantitatively large enough to supply the energy required to support the temporarily elevated rate of CO_2_ fixation over that in steady-state LL (for details, see Supplementary Text Section S3.1).

The subsequent fall in *A_n_* was primarily due to a shortfall in energy and was accompanied by a general decline of CBC intermediates; changes of metabolites indicative of decreased activation of FBPase, SBPase, and Rubisco; and a decline of the metabolite pool in the energy shuttle as well as a decline of pyruvate due to loss of C from the CCM pools (for details, see Supplementary Text Sections S2.2 and S3.1). It ended after ∼200 to 250 s with a transient trough in *A_n_*. This was followed by a slow partial recovery of *A_n_*. Several factors might, in principle, contribute to the transient trough and subsequent partial recovery of *A_n_*.

One possibility is that the decline in energy availability and CO_2_ fixation leads to depletion or suboptimal poising of CBC intermediates and/or inactivation of enzymes and that this is corrected during the partial recovery. However, inspection of changes in metabolite levels and metabolite ratios indicates that, except for 3PGA and DHAP, CBC metabolites do not rise and that RuBP even declines during the partial recovery of *A_n_* (Figs. 3 and 6, A and B; Supplementary Figs. S3B, S6C, and S7A). It is anyway unlikely that adjustments within the CBC make a major contribution to the slow recovery of *A_n_*. As in C_3_ photosynthesis, in maize, the CBC metabolite pools are small and turn over rapidly (Arrivault et al. 2017) with the exception of 3PGA and DHAP that are involved in the energy shuttle, and activation of CBC enzymes adjusts in a relatively short time (Doncaster et al. 1989).

Another possibility would be that the trough and gradual recovery is due to slow relaxation of energy dissipation. However, this seems unlikely because the experiments were carried out in the limiting light range where large changes in energy dissipation are unlikely (see also Sales et al. 2025). Furthermore, the 3PGA/DHAP ratio rose during the recovery (Fig. 3B; Supplementary Figs. S3B and S6C) rather than falling as would be expected if delivery of ATP and NADPH from the light reactions was increasing. The recovery also cannot be due to slow adjustment of stomatal conductance, which together with *C_i_* decreased in this time (Supplementary Fig. S1D).

Based on these prior considerations, the major explanation for the trough of *A_n_* seems to be that, during the preceding decline of *A_n_*, metabolite pools in the energy shuttle and, especially, the CCM decline below those required for optimal operation at the new low irradiance. The decline in CBC metabolite levels is accompanied by an especially large decline of the energy shuttle intermediates 3PGA and DHAP (Fig. 3A). As the CBC and CCM pools are connected via the near-equilibrium reactions of phosphoglycerate kinase and enolase, low 3PGA will in turn divert PEP away from PEPC, draining C from the CCM (Fig. 3, A and C). This explanation is supported by the observed decrease of the 3PGA/PEP ratio at 60 to 120 s, coinciding with depletion of the CCM pool (for more details, see Supplementary Text Sections S2.2 and S3.1). Furthermore, metabolite pools drive intercellular shuttles less efficiently in low irradiance than high irradiance (Medeiros et al. 2022). This will further exacerbate the impact on *A_n_* of depletion of these pools after a sudden decrease in irradiance.

The subsequent gradual recovery of *A_n_* was accompanied by a rise in the level of energy shuttle intermediates (Figs. 3A and 6B) and a somewhat delayed rise in the levels of pyruvate, alanine, aspartate (Fig. 3A; Supplementary Fig. S7A), and total C in CCM metabolites (Fig. 3C; Supplementary Fig. S7C; for significance, see Fig. 6A and Supplementary Figs. S3B and S6C). Overall, the C that accumulated in the CBC, energy, and CCM pools was equivalent to about 10% of the CO_2_ fixed during the recovery phase, and a larger proportion of the CO_2_ fixed in the first part of the recovery, with most of this being due to build-up of CCM pools. Incidentally, this gradual improvement in operation of the CCM in low irradiance provides an explanation for why *A_n_* rises even though RuBP, stomatal conductance, and *C_i_* are decreasing (for details, see Supplementary Text Section S3.1).

The overdepletion and slow recovery of the energy shuttle and CCM pools in the ML–LL transition is not due to large pool size per se. It is a consequence of how these large pools impact poising of C gain with end-product synthesis. Due to the topology of the CBC, use of fixed C for end-product synthesis must be tightly regulated to maintain CBC intermediates in a concentration range that allows rapid regeneration of RuBP (Edwards and Walker 1983; Stitt et al. 2010, 2021; Heldt and Piechulla, 2021). This can be illustrated by considering synthesis of sucrose, the main end-product of photosynthesis. In C_3_ plants, triose-P is exported from the plastids to the cytosol, where it is converted to sucrose. Fructose 2,6-bisphosphate (Fru2,6BP) interacts with other metabolites in a highly cooperative network to strongly restrict cytosolic FBPase activity in response to a small decline in the level of triose-P (Herzog et al. 1984; Stitt 1990; Stitt et al. 2010, 2021). After a decrease in irradiance, this network allows rapid inhibition of triose-P consumption for sucrose synthesis while maintaining CBC metabolites at relatively high levels (Stitt et al. 1983, 2021; Stitt 1990). In maize, sucrose is synthesized in the cytosol of the MC (Furbank et al. 1985; Furbank and Kelly 2021), and this process must be coordinated with maintenance of high triose-P concentrations in the MC to drive their diffusion back to the BSC (Leegood 1985; Stitt and Heldt 1985a; Arrivault et al. 2017). High triose-P concentrations can be maintained in the MC because, compared to the C_3_ cytosolic F1,6BPase, the maize enzyme has a circa 10-fold higher K_m_ for FBP and is more sensitive to inhibition by Fru2,6BP (Stitt and Heldt 1985b). Thus, while a decrease in C fixation and the associated partial inhibition of sucrose synthesis is associated with a decrease in triose-P levels, this occurs in a much higher concentration range in maize than in C_3_ species (Stitt and Heldt 1985a, 1985b; compare also steady state DHAP levels in C_4_ and C_3_ plants in Arrivault et al. 2019). Crucially, the larger absolute size of the triose-P pool and associated pools such as 3PGA means that a given relative change in triose-P concentration is associated with a much larger change of absolute pool size in maize than in a C_3_ plant. Further, as 3PGA is linked via the reversible phosphoglycerate mutase and enolase reactions to PEP (Arrivault et al. 2017; see also Supplementary Text Section S2), any decline in triose-P and 3PGA leads to drainage of C from the large pools of CCM intermediates. Thus, when photosynthetic rate falls, any delay in restricting flux at cytosolic F1,6BPase will lead to a much larger absolute drainage of C from photosynthetic metabolism in a C_4_ plant such as maize than in a C_3_ plant. Consequently, it takes longer to reverse this process and build up the pools again.

In earlier experiments where maize was transferred from stable illumination at 1,700 to 144 *µ*mol m^−2^ s^−1^ (Doncaster et al. 1989), there was a slight recovery of *A_n_* from about 2 min onward. The trough and recovery were not observed in a 300 s fluctuating regime (Sales et al. 2025; but this may be because the LL phase was too short to see the recovery.

Taken together, immediately after transferring maize from stable high to low irradiance, higher *A_n_* is maintained than in steady-state LL. This is supported by energy from metabolic transformations within the pools of energy shuttle metabolites and, possibly, CCM metabolites. However, by 200 to 250 s, the metabolic network transitions into a state that is not optimally poised for effective use of the low irradiance, and it takes several minutes for a better poise to be established. A major contributor is overdepletion of the energy shuttle and CCM pools, due to slow inactivation of end-product synthesis, followed by their gradual recovery. The gain of C fixation due to maintaining *A_n_* immediately after reducing irradiance was equivalent to about 6 s of Δ*A_n_*, (the difference between *A_n_* in ML and LL) (Fig. 8A). This is more than canceled out by the loss of C fixation due to the trough and slow recovery, which was equivalent to about 45 s of Δ*A_n._* (Fig. 8A). Hence, while our experimental study confirms the prediction of Stitt and Zhu (2014) that large metabolite pools may temporarily buffer C_4_ photosynthesis against a sudden reduction in irradiance, it also reveals that there can be a larger loss of photosynthetic efficiency due to suboptimal responses during the subsequent adjustment to low irradiance. That said, under rapid light fluctuations that often predominate in dense C_4_ grass canopies, gains due to short-term buffering of *A_n_* will probably outweigh the losses that occur after a switch from high light to prolonged LL. This however assumes that optimal pool size for intercellular shuttles can be established and maintained under fluctuating light.

### Response of the CBC and CCM to an increase in irradiance

After an increase in irradiance, the 3PGA/DHAP ratio decreased within 5 s, as expected from the increased availability of NADPH and ATP (Fig. 4B; Supplementary Fig. S4B; see Sales et al. 2025 for a similar decrease in fluctuating light). *A_n_* started to rise by 6 s, followed by a transient plateau at around 11 to 15 s and a second rapid rise until 90 s. The rise in *A_n_* between 6 and 90 s was associated with an overall increase in CBC metabolite pools (Figs. 4A and 6C; Supplementary Figs. S4B and S6B) and transient changes indicative of enzyme regulation (see Supplementary Text Section S3.2). The interruption between ∼11 and 15 s by a transient plateau is probably linked to delayed activation of the CCM and CBC, including uncoordinated responses that may lead to transient back-leakage of CO_2_ (see Supplementary Text Sections S3.2 and S5). A similar plateau was also seen under fluctuating light (Sales et al. 2025).

From 90 s onward, *A_n_* continued to rise, but more slowly. This slow rise was accompanied by a rise of 3PGA and DHAP, PEP, pyruvate, and alanine, i.e. pools involved in the energy shuttle and the CCM. Indeed, *A_n_* correlated strongly with the increase in energy shuttle and especially CCM pool size (Fig. 6, A and C; Supplementary Figs. S4B and S8C and Text Section S2.2).

Overall, over 1,800 s, summed C in the CBC, energy shuttle, and CCM increased by ∼7,000 nmol C g^−1^ FW. This is equivalent to about 60 s of *A_n_* in ML and considerably more of the lower *A_n_* that prevailed in the first part of the transition (for more details, see Supplementary Text Section S3.2). During the adjustment, the sizes of the energy shuttle pool and CCM pool were strongly correlated (Figs. 4C, 6, and 7). Thus, neither the energy shuttle nor the CCM is prioritized when maize is adjusting to a sudden increase in irradiance (see also Supplementary Text Sections S2.2 and S3.2) in contrast to the uncoordinated response during the ML–LL transition.

The initial increase in CBC pool size and subsequent rise in energy shuttle and CCM pool size require tight regulation of end-product synthesis. G6P and UDPG fell until about 60 s, but then rose (Supplementary Fig. S4, A and B), pointing to an initial restriction at cytosolic FBPase that is subsequently relaxed, after which sucrose synthesis competes for newly fixed C with the build-up of pools in the energy shuttle and CCM. A somewhat delayed activation of the cytosolic FBPase is also seen in C_3_ species and is at least partly due to a short delay until the Fru2,6BP levels fall (Stitt 1990; Stitt et al. 2010, 2021). Incidentally, the initial increase and subsequent decrease of ADPG (Fig. 4A; Supplementary Fig. S4B) are consistent with a delay until flux at cytosolic FBPase increases.

In a study under fluctuating light, Li et al. (2021) observed that, after switching from low to high light, C_3_ species showed a rapid rise of *A_n_* within 1 min to a value similar to or only slightly lower than that attained in steady-state high light. In contrast, in C_4_ species, the response was biphasic with a rapid initial rise, followed by a much slower gradual increase. This pattern has been seen in many NADP-ME and NAD-ME species (see also Lee et al. 2022), although not in 2 PEPCK species (Lee et al. 2022; Arce Cubas et al. 2023a) (see Supplementary Text Section S3.2). In many cases, the steady-state rate in high light was not reached within 2 min, and in the maize LL–ML transition, full recovery required more than 8 min. Thus, the rapid initial rise and slower gradual rise seen in maize in a LL–ML transition (Fig. 1B; Supplementary Fig. S1, F and G) and by Sales et al. (2025) under fluctuating light is a general feature of NADP-ME and NAD-ME subtypes, although not necessarily PECK subtypes.

Wang et al. (2021) modeled the response of C_4_ photosynthesis after dark–light transition and predicted a relatively short delay until maximum *A_n_* was achieved, due to transient limitation by Rubisco activase, the PPDK regulatory protein, and stomatal conductance. While their model correctly predicted a slow build-up of energy shuttle and CCM pools, this continued long after reaching maximum *A_n_*. In experimental studies of dark–light transitions, Furbank and Leegood (1984) and Usuda (1985) observed a closer match between the increase of *A_n_* and the increase in 3PGA, DHAP, and metabolites such as pyruvate from the CCM. This resembles our study of a low to higher light transition. As noted by Wang et al. (2014a), parameterization of the resistance to intercellular movement of metabolites is challenging. It is possible that the parameterization in Wang et al. (2021) underestimated the resistance to intercellular movement of metabolites and, hence, underestimated the contribution of a slow build-up of metabolite pools to the response of *A_n_* after an increase in irradiance.

Nevertheless, our analyses of metabolites in a LL–ML transition do indicate that the initial rise of *A_n_* is restricted by slow activation of Rubisco and PPDK (for details, see Supplementary Text Section S3.2). They also reveal slow adjustment of *g_s_* (Supplementary Fig. S1H), leading to a minimum of *C_i_* at ∼60 s (Supplementary Fig. S1I). A similar minimum was seen after ∼30 s in fluctuating light (Sales et al. 2025). *Ci* fell to ∼80 ppm, which may slightly restrict photosynthesis in maize (Carmo-Silva et al. 2008; Bellasio et al. 2016; Sales et al. 2025). Part of the subsequent slow gain of *A_n_* may be related to gradual stomatal opening.

### Changing contribution of different decarboxylation routes and the maintenance of nitrogen stoichiometry

In addition to NADP-ME, the maize BSC contains PEPCK (Walker et al. 1997; Supplementary Text Section S4). Analyses of metabolite levels in high and low irradiance (Usuda 1987; Leegood and von Caemmerer 1989) and after a switch from high to low irradiance (Doncaster et al. 1989) as well as ^13^CO_2_ labeling kinetics in steady-state low and high irradiance (Medeiros et al. 2022) point to the aspartate-based PEPCK route making a larger contribution in low than high irradiance. The contribution may also be higher in leaves that have been previously subjected to shading (Arp et al. 2021).

Our study reveals that the contribution of aspartate-based decarboxylation changes during light transitions. Aspartate showed a strikingly different pattern to most other metabolites (Fig. 5; Supplementary Fig. S6), rising during the ML–LL transition (Fig. 3A; Supplementary Fig. S3B) and decreasing during the LL–ML transition (Fig. 4A; Supplementary Fig. S4B). This occurred from ∼120 s onward, pointing to a gradual increase in the contribution of PEPCK during adjustment to lower light and a gradual decrease during adjustment to higher light (see also Supplementary Test Section S4).

Movement of aspartate from the MC to the BSC needs to be coupled to movement of an amino acid back to the MC. In the most parsimonious pathway, this would be alanine (Weissmann et al. 2016; Bräutigam et al. 2018). However, in both transitions, aspartate and alanine changed independently and were often negatively correlated (Figs. 3A, 4A, and 5A; Supplementary Fig. S6, A and B; for details see Supplementary Test Section S4). This argues against operation of a tight shuttle between aspartate and alanine, which would require parallel changes of the 2 shuttle partners. Alternatively, nitrogen stoichiometry could be maintained by a secondary intercellular shuttle, for example, between glutamate and 2OG. The role of 2OG and glutamate in C_4_ photosynthesis has been previously discussed, especially in relation to increased flexibility of metabolic transformations and subcellular transport in the BSC (see, e.g. Hatch and Mau 1973; Chapman and Hatch 1981; Weissmann et al. 2016). This might contribute during adjustment to changed irradiance. In addition, our study provides correlative evidence that some amino groups from aspartate move back to the MC via a 2OG/glutamate shuttle. This includes the positive relation between aspartate and glutamate and negative relation between aspartate and the 2OG/glutamate ratio, both in the ML–LL transition (Fig. 3, A and B; Supplementary Fig. S3A) and the LL–ML transition (Fig. 4, A and B; Supplementary Fig. S4A) (see Supplementary Text Section S4 for details). Qualitatively similar changes in aspartate, alanine, glutamate, and 2OG were observed under 300 s fluctuating light (Sales et al. 2025). As suggested by Weissmann et al. (2016), secondary shuttles involving glutamate and 2OG would add flexibility to C_4_ photosynthesis. Interestingly, one of the metabolic traits that changed during evolution of C_4_ photosynthesis in the *Flaveria* genus was an increase in the levels of 2OG and glutamate (Borghi et al. 2022; Tang et al. 2024; see Supplementary Text Section S4).

The increased contribution of PEPCK in low irradiance may reflect multiple factors. On the one hand, any shortfall of NADPH in the MC would restrict conversion of OAA to malate, both due to mass action and because low NADPH/NADP ratios inhibit thioredoxin activation of NADP-MDH (Ashton and Hatch 1983; Rebeille and Hatch 1986; Ashton et al. 1990). Providing amino groups are available, this would channel more OAA to aspartate. In the BSC, NADP-ME activity may be restricted by lack of demand for the reaction products (Bräutigam et al. 2018) and incomplete posttranslational activation (Bovdilova et al. 2019). On the other hand, maize PEPCK is not subject to posttranslational regulation (Wingler et al. 1999; Walker et al. 2002) and is presumably as active in low as in high irradiance. Furthermore, operation of PEPCK requires movement of PEP from the BSC back to the MC. In principle, this could occur as diffusion of PEP or, alternatively, as diffusion of PGA with enolase and phosphoglycerate mutase converting PEP to 3PGA in the BSC and 3PGA to PEP in the MC. PEP levels and/or enolase and phosphoglycerate mutase activity may suffice to support the low rate of movement required in LL but become restrictive under high light (see also Supplementary Text Section S4 and Sales et al. 2025).

### Metabolite analysis point to rapid perturbation and slow adjustment of the CO_2_ concentration in the bundle sheath

Changes in irradiance may lead to a temporary imbalance between the concentration of CO_2_ by the CCM and its utilization by Rubisco (Furbank et al. 1990; von Caemmerer 2000; Kromdijk et al. 2014; Wang et al. 2022). An excess of CO_2_ consumption over CO_2_ influx will lead to a decrease in C_BSC_ and increased RuBP oxygenation relative to carboxylation, while an excess of CO_2_ influx over CO_2_ consumption will lead to an increase in C_BSC_ and increased back-leakage of CO_2_. However, measurement of C_BSC_ is technically challenging, especially in transients. Measurements of 2PG provide a qualitative proxy for the rate of RuBP oxygenation and, hence, C_BSC_ (for details, see Supplementary Text Section S4).

Immediately following a decrease in irradiance, there was a significant >2-fold decrease in the level of 2PG (Fig. 3A; Supplementary Fig. S3B), a decline of the RuBP/3PGA ratio, and an increase of the RuBP/2PG ratio, consistent with a transient increase in carboxylation relative to oxygenation. Conversely, following an increase in irradiance, there was a significant increase of 2PG (Fig. 4A; Supplementary Fig. S8A), an increase of the RuBP/3PGA ratio, and a decrease of the RuBP/2PG ratio (Fig. 4B; Supplementary Fig. S8B), consistent with a transient decrease in carboxylation relative to oxygenation. Similar changes of 2PG were found in a 300 s fluctuating light regime (Sales et al. 2025). These observations point to C_BSC_ increasing after a decrease in light intensity, and decreasing after an increase in irradiance, with accompanying changes in back-leakage of CO_2_.This could contribute to the changes in the relationship between the estimated rate of electron transport and *A_n_* observed in fluctuating light regimes.

Finally, photorespiration contributes to transients of *A_n_* in changing light in C_3_ species, due both to delayed release of CO_2_ by glycine decarboxylase and because photorespiratory intermediates sequester and act as a source of C for the CBC (Fu et al. 2023). Glycine fell in the ML–LL transition and rose in the LL–ML transition, but the magnitude of the changes was too small to make more than a minor contribution to the *A_n_* or the dynamics of CBC and CCM metabolite pools, and other photorespiratory intermediates did not show consistent changes (Supplementary Text Section S6).

In conclusion, several subprocesses in metabolism modify the photosynthetic efficiency of maize during sudden transitions in light intensity. After a decrease in irradiance, photosynthesis is transiently buffered by energy stored in large pools of metabolites that are involved in intercellular shuttles. However, photosynthesis subsequently declines to a trough, and it takes several minutes to recover to the steady rate in LL. One reason for the trough and slow recovery is that the large pools of metabolites involved in intercellular shuttles run down too far, and it takes time to build them up again. After an increase in irradiance, it takes several minutes to establish a steady and higher rate of photosynthesis. While the first part of this delay is associated with building-up pools of CBC metabolites and, possibly, activation of enzymes, the second part is mainly due to the need to build-up the large pools of metabolites required to drive the intercellular shuttles. In addition, transient imbalances between the concentration and utilization of CO_2_ can lead to higher photorespiration or back-leakage of CO_2_ after an increase and decrease in irradiance, respectively. Under fluctuating light, the impact of these metabolic responses will depend, among other things, on the frequency of the fluctuations. When rapid fluctuations are experienced, as may often be the case in a canopy in the field, the gains immediately after the drop in irradiance may outweigh losses after longer exposure to LL. Photosynthetic efficiency in fluctuating regimes might be improved by enhancing the ability to rapidly regulate end-product synthesis and maintain higher metabolite levels at low irradiance, both to avoid a trough of *A_n_* after a decrease in irradiance and to allow faster recovery of *A_n_* when irradiance increases.

## Materials and methods

### Chemicals

Chemicals were from Sigma-Aldrich (Darmstadt, Germany; www.sigmaaldrich.com), Roche Applied Science (Mannheim, Germany; lifescience.roche.com), or Merck (Darmstadt, Germany; www.merckmillipore.com).

### Plant growth

For gas exchange parameters recorded every second, maize (*Zea mays* L. cv. B73) seeds were sown in Levington advance F2 compost (Scotts, Ipswich, UK) in seed trays and germinated under 14/10 h day/night cycles (irradiance 550 *µ*mol photons m^−2^ s^−1^, 28/20 °C, 65% relative humidity). After 1 wk, seedlings were transplanted to 1.3 L pots, containing a mixture of 2:2:1 of Levington M3 compost (Scotts, Ipswich, UK): top soil (Westland, Dungannon, UK): perlite 2.0 to 5.0 mm (Sinclair, Ellesmere Port, UK). Each pot was supplemented with 3.5 g L^−1^ of magnesium salts (Scotts Miracle-Gro, Marysville, OH, USA) and 7 g L^−1^ of garden lime (Needham Chalks, Suffolk, UK) and moved to a cabinet set to temperature conditions of 29 °C/22 °C light/night. After a week, a 5 g osmocote fertilizer tablet (14N-8P:11K + 2MgO + trace elements; Osmocote Exact, ICL, Saltburn, UK) was added per pot.

For metabolic sampling and gas exchange parameters recorded at 6 s intervals, maize (*Z. mays* L. cv. B73) seeds were germinated in darkness in petri dishes on moistened filter paper (3 d, 28 °C), transferred to soil (Einheitserde Typ Topf [Naturton, white peat, wood fibers] mixed 2:1 with quartz sand; www.einheitserde.de) in 10 cm diameter pots, and grown for 5 d under 16/8 h day/night cycles (irradiance 105 *µ*mol photons m^−2^ s^−1^, 22/18 °C, 70% relative humidity) and then under 14/10 h day/night cycles (irradiance 550 *µ*mol photons m^−2^ s^−1^, 29/22 °C, 65% relative humidity).

For both growth conditions, plants were well watered. The fourth fully elongated leaves of 3-wk plants were used.

### Gas exchange

For gas exchange parameters recorded every second, plants were transferred to a Percival E-41HO controlled environment chamber (Perry, IA, USA) set at 22 °C on the evening before measurements. In the morning, lights were turned on, and irradiance inside the chamber was kept at 550 *μ*mol m^−2^ s^−1^ (ML) or 160 *μ*mol m^−2^ s^−1^ (LL), to perform the ML–LL or LLML transition experiments, respectively. Each irradiance transition was performed in consecutive days. Gas exchange parameters were measured using a Li-6800 portable infrared gas analyzer (IRGA) system (software version 1.4.22, LI-COR, Lincoln, Ne, USA) with a 6 cm^2^ chamber. Air CO_2_ concentration in the reference analyzer was controlled at 400 *μ*mol mol^−1^, heat exchanger temperature at 25 °C, and relative humidity at 65%, and the relative proportion of blue light was set to 10% photosynthetically active photon flux density (PPFD). Flow rate was set to 600 *µ*mol s^−1^. Gas exchange parameters were logged every second. Measurements started about 4 h into the light period. For the ML–LL transition, leaves were measured for 15 min at 550 *μ*mol m^−2^ s^−1^, then light inside the chamber was switched to 160 *μ*mol m^−2^ s^−1^, and gas exchange parameters were logged for another 30 min. For the LL–ML transition, leaves were measured for 15 min at 160 *μ*mol m^−2^ s^−1^, then light inside the chamber was switched to 550 *μ*mol m^2^ s^1^, and parameters were logged for another 30 min. As gas exchange measurements during and shortly after the light switch strongly violate the steady-state assumption underlying default rate equations, dynamic equations were implemented (Saathoff and Welles 2021) to correct this measurement series. The dynamics of water vapor fluxes are less accurately measured than CO_2,_ mainly due to absorption/desorption on cuvette walls, which dampen the responses and their derivatives (i.e. g_s_ and Ci).

Gas exchange parameters recorded at 6 s intervals were performed with plants in the growth chamber without shade (550 *µ*mol m^−2^ s^−1^, ML) or with plants under a large polystyrene plate to reduce incident irradiance (160 *µ*mol m^−2^ s^−1^, LL). Measurements were performed on 2 d for ML–LL and 1 d for LL–ML. Gas exchange parameters were measured using an open-flow infrared gas exchange analyzer system (LI-6400XT, LI-COR Inc. Lincoln, NE, USA) equipped with an integrated fluorescence chamber head (LI-6400-40, 2 cm^2^ leaf chamber; LI-COR Inc. Lincoln, NE). The PPFD inside the chamber was kept at 550 *µ*mol m^−2^ s^−1^ (ML) or 160 *µ*mol m^−2^ s^−1^ (LL). Blue light was set to 10% PPFD, and CO_2_ was kept at 400 *µ*mol mol^−1^, leaf temperature at 29 °C and relative humidity at 65%. Gas exchange parameters were recorded at 6 s intervals. This data series could not be corrected using dynamic equations for technical reasons (each five 6 s time point was a recorded running average; see Supplementary Text Section S1). Measurements started about 4 h into the light period. In the ML–LL and LL–ML transitions, leaves were measured for 15 min at the initial irradiance, then light inside the chamber was switched, and parameters were logged for 45 and 55 min in the ML–LL and LL–ML transitions, respectively. In a separate experiment with the same batch of plants, a light saturation curve was performed over a range of irradiance up to 2,000 *µ*mol m^−2^ s^−1^, with preincubation at 1,000 *µ*mol m^−2^ s^−1^, before transfer to 2,000 *µ*mol m^−2^ s^−1^, and measurement at successively lower irradiances for at least 3 min.

### Sampling for metabolite measurements

Light switch experiments were performed with separately grown sets of plants for the ML to LL and the LL to ML using growth irradiance (550 *µ*mol photons m^−2^ s^−1^, ML) and low irradiance (160 *µ*mol photons m^−2^ s^−1^, LL, obtained by placing a polystyrene plate below the light source). For the LL to ML switch, maize plants were grown at 550 *µ*mol photons m^−2^ s^−1^ and then shaded with a polystyrene plate, starting just before dusk on the day prior to the experiment. LL to ML and ML to LL switches were performed by moving maize plants individually from 1 light condition to the other light condition and harvesting 5, 10, 15, 30, 60, 120, 300, 600, 1,200, or 1,800 s after transfer. Control samples (*t* = 0 s; plant not transferred) were also harvested (*n* = 4 and 10 for ML to LL and LL to ML switch, respectively). The ML–LL and LL–ML treatments were performed at a 2-wk interval on separately grown batches of plants. Samples for a given light switch treatment were collected on a single day. On each day, the first light switch treatment started at about 4 h after light on, and the last was timed such that the treatment and harvest was completed by 6.5 h after light on. This narrow time window was used to minimize sample-to-sample variation due to gradual accumulation of metabolites during the light period. Samples for different time points were randomized across time of day. The material was harvested by cutting the leaf (7 cm long section, about 10 cm below the leaf tip) and quenching it in a bath of liquid N_2_ under prevalent irradiance, avoiding shading. After quenching, the frozen plant material was stored at −80 °C until further use.

A second ML–LL and LL–ML light switch experiment was performed with separately grown plants, with less time points (0, 10 s, and 20 min, to capture changes immediately after the change in irradiance and at the end of the transition), but more replicates (*n* = 10), and samples for 1 light switch being collected on 1 d and the other light switch on the following day.

### Metabolite analyses

Frozen samples were homogenized using a ball mill (Retsch, Haan, Germany; https://www.retsch.com) at liquid N_2_ temperature. Metabolites were extracted and quantified by LC–MS/MS and GC–MS as described in Arrivault et al. (2019) and in Lisec et al. (2006), respectively. PEP, pyruvate, and 3PGA were determined enzymatically in freshly prepared trichloroacetic acid extracts (Merlo et al. 1993) using a spectrophotometer (Shimadzu, Kyoto, Japan; www.shimadzu.de).

### Statistical analyses

Statistical analysis was performed in Excel and in R Studio version 1.1.463 (www.rstudio.com) with R version 3.5.1 (https://cran.r-project.org/). Additional information is in he figure legends.

## Supplementary Material

kiaf508_Supplementary_Data

## Data Availability

All data obtained for this study are presented within the Supplementary Data Sets.

## Supplementary data

The following materials are available in the online version of this article.

**Supplementary Figure S1.** Additional gas exchange data.

**Supplementary Figure S2.** PC analysis: loadings of metabolites.

**Supplementary Figure S3.** Further metabolites and statistical tests in the ML–LL transition or the LL–ML transition.

**Supplementary Figure S4.** Further metabolites and statistical tests in the LL–ML transition.

**Supplementary Figure S5.** Changes of individual metabolites, metabolite ratios, and sets of metabolites in additional light transitions.

**Supplementary Figure S6.** Relationship between *A_n_* and metabolite levels.

**Supplementary Figure S7.** Additional plots of metabolite levels and metabolic traits against *A_n_* in a transition from ML to LL.

**Supplementary Figure S8.** Additional plots of metabolite levels and metabolic traits against *A_n_* in a transition from LL to ML.

**Supplementary Text.**

**Supplementary Data Set 1.** Gas exchange data.

**Supplementary Data Set 2.** Metabolite data in the time-resolved ML–LL and LL–ML transitions.

**Supplementary Data Set 3.** Metabolite data at 10 and 1,200 s in an independent ML–LL and LL to ML transition.

## Dive Curated Terms

The following phenotypic, genotypic, and functional terms are of significance to the work described in this paper:

ICL Gramene: AT3G21720
ICL Araport: AT3G21720

NADPH CHEBI: CHEBI:16474

ATP CHEBI: CHEBI:15422

aspartate CHEBI: CHEBI:29995

PPDK Gramene: AT4G15530
PPDK Araport: AT4G15530

PEPCK Gramene: AT4G37870
PEPCK Araport: AT4G37870
