# Supplementary material for: Metabolite profiling reveals slow and uncoordinated adjustment of C4 photosynthesis to sudden changes in irradiance
Source: Plant Physiol. 2025 Oct 13;199(3):kiaf508. doi: 10.1093/plphys/kiaf508 (PMC12624396; doi:10.1093/plphys/kiaf508)
Supplement: kiaf508_Supplementary_Data [file kiaf508_supplementary_data.zip › Supplemental material.pdf]

Metabolite profiling reveals slow and uncoordinated adjustment of C_4 photosynthesis to sudden changes in irradiance, Stéphanie Arrivault, David Barbosa Medeiros, Cristina Rodrigues Gabriel Sales, Manuela Guenther, Johannes Kromdijk, Alisdair R. Fernie, and Mark Stitt

Supplementary Material

Contents **page**

Contents	page
**Supplementary Figure S1**	**2**
**Supplementary Figure S2**	**6**
**Supplementary Figure S3**	**8**
**Supplementary Figure S4**	**9**
**Supplementary Figure S5**	**10**
**Supplementary Figure S6**	**14**
**Supplementary Figure S7**	**17**
**Supplementary Figure S8**	**19**

Supplementary Text

1. Gas exchange of plants sampled for metabolite measurements **21**
Supplementary to Results sections ‘*Response of CO_2 assimilation and stomatal conductance; Global analysis of response of metabolism’*

2. Rate of exchange of C between CBC (incl. energy shuttle metabolites) and CCM **21**
2.1. Background
2.2. Implications for movement of C between CBC and CCM pools in the ML-LL transition. Supplementary to the Discussion section ‘*Response of the CBC and CCM to a decrease in irradiance*
2.3. Implications for movement of C between CBC and CCM pools in the LL-ML transition. Supplementary to the Discussion section ‘*Response of the CBC and CCM to an increase in irradiance’*

3. Detailed account of changes in metabolism during ML-LL and LL-ML transitions **25**
3.1. Supplementary to the Discussion section ‘*Response of the CBC and CCM to a sudden decrease in irradiance*
3.2. Supplementary to the Discussion section ‘*Response of the CBC and CCM to an increase in irradiance’*

4. Changes in the contribution of different decarboxylation routes **34**
Supplementary to the Discussion section ‘*Contribution of different decarboxylation routes’*

5. Perturbation and adjustment of C_{BSC} **39**
Supplementary to Discussion section ‘*Metabolite analysis point to rapid perturbation and slow adjustment of the CO_2 concentration in the bundle sheath’*

6. Sequestration or recycling of C from pools in the photorespiratory pathway **41**
Supplementary to section ‘*Metabolite analysis point to rapid perturbation and slow adjustment of the CO_2 concentration in the bundle sheath*

Additional references **42**

Supplementary Datasets S1, S2 and S3 are available as separate files

Supplementary Figure S1. Additional gas exchange data.

These figures are Supplementary to Fig. 1, the data are provided in Supplementary Dataset S1.

(**A**) Light saturation response (mean ± SD, *n*=5 leaves on separate plants).

(**B**) Time linear plot of A_n for the ML-LL transition (mean ± SD, *n*=5).

(**C**) Curve fitting of the decrease of A_n during the ML-LL transition. The plot shows mean values (*n*=5) and the curve fitting is blue.

(**D**) Time linear and log scaled plots of g_s for the ML-LL transition (mean ± SD, *n*=5). The time point in white corresponds to time 0, before the light transition.

(**E**) Time linear and log scaled plots of *Ci* for the ML-LL transition (mean ± SD, *n*=5). The time point in white corresponds to time 0, before the light transition.

(**F**) Time linear plot of A_n for the LL-ML transition (mean ± SD, *n*=6). (

(**G**) Curve fittings of the increases of A_n during the LL-ML transition. The plots show mean values (*n*=6) and the curve fittings are blue.

(**H**) Time linear and log scaled plots of g_s for the LL-ML transition (mean ± SD, *n*=6). The time point in white corresponds to time 0, before the light transition.

(**I**) Time linear and log scaled plots of *Ci* for the LL-ML transition (mean ± SD, *n*= 6). The time point in white corresponds to time 0, before the light transition.

(**J**) Comparison of *An* data from Figure 1 corrected by dynamic equation ("corrected") and uncorrected ("non-corrected") for the M-LL and LL-ML transitions (mean ± SD, *n*=5). The time point in white corresponds to time 0, before the light transition. The plots are presented with time on a log scale.

(**K**) Comparison of two independent experiments obtained for the ML-LL transition and for the LL-ML transition; "non-corrected" are data from the experiment shown in Figure 1, where data was recorded every second but, in this plot, (like panel J) are not corrected by the dynamic equation (i.e., as in panel J), "non-corrected 2" are data from an independent experiment in which *An* was measured at 6 s intervals, and correction was not possible as each time point was a recorded data average. This second set of measurement was performed on the batch of plants that were sampled for metabolite analyses. For a better comparison between the experiments, data are shown as a percentage of *An* at time 0. The plots show ± SD (*n*=5 for non-corrected ML-LL, *n*=6 for non-corrected LL-ML, *n*=3 for non-corrected 2 ML-LL and *n*=5 for non-corrected 2 LL-ML). The time points in white correspond to time 0, before the light transition.

(**L**) Expansion of the ML-LL response from 60 s onwards to show the slight recovery of A_n in the independent uncorrected experiment (data recorded every 6 seconds, not corrected by dynamic equation). The plot shows mean ± SD (*n*=3). The relatively high SD is due to differences in A_n between the three independent replicates (see Supplemental Dataset S1B). Significance was analyzed using a paired t-test, to focus on the comparison between different time points, and separate this from differences in A_n between replicates. The increase was significant (p = 0.03, n = 3, paired t-test comparing for each replicate average A_n between 267-300 s with average A_n between 1776-1800 s). The x-axis corresponds to time on a linear scale.

(**M**) Each individual leaf measurement of corrected A_n shown separately for replicate LL-ML transition (Figure 1B shows the average and SD of these values). The plot is presented with time on a log scale.

Supplementary Figure S1. Continued.

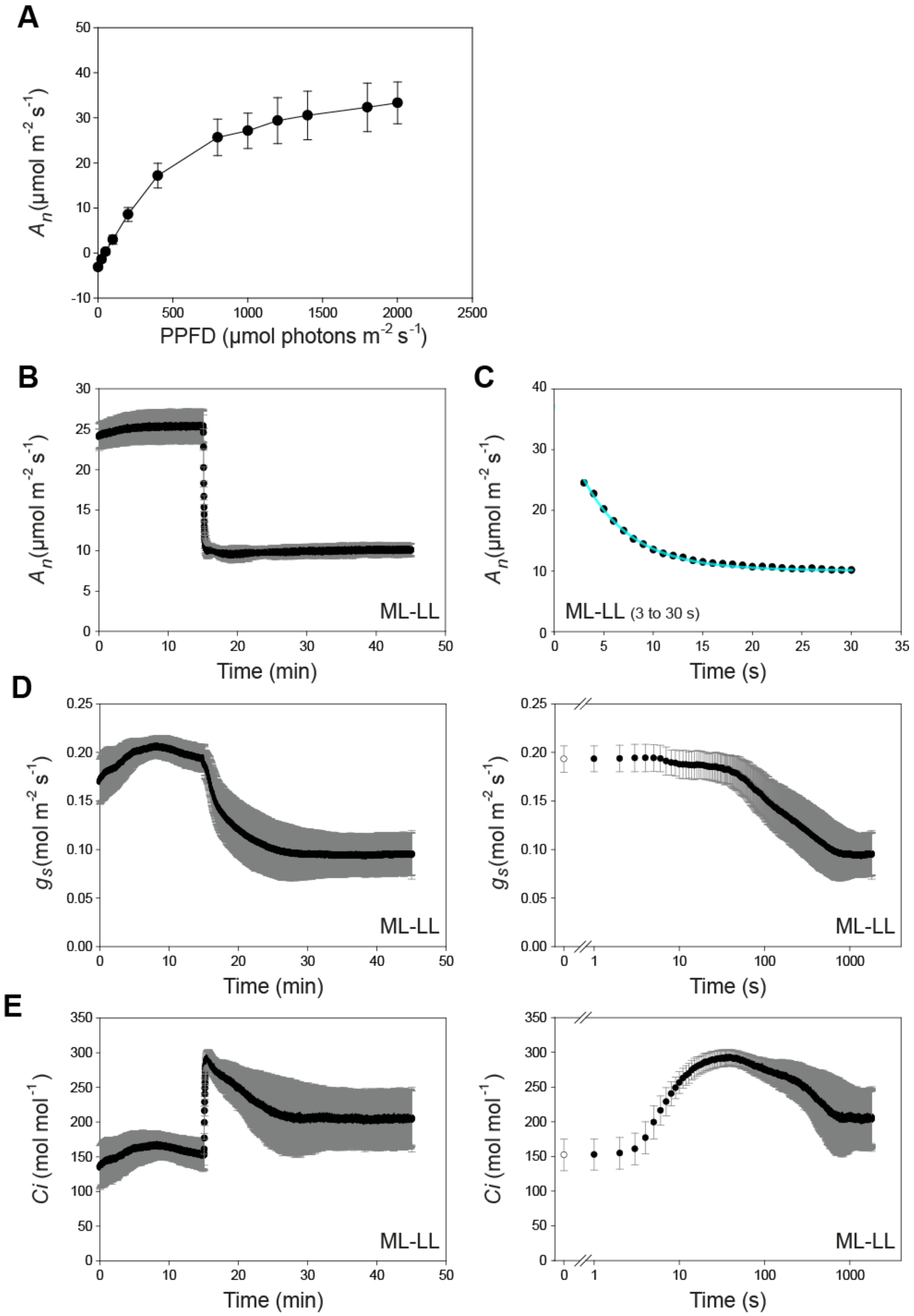

Supplementary Figure S1. Continued.

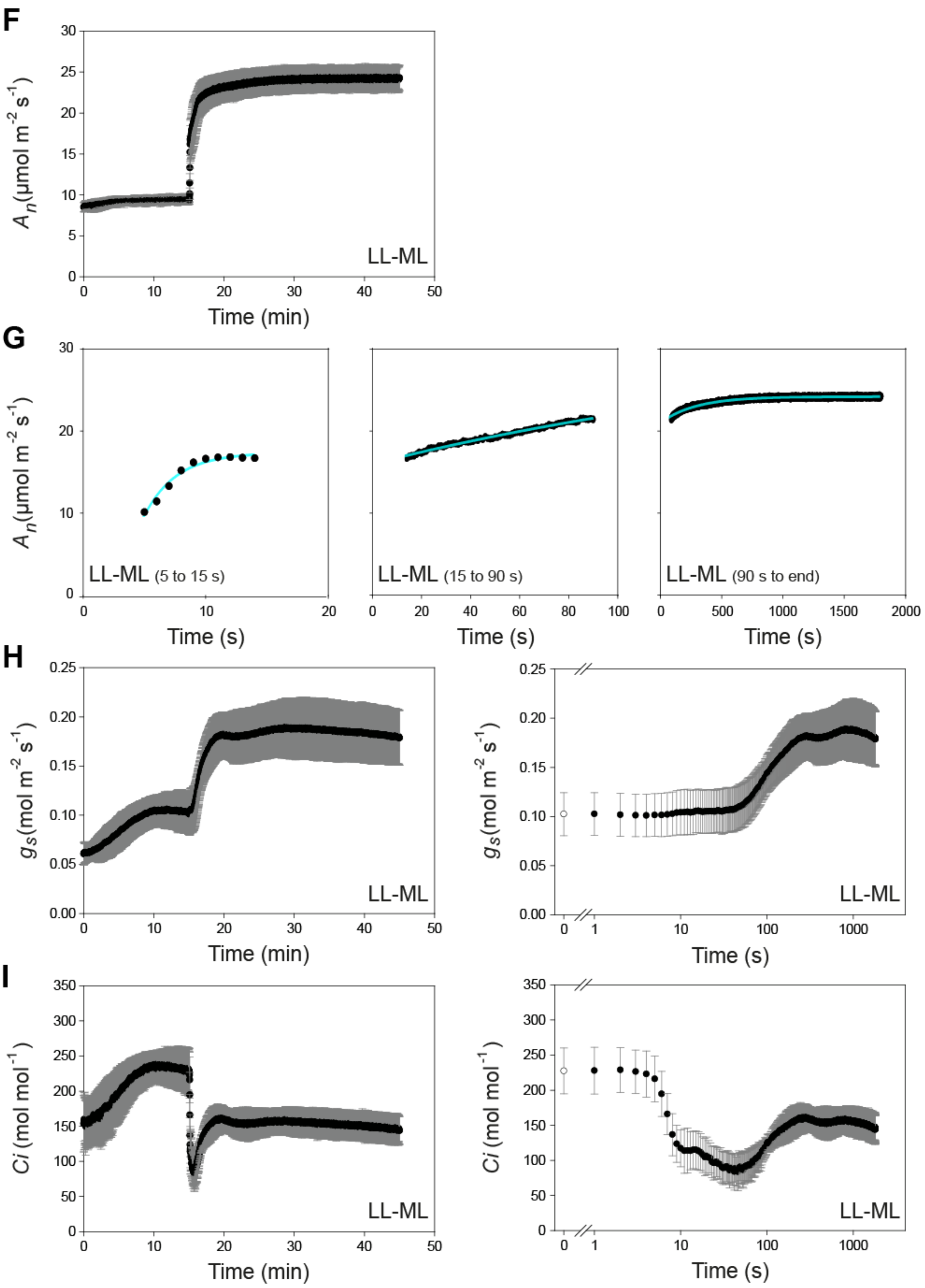

Supplementary Figure S1. Continued.

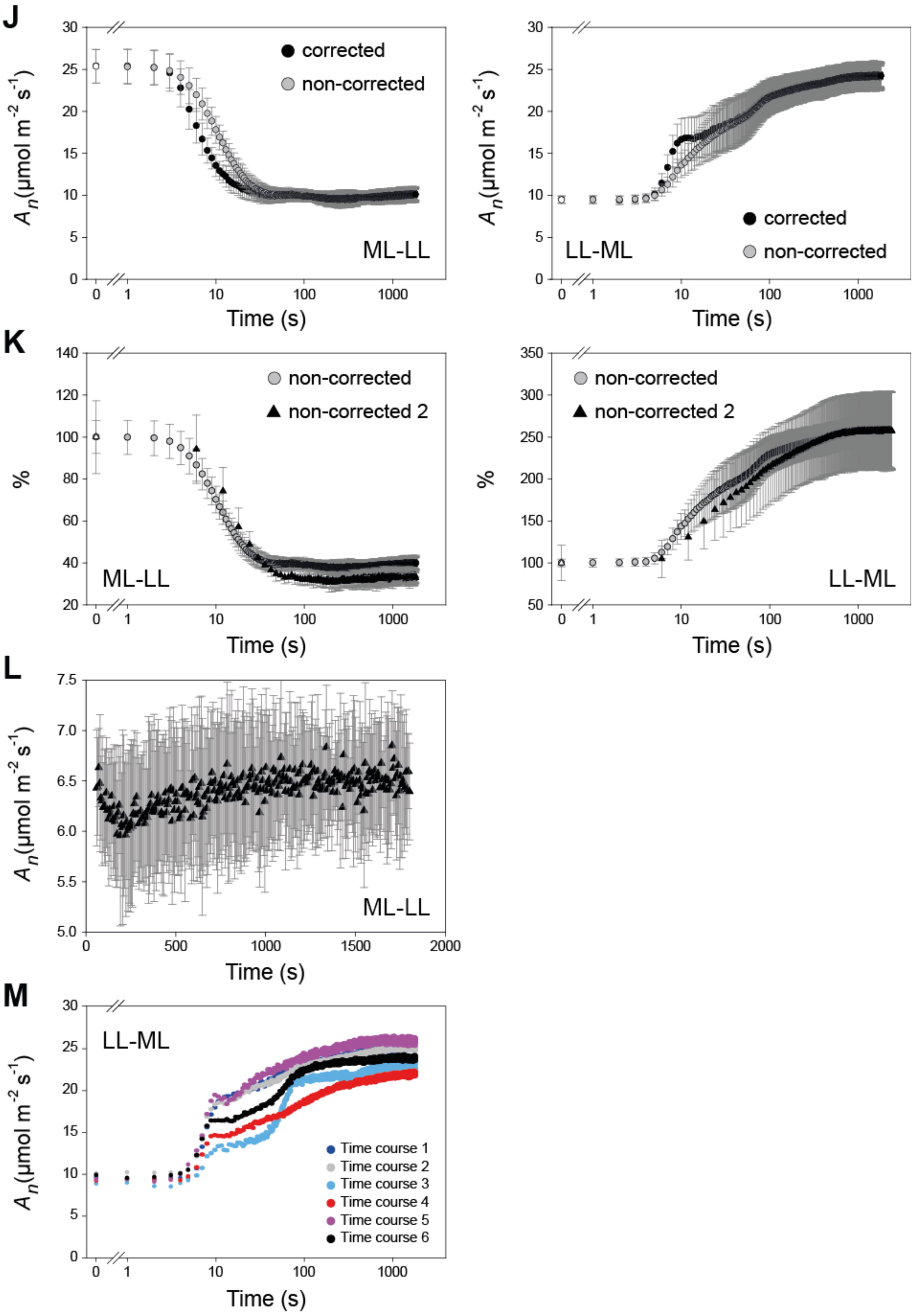

Supplementary Figure S2. Principal Components Analysis: loadings of metabolites.

(A) ML-LL transition

(B) LL-ML transition.

Means for each time point were used to perform the analyses (*n*=4 to 5 for ML-LL, and *n*=4, except for time 0 s where *n*=10 for LL-ML). In (A) and (B), both the PC analysis (as in Fig. 2) and the variables are included. Variables are depicted as small boxes, with CO_2 assimilation (An) and metabolites from the CBC and metabolites from the CCM highlighted in while, dark grey and light grey, respectively. The time points in the PCA are identified by color and symbol (see insert) and the arrows show the time sequence in the transition. This figure is supplementary to Fig. 2.

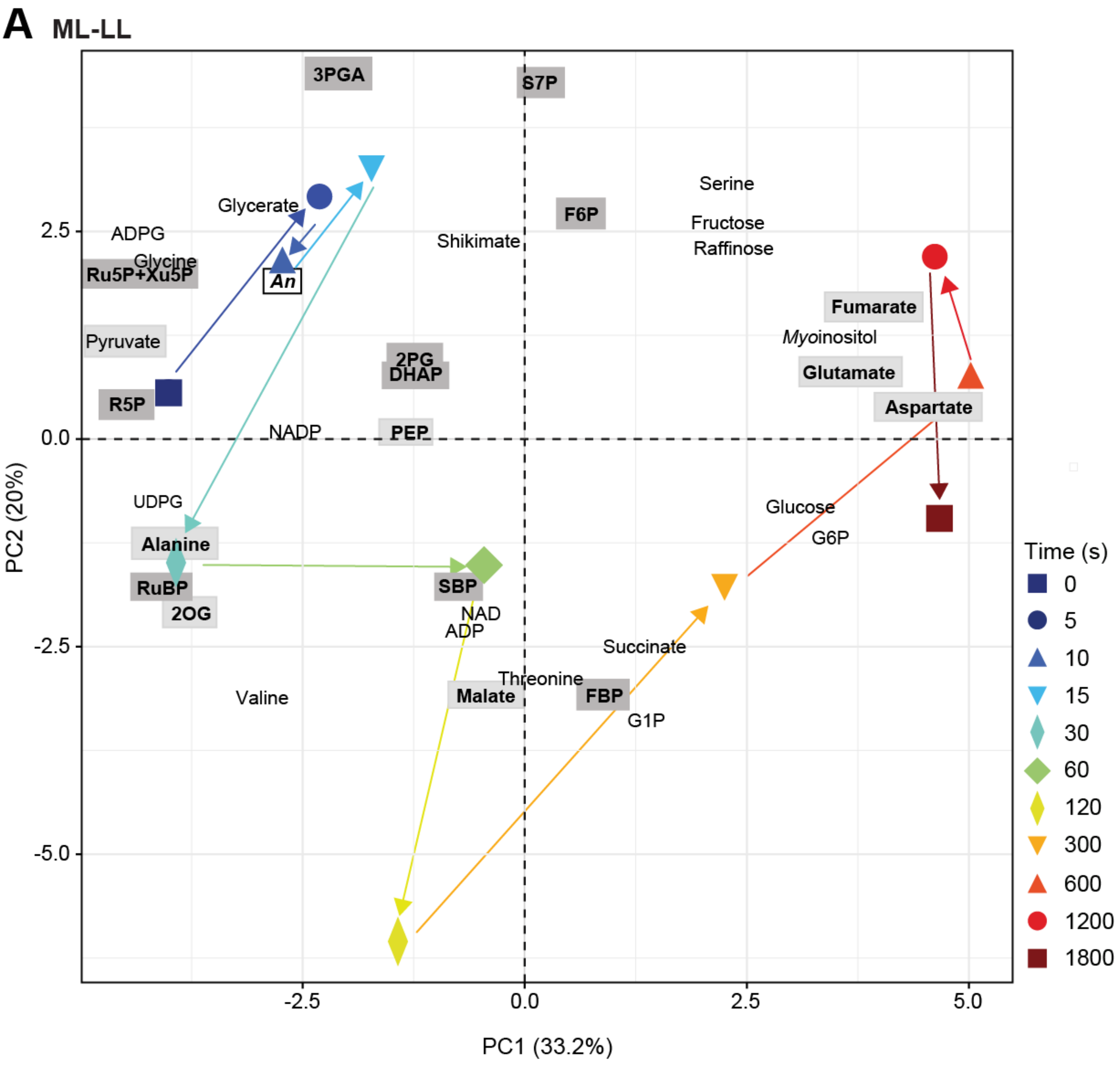

Supplementary Figure S2. Continued.

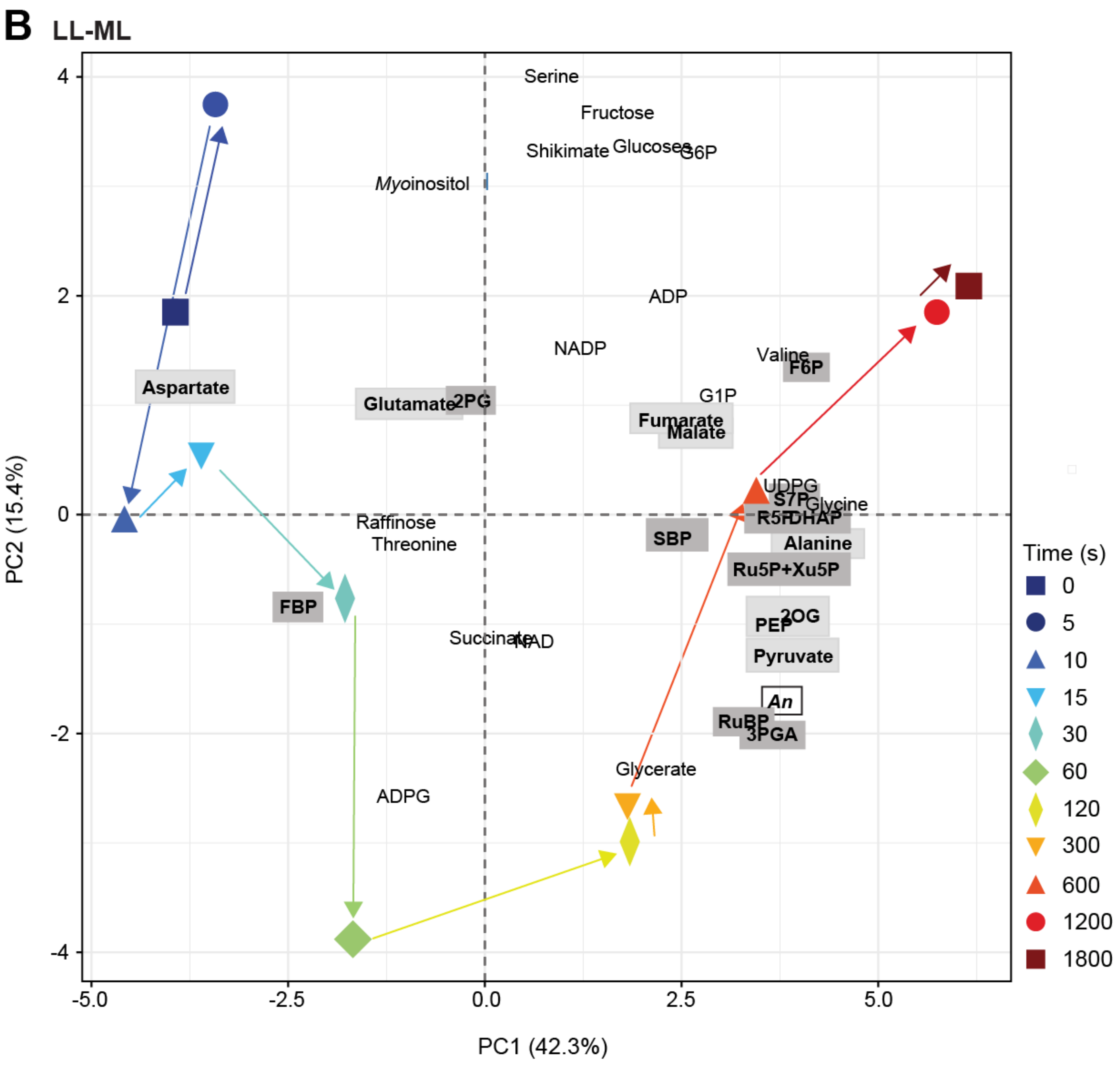

Supplementary Figure S3. Further metabolites and statistical tests in the moderate to low light transition, or the low to moderate light transition.

(**A**) Moderate light to low light transition. Amounts of metabolites (nmol g^{-1} FW) are shown as mean ± SD (*n*=4 to 5). Significant differences (T-test) to time zero (i.e., ML) are indicated by stars (* $p < 0.05$; ** $p < 0.01$: *** $p < 0.001$). Time is shown on a log scale. For original data, see Supplementary Dataset S2.

(**B**) Visualization of significant changes. Two approaches were taken. In one approach (left hand side) the entire time sequence was analyzed. T-tests were performed individually between time zero (i.e., ML) and each time in the time sequence. In the second approach (right hand side) the response was divided into time segments to test for changes in different phases of the transient that might be masked in the complete transient. Time segments were defined based on the PCA of the combined CO2 assimilation response and metabolome response (Fig. 2). Time segments are separated by solid vertical lines, and the grey column indicates first time in a given time segment. T-tests were performed between the first time in the time segment and each later time in the time segment. The results are visualized as stars (* $p < 0.05$; ** $p < 0.01$: *** $p < 0.001$; blue denotes an increase and red a decrease (see legend). Time segments are also indicated by colored blocks in **A**.

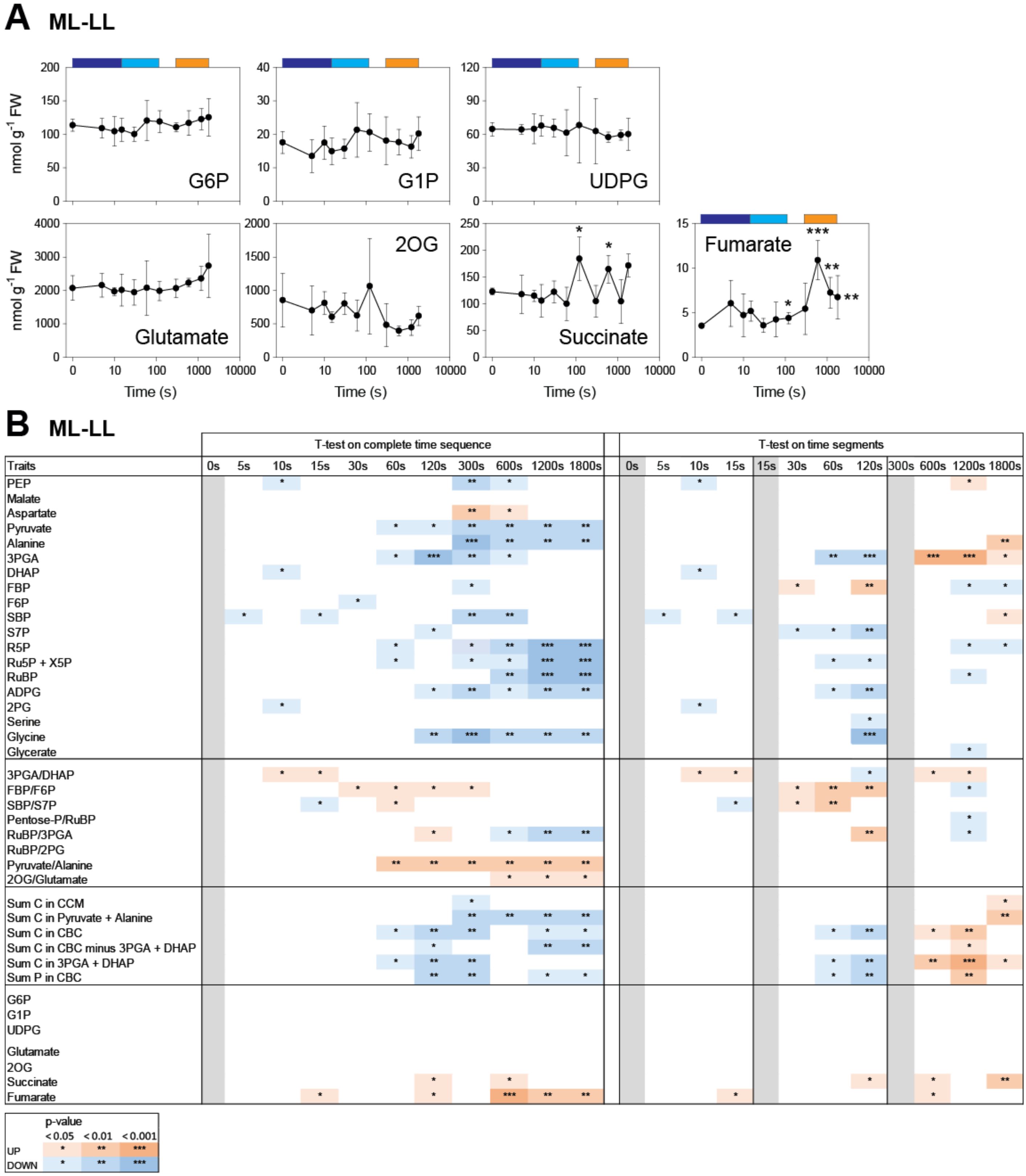

Supplementary Figure S4. Further metabolites and statistical tests in the low to moderate light transition.

(**A**) Low light to moderate light transition. Amounts of metabolites (nmol g^{-1} FW) are shown as mean ± SD (*n*=4 except for time zero where *n*=10). Significant differences (T-test) to time zero (i.e., LL) are indicated by stars (* p < 0.05; ** p < 0.01: *** p < 0.001). Time is shown on a log scale. For original data, see Supplementary Dataset S2.

(**B**) Visualization of significant changes. Two approaches were taken. In one approach (left hand side) the entire time sequence was analyzed. T-tests were performed individually between time zero (i.e., ML in the ML-LL transition and LL in the LL-ML transition) and each time in the time sequence. In the second approach (right hand side) the response was divided into time segments to test for changes in different phases of the transient that might be masked in the complete transient. Time segments were defined based on the PCA of the combined CO_2 assimilation response and metabolome response (Fig. 2). Time segments are separated by solid vertical lines, and the grey column indicates first time in a given time segment. T-tests were performed between the first time in the time segment and each later time in the time segment. The results are visualized as stars (* p < 0.05; ** p < 0.01: *** p < 0.001; blue denotes an increase, red a decrease (see legend). Time segments are also indicated by colored blocks in **A**.

A LL-ML

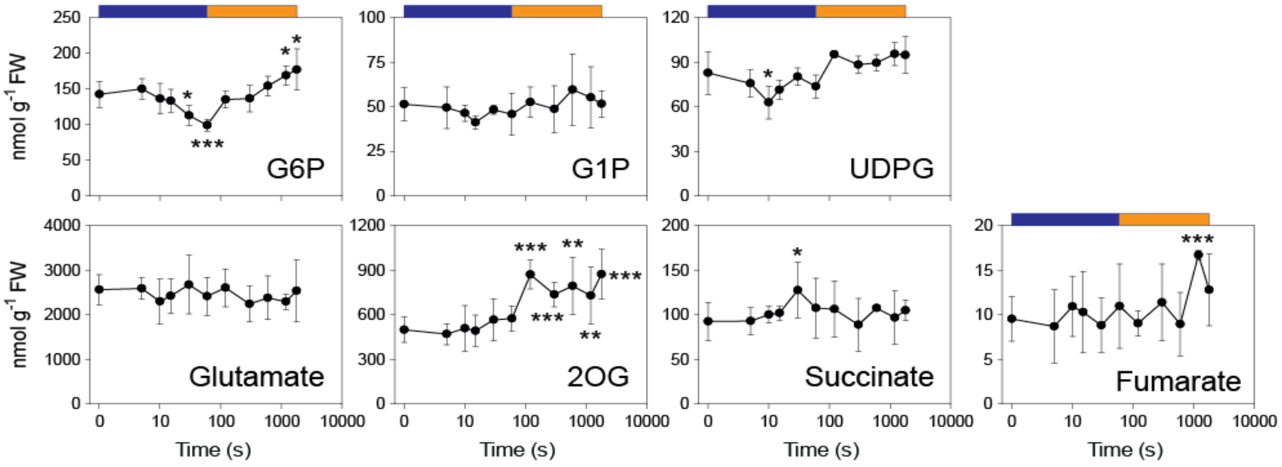

B LL-ML

Traits	T-test on complete time sequence												T-test on time segment					
	0s	5s	10s	15s	30s	60s	120s	300s	600s	1200s	1800s		60s	120s	300s	600s	1200s	1800s
PEP		*** (DOWN)	** (DOWN)	* (DOWN)				*		*	**							
Malate																		
Aspartate							* (DOWN)	* (DOWN)	** (DOWN)	* (DOWN)	* (DOWN)							
Pyruvate		*		*	**	***	***	***	***	***	***			*	***			**
Alanine							*	**	**	**	**							
3PGA							*	*		*								
DHAP		*			**	***	***	***	***	***	***			*			**	*
FBP					*			* (DOWN)	* (DOWN)						** (DOWN)	** (DOWN)	** (DOWN)	** (DOWN)
F6P							**	*	**	***	***			*		*	**	*
SBP																		
S7P				*	**	*	***	*	*	***	***							
R5P							*		*	*	*							
Ru5P + X5P		*	*		**	*	***	***	***	***	***							
RuBP					*	*	*		*	**	*							
ADPG			**	***	**	**	**	***	***	***	***							
2PG		*					**							**				
Serine						* (DOWN)											*	
Glycine							**	***	***	**	***				*		*	*
Glycerate								*										
3PGA/DHAP		* (DOWN)					* (DOWN)			* (DOWN)	* (DOWN)						* (DOWN)	
FBP/F6P		** (DOWN)	** (DOWN)		*	**	** (DOWN)	*** (DOWN)	*** (DOWN)	*** (DOWN)	*** (DOWN)			** (DOWN)	*** (DOWN)	*** (DOWN)	*** (DOWN)	*** (DOWN)
SBP/S7P																		
Pentose-P/RuBP		*																
RuBP/3PGA									*									
RuBP/2PG						*								* (DOWN)				
Pyruvate/Alanine		**	**	*	*	***	***	***	**	**	***							
2OG/Glutamate							***	***	*	**	***			*	**			*
Sum C in CCM											*							
Sum C in Pyruvate + Alanine							**	***	**	***	***						*	*
Sum C in CBC					**	***	***	***	***	***	***						*	
Sum C in CBC minus 3PGA + DHAP					**	**	***	*	***	***	***							
Sum C in 3PGA + DHAP					*	**	***	***	**	***	***							
Sum P in CBC					**	***	***	***	***	***	***						*	
G6P					* (DOWN)	*** (DOWN)				*	*			**	**	***	***	**
G1P																		
UDPG			* (DOWN)											**	*	*	**	*
Glutamate																		
2OG							***	***	**	**	***			**	*			*
Succinate					*													
Fumarate										***								

p-value	< 0.05	< 0.01	< 0.001
UP	*	**	***
DOWN	*	**	***

Supplementary Figure S5. Changes of individual metabolites, metabolite ratios and sets of metabolites in additional light transitions.

(A-B) Levels of metabolites at 10 s and 1200 s

(A) ML-LL transition,
(**B**) LL-ML transition (plot overleaf)

Data shown are means ± SD (n=10 for ML-LL, n= 9 to 10 for LL-ML). Statistically significant differences from time zero were determined by t-test (* p < 0.05; ** p < 0.01: *** p < 0.001). All x-axes correspond to time on a log scale. The original data are provided in Supplementary Dataset S3.

(C-D) Comparison of changes of individual metabolites, metabolite ratios and sets of metabolites at 10 s and 1200 s in the two experiments (plots overleaf, two and three pages later)

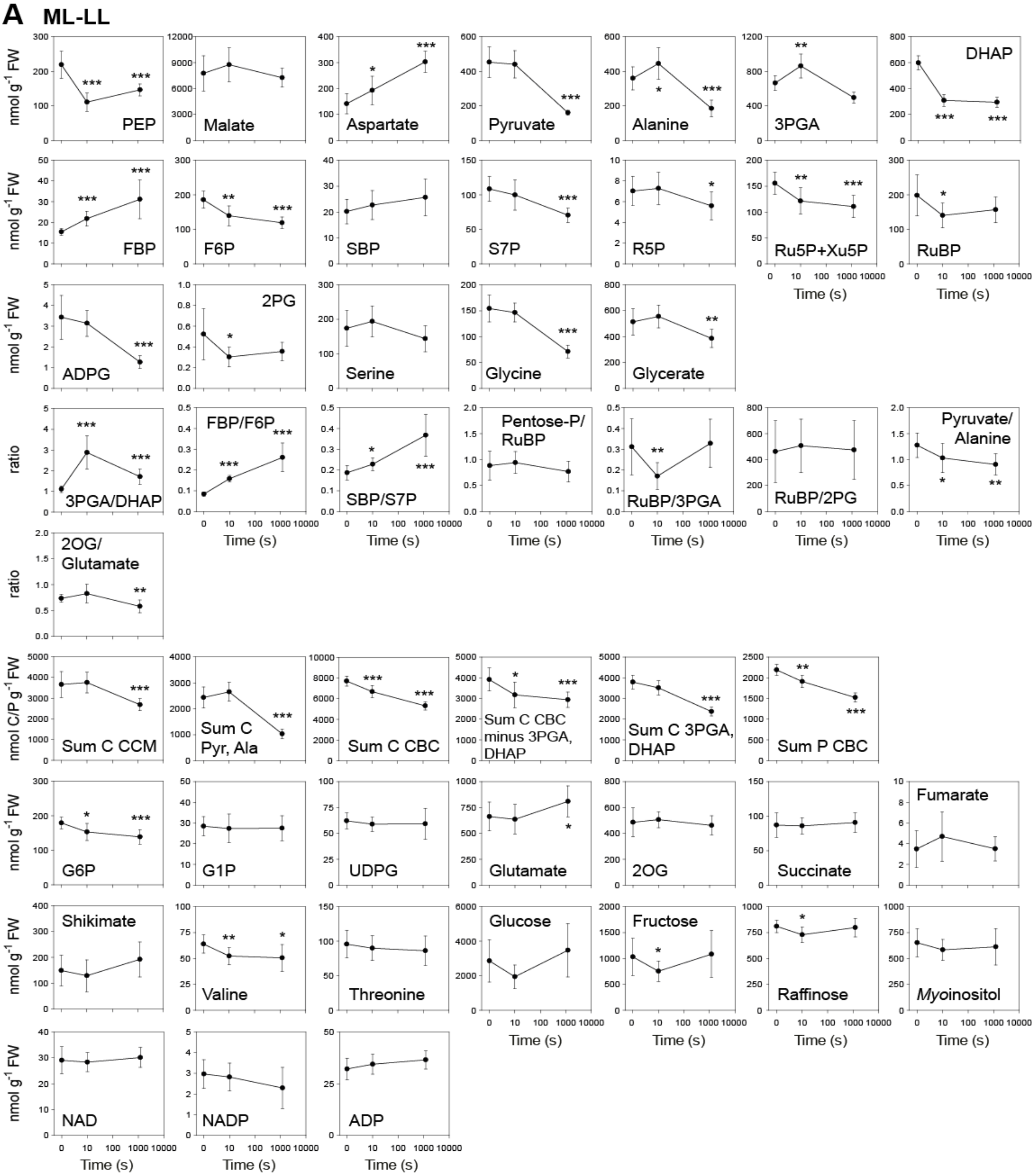

Supplementary Figure S5. Continued.

B **LL-ML**

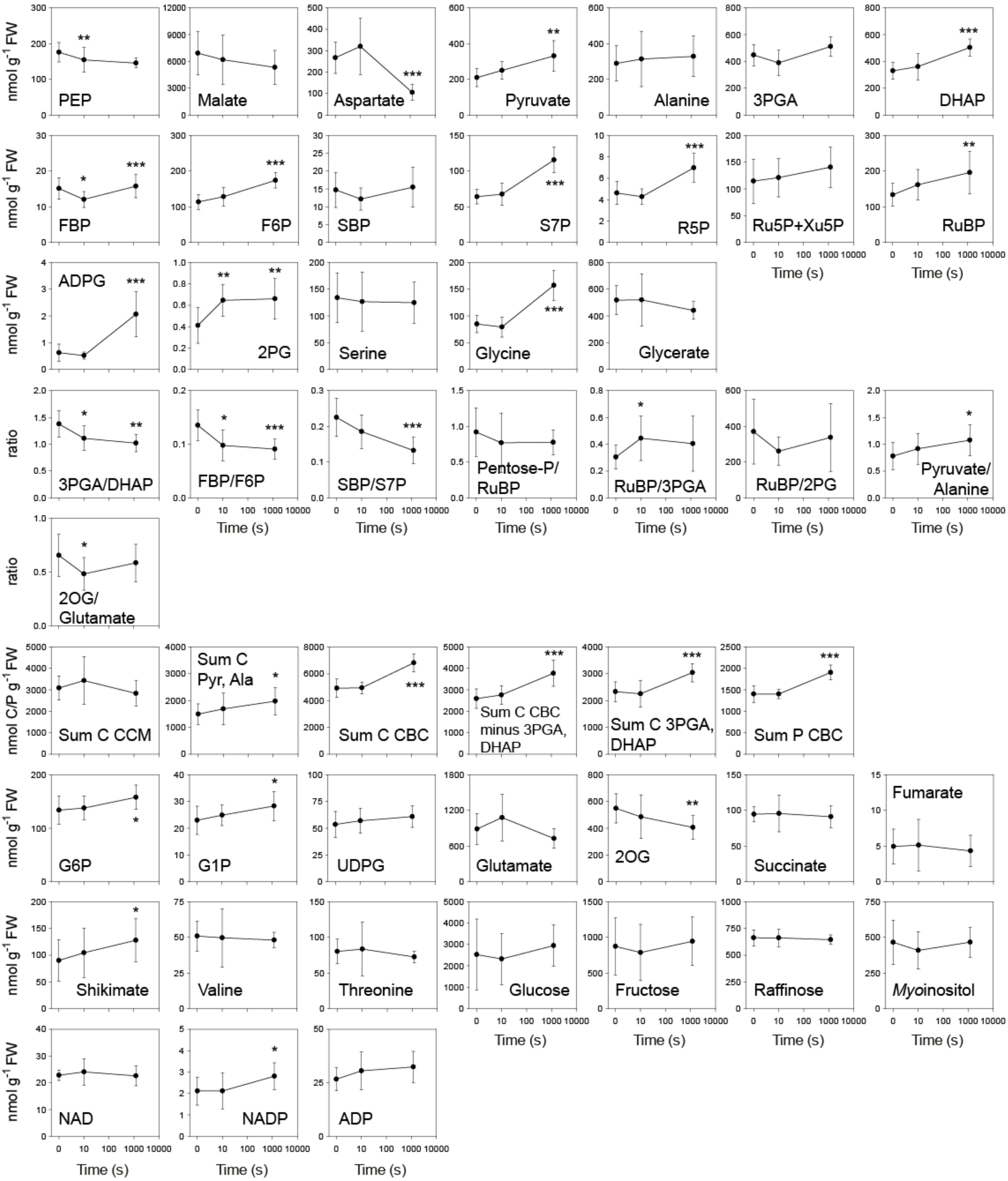

Supplementary Figure S5. Continued.

(C-D) Comparison of changes of individual metabolites, metabolite ratios and sets of metabolites at 10 s and 1200 s in the two experiments. The plots show the ratios at 10 s and 20 min to the average amounts at time 0 s (before light transition). Time 10 s is presented in light grey while time 20 min is presented in dark grey. The values obtained in the repetition with two time points are indicated with hatching.
(C) ML-LL transition
(D) LL-ML transition (plot overleaf**).**

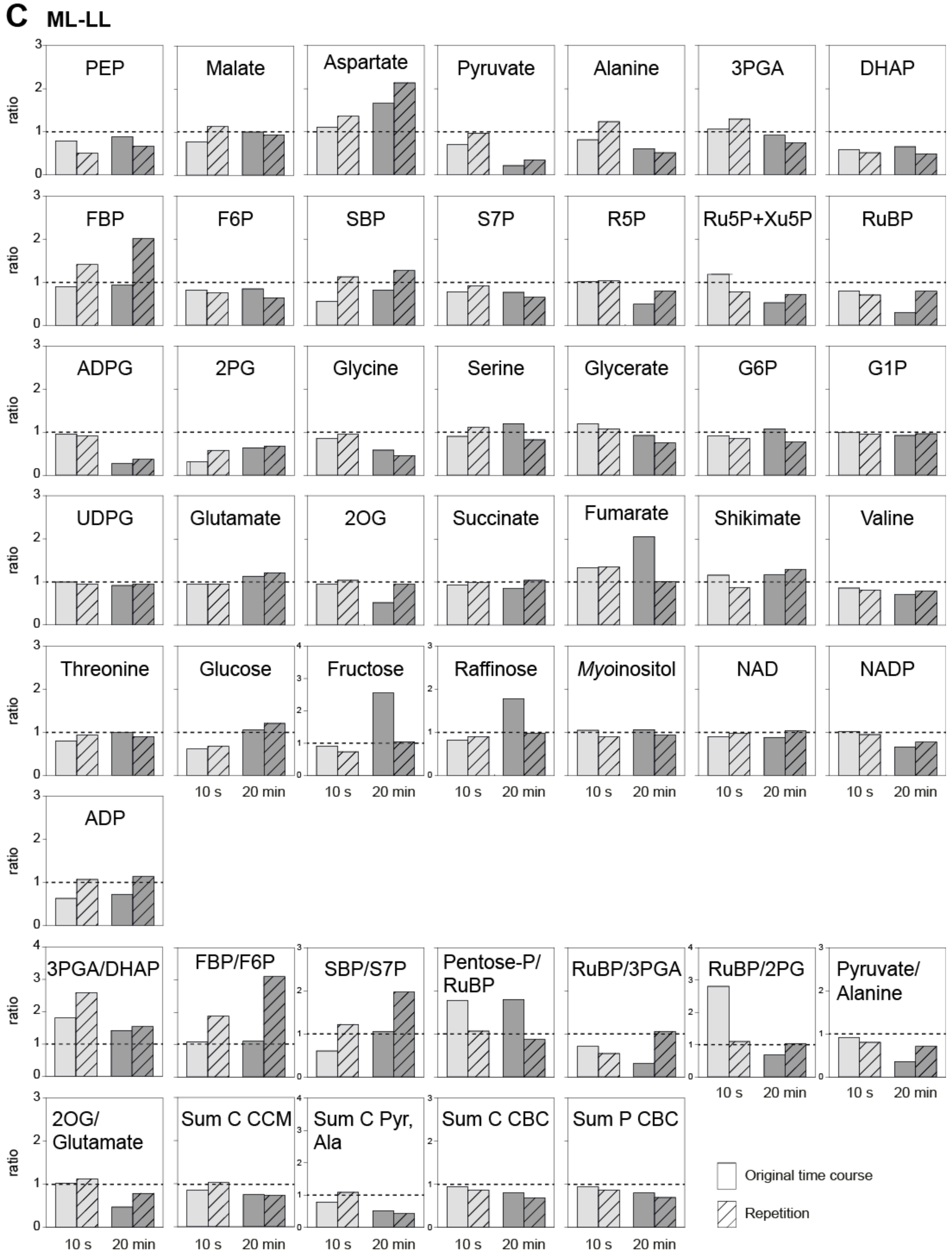

Supplementary Figure S5. Continued.

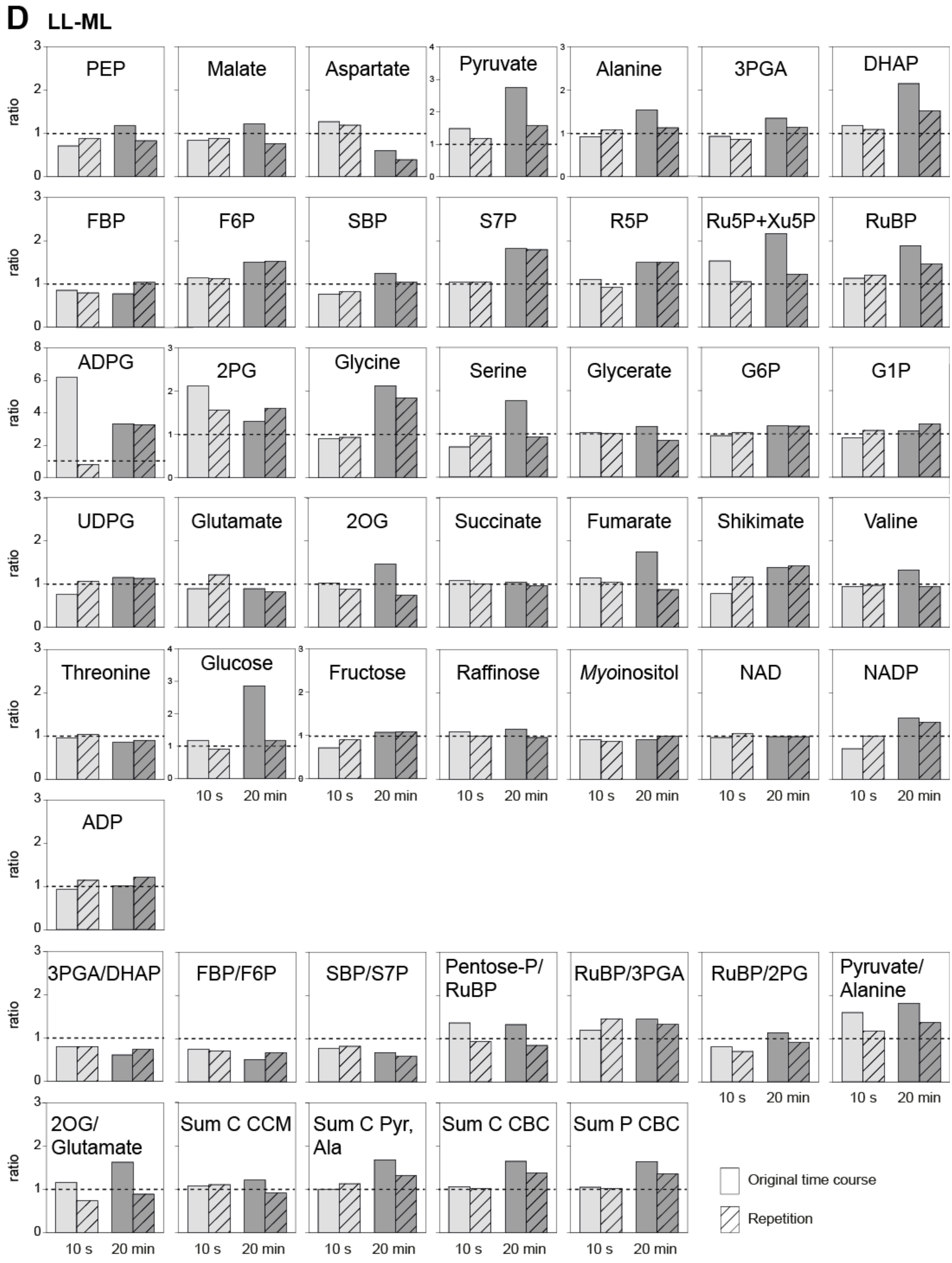

Supplementary Figure S6. Relationship between A_n and metabolite levels.

Heatmaps showing Pearson's correlation coefficients between A_n and metabolites in the (**A**) ML-LL transition and (**B**) LL-ML transition (plot overleaf). Pearson's correlation coefficients were calculated using individual samples at all time points after the transition to LL or the transition to ML (time zero excluded). Significant correlations ($p < 0.05$) are colored red (positive) and blue (negative), while correlations that were not significant are shown in grey. Self-correlations are identified in dark grey. The hierarchical clustering is shown. Panels A and B are expanded versions of Fig. 5A.

(C) R^2 and p values for a regression between A_n and metabolite levels, ratios or summed pool and **(D)** plots of summed C in the CCM vs CBC (plots overleaf, two pages later).

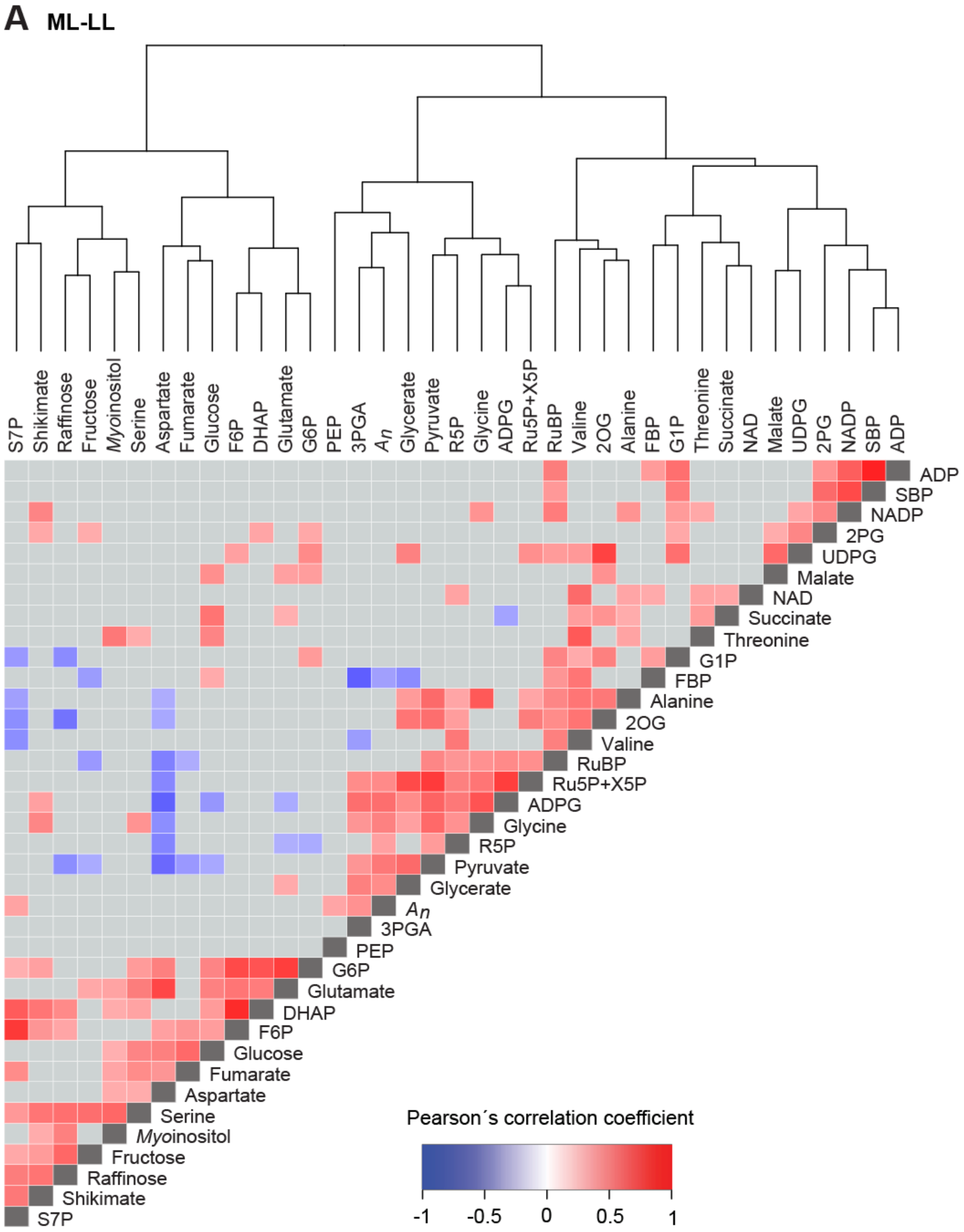

Supplementary Figure S6. Continued.

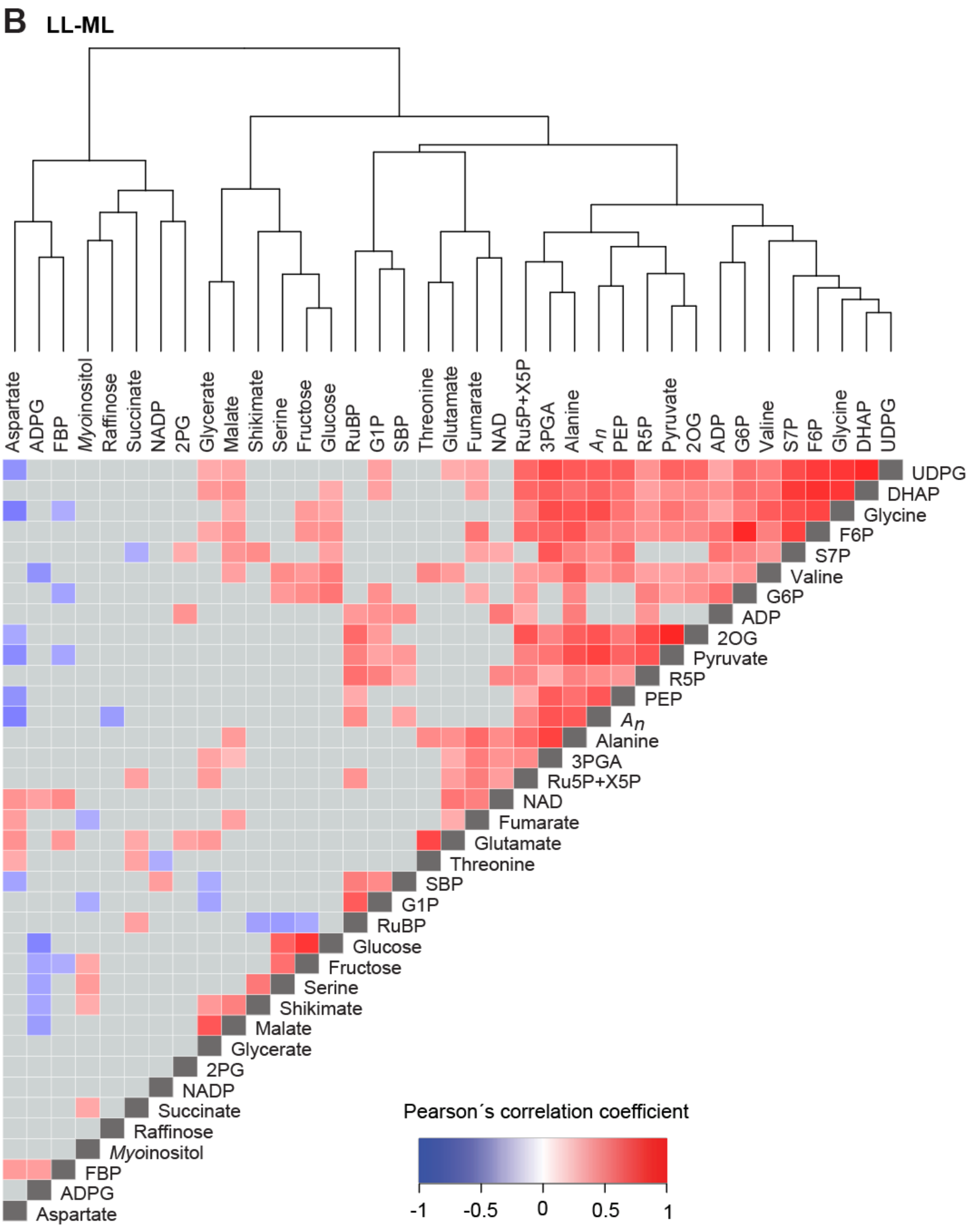

Supplementary Figure S6. Continued.

(**C**) Relation between A_n and metabolite levels or metabolic traits. A_n was plotted against metabolite rates using individual samples (*n*=3 to 5 for ML-LL and *n*=4 for LL-ML) at all points after the transition (time zero excluded). Additional plots are calculated from 300 to 1800 s for the ML-LL transition, corresponding to the slight recovery of A_n in the later part of the transient. The displays summarise R^2, the slope direction and p value (regression function with 95% confidence interval). The plots are provided in Supplementary Figs. S7 and S8.

(**D**) Relation between total metabolite pools in the CCM and the CBC. Analogous plots to panel C were made for summed C in metabolites in the CBC and CCM, or in the energy shuttle pools (3PGA and DHAP).

C

	ML-LL						LL-ML		
	5-1800 s			300-1800 s			5-1800 s		
Traits	R^2	slope	*p*	R^2	slope	*p*	R^2	slope	*p*
PEP	**0.12**	**+**	**0.03**	**0.33**	**+**	**0.03**	**0.44**	**+**	**0.00**
Malate	0.01	-	0.48	0.02	-	0.61	0.04	+	0.23
Aspartate	**0.06**	**-**	**0.10**	0.01	+	0.68	**0.24**	**-**	**0.00**
pyruvate	**0.24**	**+**	**0.00**	**0.31**	**+**	**0.02**	**0.55**	**+**	**0.00**
Alanine	0.02	+	0.43	**0.30**	**+**	**0.02**	**0.42**	**+**	**0.00**
Glutamate	0.00	-	0.73	0.14	+	0.12	0.01	-	0.50
2OG	0.01	+	0.48	0.01	+	0.71	**0.45**	**+**	**0.00**
3PGA	**0.18**	**+**	**0.00**	**0.56**	**+**	**0.00**	**0.46**	**+**	**0.00**
DHAP	0.00	-	0.70	0.04	-	0.41	**0.37**	**+**	**0.00**
FBP	**0.13**	**-**	**0.02**	**0.55**	**-**	**0.00**	0.08	-	0.08
F6P	0.02	+	0.35	0.03	-	0.48	**0.25**	**+**	**0.00**
SBP	0.03	-	0.31	0.19	+	0.07	**0.13**	**+**	**0.02**
S7P	**0.12**	**+**	**0.02**	0.00	-	0.92	**0.19**	**+**	**0.01**
R5P	**0.13**	**+**	**0.02**	**0.53**	**-**	**0.00**	**0.20**	**+**	**0.00**
Ru5P + X5P	**0.21**	**+**	**0.00**	0.13	-	0.14	**0.21**	**+**	**0.00**
RuBP	0.00	-	0.70	**0.31**	**-**	**0.02**	**0.20**	**+**	**0.00**
ADPG	**0.32**	**+**	**0.00**	0.11	-	0.17	0.01	+	0.48
2PG	0.01	-	0.58	0.00	+	0.97	0.02	-	0.38
Serine	0.01	+	0.60	0.19	+	0.07	0.02	-	0.38
Glycine	**0.23**	**+**	**0.00**	0.04	+	0.43	**0.48**	**+**	**0.00**
Glycerate	**0.20**	**+**	**0.00**	0.21	+	0.05	0.05	+	0.16
3PGA/DHAP	**0.15**	**+**	**0.01**	**0.34**	**+**	**0.01**	0.01	-	0.55
FBP/F6P	**0.15**	**-**	**0.01**	**0.41**	**-**	**0.00**	**0.18**	**-**	**0.01**
SBP/S7P	0.06	-	0.12	0.13	+	0.14	0.00	-	0.94
Pentose-P/RuBP	**0.10**	**+**	**0.05**	0.14	+	0.13	0.03	-	0.29
RuBP/3PGA	0.05	-	0.14	**0.45**	**-**	**0.00**	0.03	+	0.26
RuBP/2PG	0.00	-	0.76	0.01	-	0.63	0.08	+	0.07
Pyruvate/Alanine	**0.30**	**+**	**0.00**	0.04	+	0.45	**0.12**	**+**	**0.03**
2OG/Glutamate	0.01	+	0.57	0.01	-	0.67	**0.40**	**+**	**0.00**
Sum C in CCM	0.01	+	0.58	0.14	+	0.18	**0.20**	**+**	**0.00**
Sum C in Pyruvate + Alanine	0.06	+	0.12	**0.33**	**+**	**0.02**	**0.50**	**+**	**0.00**
Sum C in CBC	**0.19**	**+**	**0.00**	0.11	+	0.17	**0.55**	**+**	**0.00**
Sum C in CBC minus 3PGA + DHAP	**0.09**	**+**	**0.05**	**0.23**	**-**	**0.04**	**0.39**	**+**	**0.00**
Sum C in 3PGA + DHAP	**0.16**	**+**	**0.01**	**0.45**	**+**	**0.00**	**0.51**	**+**	**0.00**
Sum P in CBC	**0.17**	**+**	**0.01**	0.19	+	0.07	**0.57**	**+**	**0.00**

D

C in CBC vs C in CCM	0.00	+	0.90	0.03	-	0.57	**0.37**	**+**	**0.00**
C in CBC vs C in pyruvate + alanine	0.03	+	0.28	0.03	-	0.53	**0.56**	**+**	**0.00**
C in 3PGA + DHAP vs C in CBC minus 3PGA + DHAP	**0.13**	**+**	**0.02**	0.00	-	0.85	**0.45**	**+**	**0.00**

	p-value		
	<0.05	<0.01	<0.001
UP	*	**	***
DOWN	*	**	***

Supplementary Figure S7. Additional plots of metabolite levels and metabolic traits against A_n in a transition from moderate to low light. This figure is Supplementary to Figs. 5-6.

(**A**) Metabolite levels**,**

(B) Metabolite ratios, and

(C) Sum of C in CCM and sums of C and P in CBC.

The design of the plots is described in the legend of Fig. 5. Briefly, the insert in shows times between 300-1800 s (dotted grey box in main panel), with an expanded scale for A_n (52 – 56 µmol CO_2 g^{-1} FW s^{-1}) to visualize relationships during the slight recovery of A_n after 250 s. Arrows denote the time sequence in the main plot and in the insert. Slope directions and p values were calculated by linear regressions using individual samples at all time points after the transition (time zero excluded) and also from 300 to 1800 s (in insert). The p value of the correlation is given, colored according to the direction of the slope.

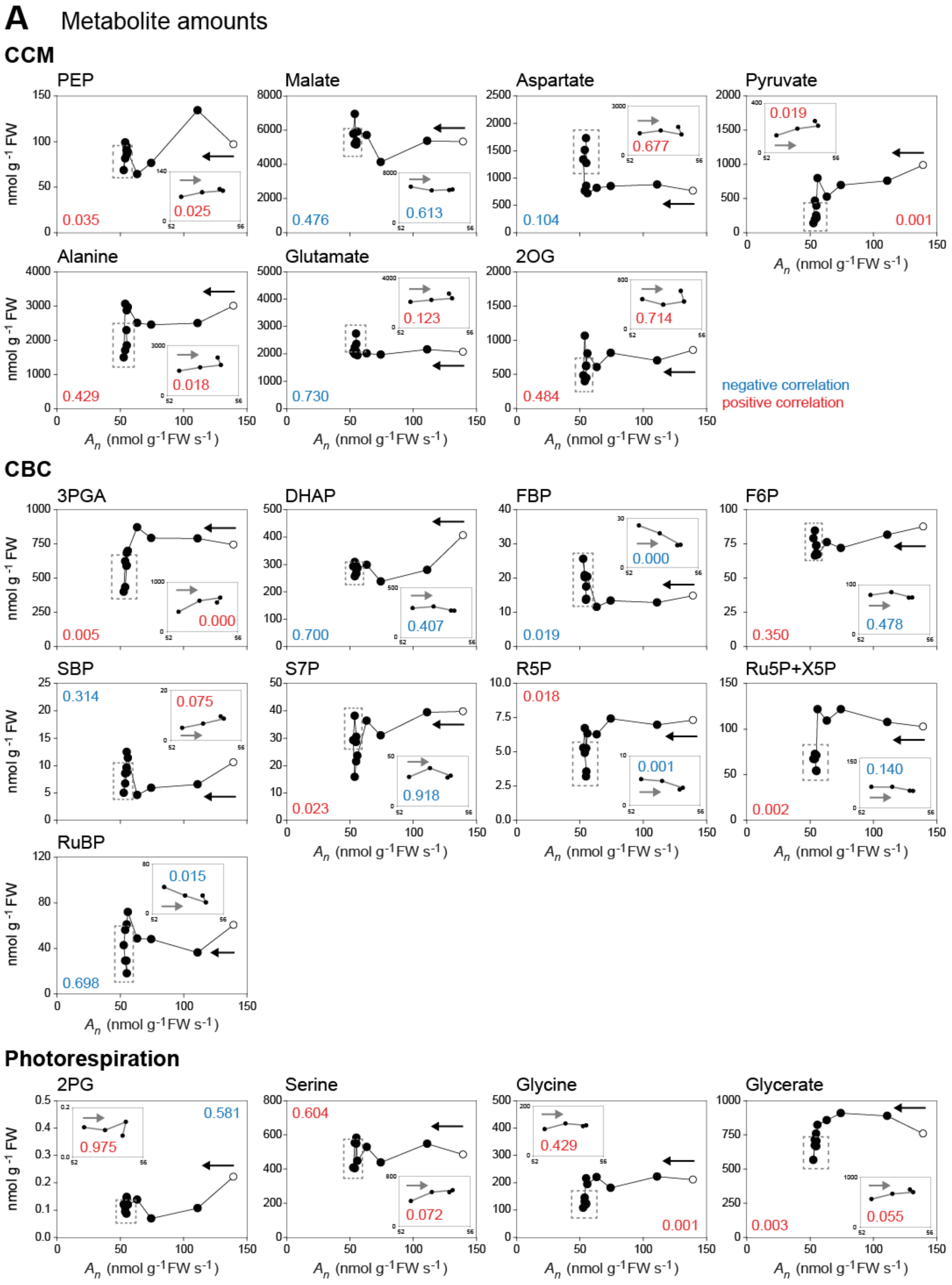

Supplementary Figure S7. Continued.

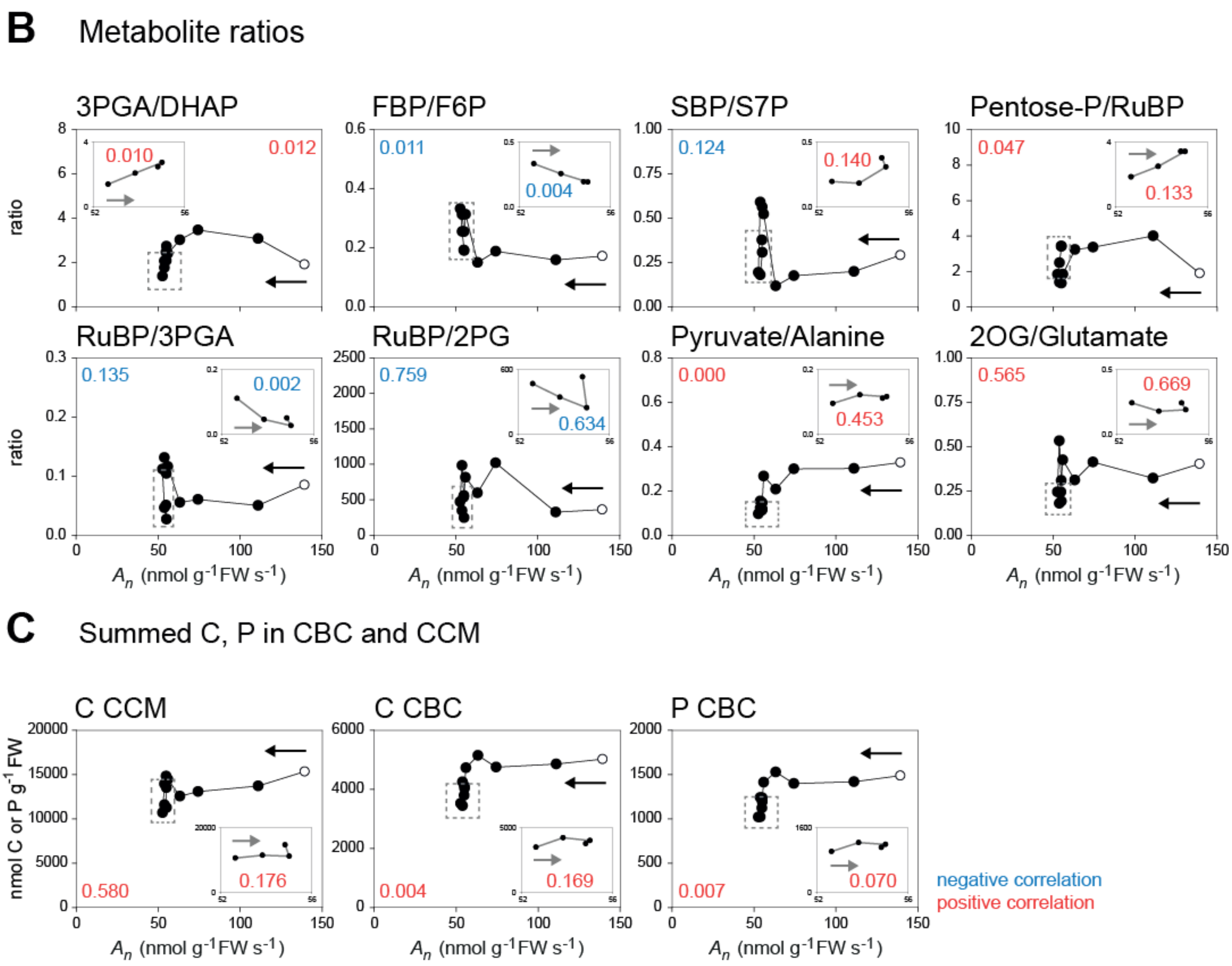

Supplementary Figure S8. Additional plots of metabolite levels and metabolic traits against A_n in a transition from low to moderate light. This figure is Supplementary to Figs. 5-6.

(**A**) Metabolite levels**,**

(B) Metabolite ratios**,**

(C) Sum of C in CCM and sums of C and P in CBC**.**

The design of the plots is described in the legend of Fig. 5. Arrows denote the time sequence. Slope directions and *p* values were calculated by linear regressions using individual samples at all time points after the transition (time zero excluded). The *p* value is colored according to the direction of the slope.

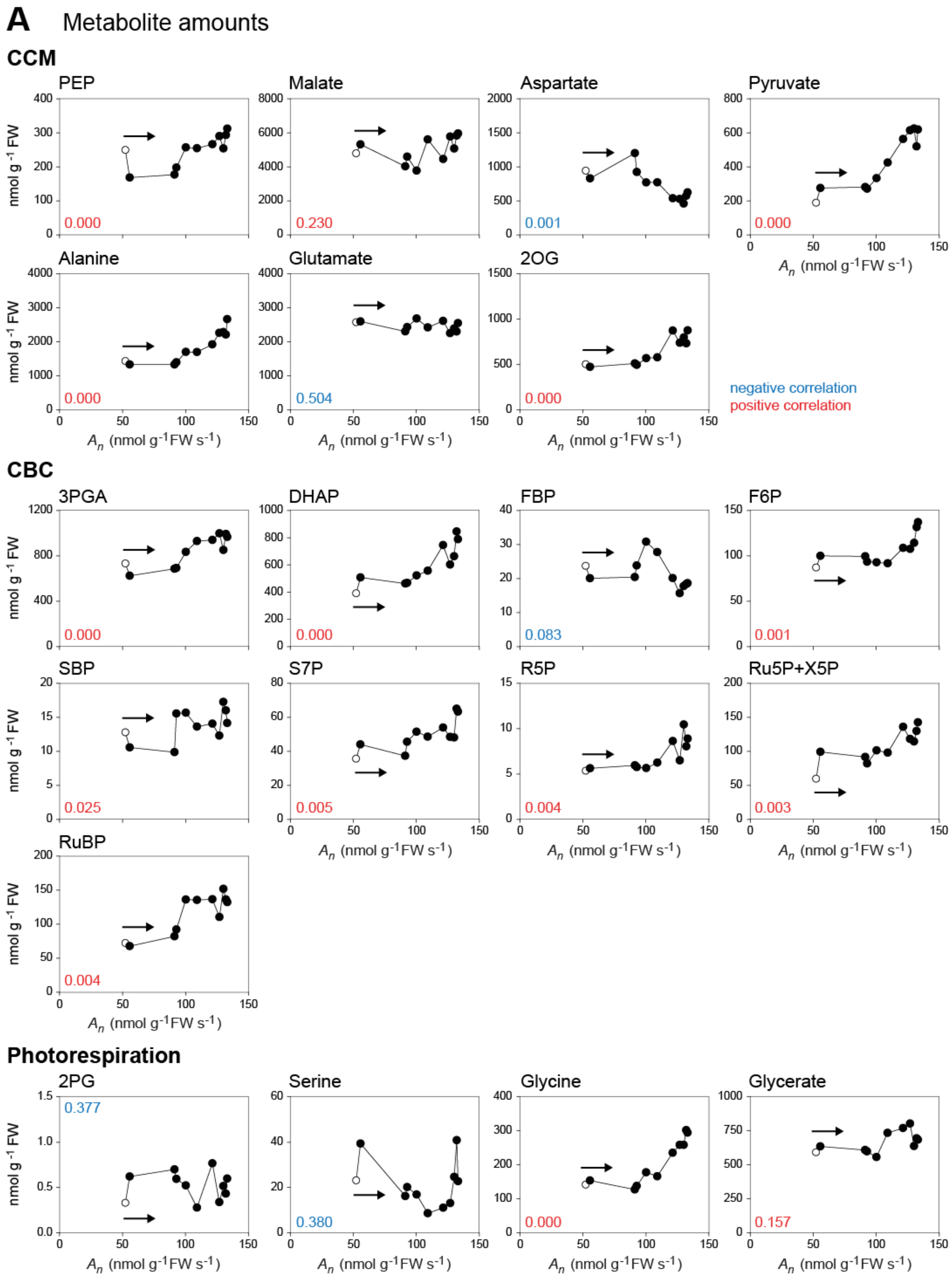

Supplementary Figure S8. Continued.

B Metabolite ratios

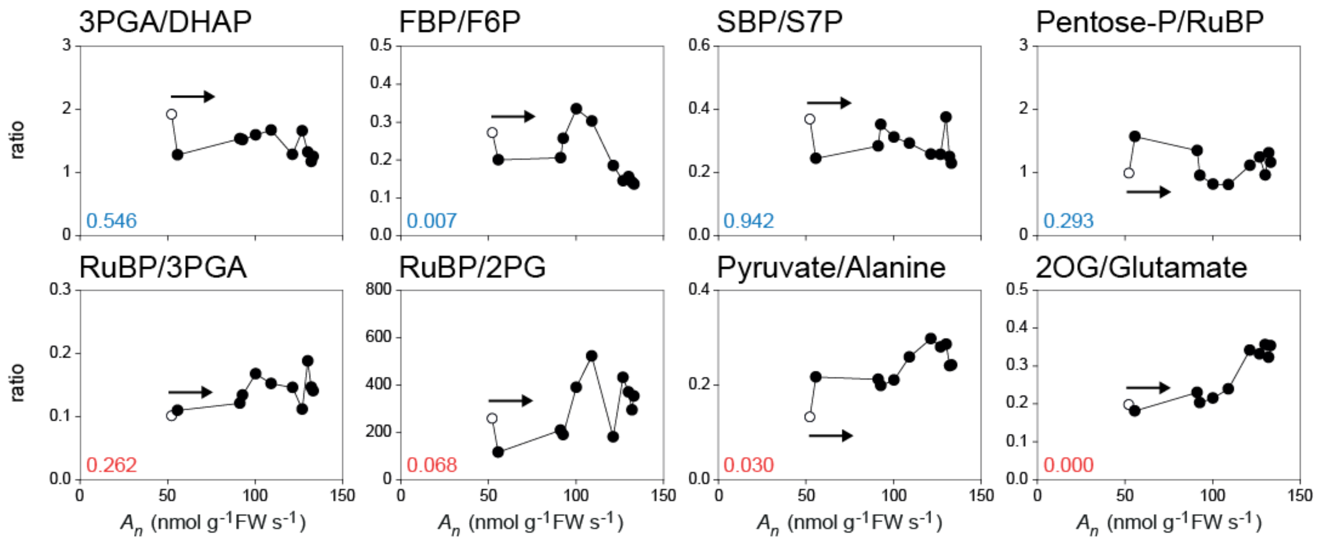

C Summed C, P in CBC and CCM

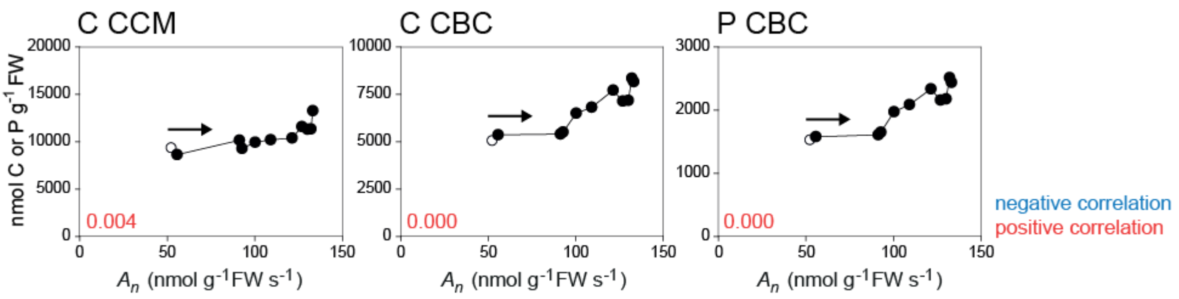

Supplemental Text

1. Gas exchange of plants sampled for metabolite measurements.

(Supplementary to Results sections '*Response of CO_2 assimilation and stomatal conductance', 'Global analysis of response of metabolism'*

The gas exchange data presented in Fig. 1 and Supplemental Dataset S1A were logged every second, and corrected by dynamic equations (Saathoff and Welles, 2021) to account for the lack of steady state. The effects of these corrections are shown in Supplementary Fig. S1J, revealing a clear impact of the correction just after the switch in light intensity for both transitions.

Metabolite measurements were made in samples harvested from separately-grown batches of maize. For technical reasons, gas exchange in this batch of plants was recorded at 6 s intervals and correction was not possible as each time point was a recorded running average (Supplementary Dataset S1B). Comparison of these data with the uncorrected data underlying Fig. 1 recorded every second showed good agreement between the two independent experiments (Supplementary Fig. 1K). In particular, in the ML-LL, the initial decay of A_n was delayed, and the trough at ~250 s and subsequent slight recovery of A_n observed in the experiment where data were logged every second (Fig. 1C) was also observed in the experiment where data were recorded every 6 seconds (Supplementary Fig. S1K) with the recovery starting in this case from a slightly earlier trough at ~200 s (Supplementary Fig. S1L, significant at p= 0.03, paired *t*-test). In the LL-ML transition the slowing down of the rise at about 90 s and subsequent slow further rise until 1000 s was observed in both experiments (Supplementary Fig. S1K). The early plateau observed in the LL-ML transition at 11-15 s with corrected data (Fig. 1B) was visible as a slowing of the rise of A_n in both uncorrected datasets (Supplementary (Fig. S1K).

2. Rate of exchange of C between the CBC (including the energy shuttle metabolites) and the CCM

Supplemental to the main Discussion sections' *Response of the CBC and CCM to a sudden decrease in irradiance*' and '*Response of the CBC and CCM to an increase in irradiance*'

2.1 Background

The CBC (including the energy shuttle intermediates 3PGA and DHAP) and the CCM are nominally independent pathways with no shared metabolites. However, exchange of C between the CBC and the CCM metabolites can occur via equilibration of 3PGA and PEP. These two metabolites are interconverted via near-equilibrium reactions catalyzed by phosphoglycerate mutase and enolase. When operating in steady state, the CBC and CCM can form closed cycles. In the CBC, 3PGA is formed by Rubisco and either reduced in the BSC or moves to the MC where it is reduced. In the CCM, PEP is the substrate for PEPC, and is regenerated from pyruvate in the PPDK reaction. However, the near-equilibrium reactions catalyzed by phosphoglycerate mutase and enolase provide the potential to move C between the CBC (and energy shuttle)

and the CCM (see sketch below, the steady state corresponds to the scenario in the left-hand panel of the diagram overpage). This potential is underlined by the 3-to 4-fold higher activity of phosphoglycerate mutase and enolase in the MC compared to the BSC (Ku and Edwards 1975; Furbank and Leegood 1984).

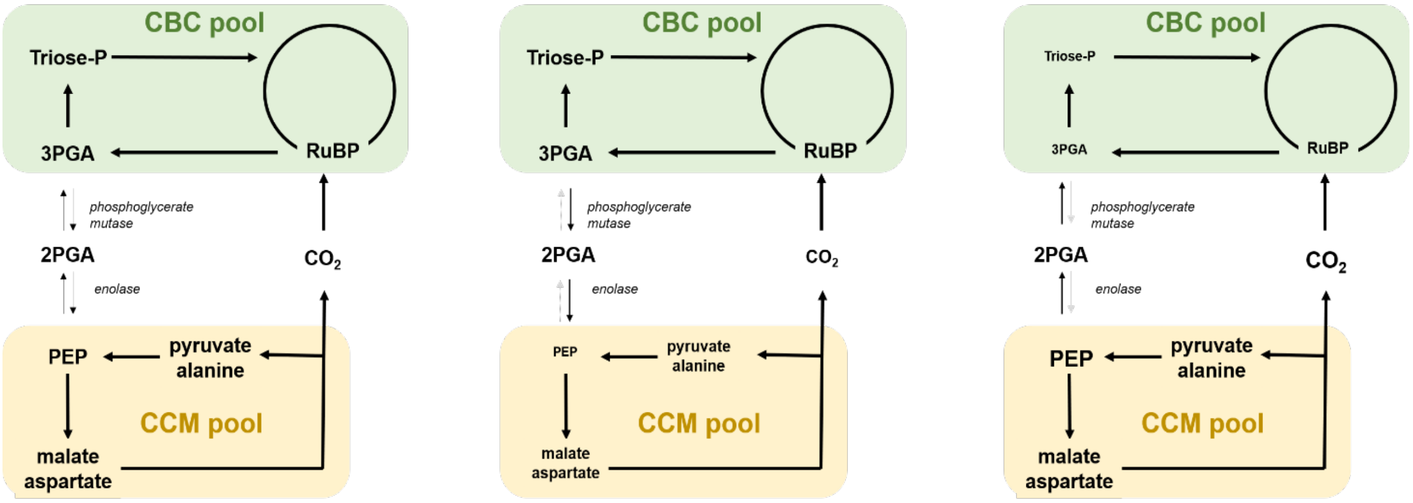

Stead state

Phosphoglycerate and enolase catalyse forward and reverse reactions at the same speed

No net flux between CBC and CCM

Perturbation

CCM pool is low compared to CBC pool

PEP decreases relative to 3PGA
Phosphoglycerate mutase and enolase flux in direction of PEP

C moves from the CBC to the CCM pool

Perturbation

CCM pool is high compared to CBC pool

PEP increases relative to 3PGA
Phosphoglycerate mutase and enolase flux in direction of 3PGA

C moves from the CCM to CBC pool

Based on ^{13}C labelling kinetics, Medeiros et al. (2022) recently estimated that during steady state photosynthesis phosphoglycerate mutase and enolase exchange ^{13}C at a rate equivalent to 18 and 22% of the net rate of CO_2 fixation at an irradiance of 550 and 160 µmol^{-2} m^{-2} s^{-1}, respectively. Exchange of label does not, however, mean that there is a net flux of C. At thermodynamic equilibrium, when no net flux occurs, the 3PGA:PEP ratio would be about 3:1 (based on the theoretical equilibrium constants of phosphoglycerate mutase and enolase of 0.17 and 2.07, Stryer 1990). This may be modified by conditions in the cellular milieu like pH and Mg^{2+} levels, as these differentially affect ionization and ion binding and thus the concentration of the actual substrate. Further, due to compartmentation, the overall level of 3pGA and PEP may not precisely reflect cytoplasmic levels in the MC. One potential complication could be differing distribution of 3PGA and PEP between the BSC and MC. The second and probably more important complication is their subcellular distribution between the chloroplast stroma and cytosol. Due to the absence of phosphoglycerate mutase and enolase from the plastid stroma of chloroplasts in mature leaves (Stitt and ap Rees 1980; Fukuyama et al. 2015), whilst the stroma contains a large pool of 3PGA (participating in the CBC) the PEP pool in the stroma is small (Szecowka et al. 2013). As a result, the overall 3PGA/PEP ratio in leaf material will overestimate the 3PGA/PEP ratio in the MC cytosol, the place where interconversion of 3PGA and PEP will directly impact on C flow towards or away from PEPC. Detailed studies of metabolite levels across a range of conditions in maize and Amaranthus in which 3PGA and PEP levels varied over a <10-fold range revealed that the relationship is maintained very constant, with a 3PGA:PEP ratio of about 4.6 (Leegood and von Caemmerer 1989). In our current studies, the measured levels 3PGA and PEP in maize leaves in steady state condition were close to or above the theoretical 3:1 ratio (Figs. 3A, 4A). Thus, whilst labeling studies

demonstrate there is rapid movement of C from 3PGA into PEP (Medeiros et al. 2022), in steady state conditions this will be largely balanced by flow of C from PEP back to 3PGA.

Net flux of C will occur after perturbations that alter the rate of photosynthesis and/or alter the balance between CBC and CCM pool size. After perturbations that increase the level of 3PGA relative to PEP (or more precisely, when the 3PGA/PEP level in the MC cytosol rises above the ratio found at thermodynamic equilibrium) there will be an increase in next flux from 3PGA to PEP (middle scenario in above sketch). After perturbations that decrease in the level of 3PGA relative to PEP (or more precisely, when the 3PGA/PEP level in the MC cytosol falls below the ratio found at thermodynamic equilibrium) there will be net flux from PEP to 3PGA (right hand scenario in above sketch).

Based on the rate of label exchange in steady state photosynthesis in Medeiros et al. (2022), and estimated for the most extreme scenario:

i) when the 3PGA/PEP ratio is very high and phosphoglycerate mutase and enolase catalyze almost exclusively reactions in the direction that converts 3PGA to 2PGA to PEP, this flux could generate a ~10% decrease in the total CBC pool (including 3PGA and triose-P) in about 25 s under an irradiance of 550 µmol m^{-2} s^{-1}, with slightly longer being required under an irradiance 160 µmol m^{-2} s^{-1}.

ii) when the 3PGA/PEP ratio is very low and enolase and phosphoglycerate mutase catalyze almost exclusively reactions in the direction that converts 3PEP to 2PGA to 3PGA, the flux could generate a ~10% increase in the total pool of CCM metabolites in about 36 s under an irradiance of 550 µmolm^{-2} s^{-1}, with slightly longer being required under an irradiance 160 µmol m^{-2} s^{-1}.

These calculations are based on measured values of A_n in ML (~ 135 nmol C g^{-1} FW s^{-1}) and the summed pool of CCM metabolites and summed pool of CBC plus energy metabolites (8200 and 6250 nmol C g^{-1} FW, respectively).

It should be noted that the summed pool for CCM metabolites is underestimated because it does not include malate (the active pool of malate cannot be well determined due to the presence of two or more pools of malate in maize leaves, with the pool that is directly involved in the CCM being only a small component, probably less than 20% (see Arrivault et al. 2017; Medeiros et al. 2022). Consequently, the estimated time for a ~10% increase in the total pool of CCM metabolites of about 36 s is a minimum value.

2.2 Implications for movement of C between CBC and CCM pools in the ML-LL transition

During a ML-LL transition, the summed C in the pool of CBC plus energy metabolites fell in the first 120 s (Fig. 3C), followed slightly later (120-300 s) by a fall in the summed C in the estimated pool of CCM metabolites, especially pyruvate and alanine (Figs. 3A, 3C) (see main text and below, Supplementary text Section 3.1). Interestingly, this slightly delayed drop of the CCM metabolites started after a delay.

This corresponded with changes of 3PGA and the 3PGA/PEP ratio (overpage). 3PGA remained high at 10-15 s whereas PEP showed a significant decrease at 10 and 15 s, leading to an initial increase of the 3PGA/PEP ratio (compare plots in Fig. 3A, also plot of the 3PGA/PEP ratio alongside, T-test indicated as in

Fig. 3). An initial rise of the 3PGA/PEP ratio was also seen after switching to low light in a 300 s fluctuating regime (Sales et al. 2025). This rise would initially prevent net loss of C from the CCM pools and favor flux from 3PGA to PEP

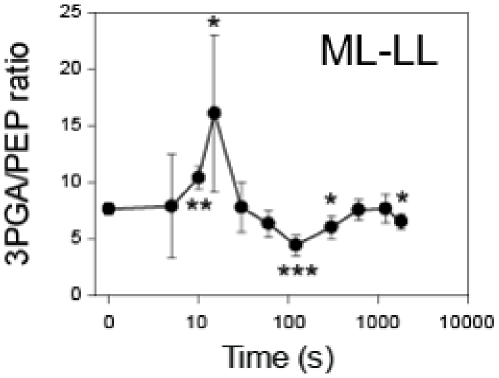

From 30 s onwards, 3PGA decreased both in absolute terms (Fig. 3) and relative to the PEP (Fig. 3A) leading to a decrease of the 3PGA/PEP ration (see plot alongside). The delay in the decay of the CCM pool (see main text and Supplementary text Section 3.1 below) was probably due at least in part to the delay until 3PGA decreased, and the slowness of the decay to the 3PGA/PEP ratio decreasing by only about two-fold compared to steady state ML or LL as well as the rather restricted capacity for flux over phosphoglycerate mutase and enolase (see previous subsection).

The complex time course of the 3PGA/PEP ratio at least partly explains the rather uncoordinated responses of the CBC, energy shuttle and CCM pools during the ML-LL transition (Fig. 7B). Nevertheless, by 300 s there was a substantial decrease in both the CBC and the CCM pools (by 1500 and ~5000 nmol C g^{-1} FW, respectively, accounting for ~30 and ~33%, respectively, of the initial pool size in ML; see Fig. 1C and Supplementary Dataset S2).

As described in the main text and below in Supplementary Text Section 3.1, the initial decline of CBC and CCM metabolite pools is partly reversed later in the ML-LL transition. The slight (7%) but highly significant rise in A_n (Fig. 1C) was accompanied by a ~20% increase in the total size of CBC pool, due mainly to an increase in the energy shuttle intermediates 3PGA and DHAP, and a ~70% rise in the C in CCM metabolites (Fig. 3C; Supplementary Fig. S3B). As also mentioned in Supplementary Text Section 2.1, the partial recovery of the CBC and CCM pools together accounted for about 10% of the CO_2 fixed during the recovery phase. This increase was mostly due to the rise in the CCM pool (from 10,339 to 14,828 nmol C g^{-1} FW) compared to a smaller and possibly earlier rise of the CBC metabolite pool (from 3,443 nmol C g^{-1} FW at 120 s to a peak of 4,252 nmol C g^{-1} FW at 600 s, that was followed by a plateau or slight decline) (Fig. 1C, for details see Supplementary Dataset S2). Thus, during this recovery, over 8% of the fixed C is moved from the CBC pool via phosphoglycerate mutase and enolase into the CCM pool. It is noteworthy that during this slight recovery, the 3PGA level rises more markedly than the PEP level (see Fig. 3A and insert above), which would favor increased net flux from the CBC pool to the CCM pool.

Many factors may contribute to the differing kinetics of 3PGA and PEP and the resulting changes in the 3PGA/PEP ratio. The following is a plausible explanation. The initial rise of the 3PGA/PEP ratio is partly due to a significant decrease of PEP at 10 and 15 s, probably due to consumption of PEP by PEPC exceeding regeneration of PEP by PPDK (see main manuscript and Supplementary Section 3.1). At the same time, 3PGA trends upwards until 15 s, reflecting its formation from oxidation of DHAP that results from the shortfall of NADPH and ATP after a drop in irradiance (see Supplementary Section 3.1). The subsequent decline of the 3PGA/PEP ratio at 60-300 s is driven by a decline of 3PGA, due to a decline of energy shuttle pools in the low irradiance, exacerbated by the overshoot in end product synthesis (see main manuscript) The rise of

the 3PGA/PEP ratio between 300-1800 reflects recovery from this depletion of 3PGA, whilst the smaller rise in PEP may imply that PEPC activity has been upregulated.

Summarizing, the CCM and CBC metabolite pools change in an uncoordinated manner in the ML-LL transition, both i) early in the transition as A_n is rapidly decreasing, when the CCM pool declines later than the CBC pool and ii) later in the transition as A_n recovers slightly, when the CCM pool rises later but more markedly than the CBC pool (see main text and Supplementary Text Section 3.1) . In both cases, the differing response can be at least partly explained by changes in the 3PGA/PEP ratio that reflect the constraint on flux over the enolase and phosphoglycerate mutase reaction and are, on the one hand, indicative of an imbalance between the CCM and CBC and, on the other hand, determine via mass action the net direction and rate of C exchange between the CBC and CCM.

2.3 *Implications for movement of C between CBC and CCM pools in the LL-ML transition.* During an LL-ML transition, the summed C in the pool of CCM metabolites and the summed C in the pool of CBC plus energy metabolites rise gradually (Fig. 4C), with the CBC pool (excluding 3PGA and DHAP) rising most in the first part of the response (until about 100 s) and the energy shuttle and CCM metabolites increasing most in the later part of the response from about 100 s onwards (Fig. 4,6C; Supplementary Fig. S4B; Supplementary Text Section 3.2). Thus, in contrast to the ML-LL transition, these pools change in a rather coordinated manner (see Fig. 7).

This matches the response of the 3PGA/PEP ratio. After an initial ~30% significant increase at 10 s increase of the 3PGA/PEP ratio, due to PEP decreasing more than 3PGA (Fig. 4A), 3PGA and PEP levels rise gradually, and roughly in parallel (Fig. 4A) and the 3PGA/PEP ratio remains stable. The trend to a slightly elevated 3PGA/PEP ratio than in LL is consistent with the gradual increase of the CCM pools in the later part of the LL-ML transition.

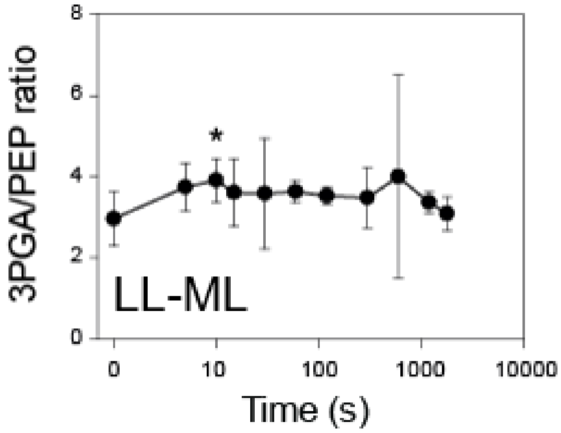

Incidentally, in a 30 s fluctuating light regime, the 3PGA/PEP ratio even decreased after 10 s and (marginally) 30 s in high light (Sales et al. 2025). This may reflect different poising after a relatively short and long time in low light, or the different light intensities used in the LL-ML transition and in Sales et al. (2025)

3. Detailed account of changes in metabolism during the ML-LL and LL-ML transitions

3.1 Supplementary to section '*Response of the CBC and CCM to a decrease in irradiance'*

- *Transient buffering of CO_2 assimilation is quantitatively accounted for by energy released in transformations of large pools of shuttle metabolites,*

The first phase of the ML-LL transition captured in the PC analysis (Fig. 2A) lasted until about 15 s. This coincides with the time when, instead of falling immediately, there is a progressive decline in A_n (Fig. 1A;

Supplementary Fig. 1C). In this time most CBC metabolites including RuBP remained high and ADPG rose (Fig. 3A), which is consistent with continued operation of the CBC. This confirms the prediction of Stitt and Zhu (2014) that after a decrease in irradiance the large pools of metabolites in the energy shuttle and the CCM temporarily buffer C_4 photosynthesis against the shortfall of ATP and NADPH from the light reactions. However, this is only qualitative evidence. The key question is: are the changes in metabolite levels large enough to provide the energy that is required to support the observed buffering of A_n.

Relatively high (over 50% of that in ML, or over 30% of the difference between ML and steady state LL, ΔA_n,) was sustained until 10 s, and substantial rates until 15 s after switching to LL (A_n over 40% of that in ML, or over 25% of ΔA_n) (Fig. 1; Supplementary Fig. S1C). The response until 10 s is equivalent to buffering against the decrease in irradiance for about 4.5 s (Fig. 8A). This is a conservative estimate, because it excludes the continued slow decay of A_n after 10 s (at 15 s it would be equivalent to buffering A_n for about 6 s). Previous studies in C_4 plants including maize also reported that CO_2 assimilation continues for a time after darkening (Laisk and Edwards 1998), after imposing and then removing a short period of high light to imitate a sun fleck (Krall and Pearcy 1993) and in a fluctuating light regime after switching to low light (Lee et al. 2022; Arce-Cubas et al. 2023b, Sales et al. 2025).

The energy requirement will depend upon whether CO_2 is only fixed by PEPC into C_4 acids, or whether CO_2 is being assimilated by the CBC. In a detailed study of light-dark transients, Laisk and Edwards (1998) employed a custom-built gas exchange set up to investigate the impact of the rate and duration of photosynthesis in the preceding light treatment on post-illumination CO_2 assimilation. They concluded that post-illumination CO_2 assimilation was driven by pools of metabolites that were built up in the light, argued that it involved flux at PEPC, and that the amount of CO_2 fixed after darkening was limited by the amount of PEP formed from triose-P and 3PGA. In contrast, in our ML-LL switch the changes in metabolite levels including the maintenance of high RuBP and other CBC intermediate levels and high ADPGlc in the first 15-30 s after the decrease in light intensity (Figs. 3A, 3C) show that not only PEPC but also the CBC remains more active than in steady state low light. This does not, of course, show that all the CO_2 fixed at rates above steady state LL is continuing on into the CBC.

Continuation of A_n including operation of the CBC at rates above those in steady state LL requires additional ATP and NADPH to that provided by the light reactions in LL. The additional cost until 10 s can be estimated as that required to support CO_2 fixation for 10 s at a rate of ~36 nmol g^{-1} FW s^{-1} (45% of ΔA_n, where ΔA_n is 81 nmol g^{-1} FW s^{-1}). Taking just costs in the CBC, this represents about 110 nmol ATP g^{-1} FW and 72 NADPH nmol g^{-1} FW. This will be an overestimate if part of the additional CO_2 fixed in first seconds in LL remains in C_4 acids, rather than being released and reassimilated in the CBC (see below). On the other hand, some additional energy would still be required if NADP-MDH and/or PPDK continue to operate for a short time at a higher rate than in steady state LL.

DHAP decreased significantly by ~180 nmol g^{-1} FW in the first 10 s after decreasing irradiance (Fig. 3A, Supplementary Fig. S3B) and the 3PGA/DHAP ratio almost doubled (Fig. 3C, Supplementary Fig. S3B). Assuming that the decrease of DHAP is mainly due to oxidation of DHAP to 3PGA, it would generate up to

180 nmol g^{-1} FW of ATP and NADPH. This is a maximum value because some DHAP will be consumed in other reactions, including continued use for RuBP regeneration by chloroplast FBPase, and continued conversion to sucrose by cytosolic FBPase. Corroborative evidence that much of the DHAP was converted to 3PGA is provided by the upwards trend of 3PGA during the first 15 s in LL (~120 nmol g^{-1} FW between ML and 15 s in LL, Fig 3A). The rise of 3PGA probably underestimates how much DHAP is converted to 3PGA because increased 3PGA in combination with the significant 35% decline in PEP (Fig. 3A, Supplementary Fig. S3B) will favor flow of C from 3PGA on into PEP and C_4 acids (for details, see Supplemental text, Section 3.1). Incidentally, synthesis of PEP form 3PGA will support continued activity of PEPC in the first seconds after the decrease in irradiance, even if PPDK is rapidly inactivated

Taken together, the overall decline of DHAP is well in excess of that required to cover the estimated shortfall in ATP and NADPH in the first 10 or 15 s after the shift to LL, and it is likely that much of this decline is due to oxidation of DHAP to 3PGA.

Metabolism of malate to might provide a further source of reducing equivalents after the shift to LL. As measurements of total malate do not provide information about whether there is a decline of the malate pool that is directly involved in the CCM, the rate of malate metabolism cannot be directly assessed. In principle, one possible route would be conversion of malate back to OAA by NADP-MDH, generating NADPH in the MC chloroplast. Any further conversion of OAA top aspartate would promote this reaction. However, there was no detectable increase in aspartate in the 10-15 s after the ML-LL switch (Fig. 3A). Another possible source of NADPH would be that, NADP-ME continues for a short time to convert malate to pyruvate and NADPH in the BSC at higher rates than in steady state LL. Laisk and Edwards (1998) argued that decarboxylation of malate by NADP-ME will be restricted after darkening because this reaction would rapidly reduce all the available NADP in the BSC chloroplast. After a switch to LL the situation may be different because NADPH consumption could continue for 3PGA reduction, using ATP produced by the BSC light reactions. That said, the downwards trend of pyruvate in the first 15 s after the decrease in irradiance (Fig. 3A) indicates flux decreases at NADP-ME very soon after the switch to LL. Further, there was a rise of fumarate (Supplementary Figs. S3A-B) which, although not significant, is consistent with a small increase rather than a decrease of the metabolically active pool of malate. Together, these observations indicate that the NADP-ME reaction may not be a major source of NADPH after the transition to LL. Incidentally, the decline of pyruvate is unlikely to be due to increased PPDK activity, because PEP, the product of the PPDK reaction, declined slightly by 10 s (Fig. 3A; Supplementary Fig. S3B) and because lower irradiance will anyway lead to a shortfall of ATP in the MC.

It is possible that some of the HCO_3 fixed into OAA by PEPC in the first seconds after the transition to LL accumulates as C_4 acids that are not decarboxylated and therefore do not require a large energy input in the CBC. Accumulation as malate is unlikely as this would consume one NADPH per C fixed. Rather, any shortfall of NADPH in the MC after the decrease of irradiance might lead to freshly synthesized OAA remaining as OAA or being converted to aspartate. However, as already mentioned, there was no increase in aspartate in the first 15 s after decreasing irradiance (Fig. 3A; Supplementary Fig. S3B), but as the aspartate pool is large it is possible that some C does accumulate in aspartate but this is masked by biological noise. This argues

against any major uncoupling of HCO_3 incorporation by PEPC and CO_2 assimilation in the CBC. Anyway, if there were any uncoupling, it would decrease the energy required to support A_n immediately after the transition to LL and further strengthen the argument that the observed decline in DHAP is large enough to supply the ATP and NADPH required for the observed buffering of net CO_2 assimilation.

- *Metabolic responses during the subsequent decline of photosynthesis to a trough: net decrease of the energy shuttle and CCM pools and changes in levels of CBC intermediates indicative of enzyme inactivation*

A second phase defined by the PC analysis started at about 15 s and lasted through to ~120 s (see Fig. 2A), corresponding to most of the gradual decline of A_n to a minimum, or trough, at about 250 s. As discussed in the main manuscript, a crucial factor in this phase was a delay in inhibition of end-product synthesis resulting in C being drained from the CBC and CCM pools. In addition, the changes of metabolites provide further insights into the topology of C_4 photosynthesis and how the CBC and CCM are regulated following a decrease in irradiance.

This decline in A_n was accompanied by an increase of FBP and SBP levels (Fig. 3A; Supplementary Figs. S3B, S7A). The increase of FBP was not accompanied by an increase of DHAP, as might be expected if the aldolase reaction were near to equilibrium. This could reflect the complex intercellular compartmentation of these metabolites in maize leaves. Most of the DHAP is located in the MC and most of the FBP in the CBC in the BSC (Leegood 1985; Stitt and Heldt 1985a; Arrivault et al. 2017). It is possible that the overall DHAP pool mainly reflects the DHAP pool in the MC, the overall FBP pool mainly reflects that in the CBC in the BSC chloroplasts, and that DHAP in the BSC rises in parallel with FBP. Indeed, as A_n falls, the size of the concentration gradient that is required to drive diffusion of DHAP from the MC to the BSC will decline, and this could include not just a decrease of the total DHAP pool (as seen in Fig. 3A), but also a relatively larger decline of the pool in the MC and maintenance or even an increase of the pool in the BSC,

The increase of FBP and SBP levels (see above), maintenance of F6P and decline of S7P (Fig. 3A; Supplementary Figs. S3B, S7A) and the resulting increase of both the FBP/F6P and SBP/S7P ratios (Fig. 3B; Supplementary Figs. S3B, S7B) point to inactivation of plastidic FBPase and SBPase. This may reflect falling availability of reducing equivalents. Doncaster et al (1989) reported that NADP-MDH activation, which is proxy for availability of reducing equivalents (Scheibe and Stitt 1988), decreased dramatically in the first minute after decreasing irradiance in maize.

There was an increase of RuBP levels (Figs. 3A, 5B; Supplementary Fig. S7A), the RuBP/3PGA ratio and, though less consistently, the RuBP/2PG ratio (Fig. 3B; Supplementary Figs. S3B, S7B). These responses are consistent with decreased activation of Rubisco, possibly reflecting restriction of Rubisco activase by a shortfall of ATP (Portis and Parry 2007; Portis et al. 2008). The 3PGA/DHAP ratio, which rose immediately after the drop in irradiance (see above), declined between 15-120 s (Fig. 3B; Supplementary Figs. S3B, S7B), as expected as falling A_n rebalances the relationship between provision of ATP and NADPH by the light reactions and their consumption in the CBC and CCM.

Overall, RuBP and many metabolites involved in its regeneration declined between 15 s and 120 s (Figs. 3A, 3C; Supplementary Figs. S3B, S7A, S7C). In this time span pyruvate decreased, but not PEP, alanine or aspartate (Fig. 3A; Supplementary Figs. S3B, S7B). Summed C in pyruvate and aspartate (Figs. 3C, 6B) and summed C in all CCM intermediates remained high for most of this phase (Fig. 3C; Supplementary Fig. S3B). Overall, the changes of CBC, energy shuttle and CCM metabolites were rather uncoordinated (for reasons see above, section 2, 'Rate of exchange of C between the CBC (including the energy shuttle metabolites) and the CCM'. The phase ended with a transient trough of A_n.

- *Metabolic responses during the partial recovery of photosynthesis: adjustments within the CBC and partial recovery of the energy shuttle and CCM pools*

The third phase captured by the PC analysis started by 300 s (Fig. 2A) and corresponded to a slow partial recovery of A_n that was equivalent to almost 8 % of the difference in the rate of photosynthesis in ML and LL, and continued until ~1200 s. This feature has been observed in other studies, including experiments where maize was transferred from 1700 to 144 µmol m^{-2} s^{-1}, Doncaster et al. (1989) and many but not all previous studies under fluctuating light (Lee et al. 2022; Arce-Cubas et al. 2023b, Sales et al. 2025, see also Discussion in main manuscript).

The partial recovery of A_n was accompanied by a decrease in FBP levels (Fig. 3A; Supplementary Figs. S3B, S7A) and a decrease in FBP/F6P ratio (Fig. 3B; Supplementary Figs. S3B, S7B), consistent with activation of FBPase and Rubisco. SBP levels (Fig. 3A; Supplementary Figs. S3B, S7A) and the SBP/S7P ratio (Fig. 3B; Supplementary Figs. S3B, S7B) rose, indicating SBPase is regulated independently to plastidic FBPase. There was an decrease of RuBP (Fig. 3A; Supplementary Figs. S3B, S7A) and the RuBP/3PGA ratio (Fig. 3B; Supplementary Figs. S3B, S7B), indicative either of increased activation of RuBP and/or an increase in the CO2 concentration in the BSC. Summed C in the CBC pool, excluding the energy shuttle intermediates, even declined slightly (Fig. 3C; Supplementary Figs. S3B, S7C).

During the partial recovery of A_n, the pools of the two energy shuttle intermediates, 3PGA and DHAP, increased (Figs. 3C, 6B; Supplementary Fig. S3B). There was also a marked rise in the levels of pyruvate, alanine and also aspartate (Figs. 3A, 6B; Supplementary Fig. S3B) resulting in a ~70% rise in the total C in CCM metabolites (Fig. 3C; Supplementary Figs. S3B, S7C). Most of these changes were significant when regressions were calculated across the 300, 600, 1200 and 1800 s time points (Supplementary Figs. S6C, S7A, S7C) or the 600, 1200 and 1800 s time points were individually tested against the 300 s time point (Figure S3C). Overall, the C that accumulated in the CBC, energy and CCM pools was equivalent to about 10% of all the CO_2 fixed during the recovery phase, and more in the first part of the recovery. As argued in the main manuscript, this provides evidence that the trough and partial recovery of A_n, is due to depletion and reestablishment of the pools in the energy shuttle and CCM

3.2 Supplementary to section '*Response of the CBC and CCM to an increase in irradiance'*

- *Metabolic responses in the first 5 sec before there is a significant increase of An*

A sudden increase in irradiance was followed, within 5 s, by a decrease of the 3PGA/DHAP ratio. This immediate response presumably reflects increased availability of ATP and NADPH from the light reactions. It occurred before A_n started to rise, at 5-6 s. Interestingly, the FBP/F6P and SBP/S7P ratios fell in the first 5-10 s after the increase in irradiance (Fig. 4B; Supplementary Fig. 4B; the decrease of the FBP/F6P ratio was significant and the decrease of both ratios was consistent across experiments, see Supplementary Fig. S5D). This points to rapid activation of FBPase and SBPase after an increase in irradiance. The FBP/F6P and SBP/S7P ratio did not increase at 10 or 30 s after a switch to high light in a 300 s fluctuating regime (Sales et al. 2025), possibly because the enzymes were not fully inactivated in the shorter time in LL. As in the ML-LL transient, DHAP changed in an opposite manner to FBP, possibly reflecting the intercellular location of these metabolites (see previous subsection).

The initial decline of 3PGA at 5 s was accompanied by a larger and significant decrease in PEP (Fig. 3A; Supplementary Fig. S3B). A similar decrease was seen 10 s after a shift to high light in a 300 s fluctuating regime (Sales et al. 2025). This rapid and overproportioned decrease of PEP is unlikely to be solely due to the decrease in 3PGA (see also Section 2, above). Furthermore, pyruvate showed a small but significant increase at 5 s (Fig 3A; Supplementary Fig. S3B, resulting in a 2-fold increase in the pyruvate/PEP ratio (not plotted). Pyruvate also rose at 10 s after shifting to high light in a 300 s fluctuating regime (Sales et al. 2025). This points to a temporary restriction on flux at PPDK. As shown by Chen et al (2014), PPDK is post-translationally regulated not only in response to dark-light switches, but also in response to changes in irradiance intensity. It is possible that the resulting decline in PEP temporarily restricts flux at PEPC, especially as this enzyme is also not fully activated and the inactive form has lower affinity for PEP, decreased sensitivity to activation by activating metabolites, and higher lower sensitivity to inhibition by inhibitory metabolites (Ashton et al.1990; Doncaster and Leegood 1987; Vidal et al. 2002)

The subsequent increase of A_n was spread over the next 10-15 min, with a rapid rise in the first 90 s (interrupted by a short plateau at ~10-15 s) and then a slower rise from 90 s onwards (Fig. 1B; Supplementary Figs. 1F-G). A similar complex response was seen in a 300 s fluctuating regime (Sales et al. 2025), although the later slow rise was of course absent.

- *Metabolic responses accompanying the rise of A_n until 90s, including factors that might underly the plateau of A_n at 9-15 s*

The rise of A_n from 6 s until about 90 s (Fig. 2B; Supplementary Fig. S1G) corresponded broadly to phase 2 of the PC analysis (Fig. 2B). Overall, the rise of A_n was associated with a rapid increase in CBC metabolite pools and enzyme regulation. There was a coordinated increase in 3PGA, DHAP, FBP, SBP, S7P, pentose-P and RuBP (Fig. 4A; Supplementary Figs. S4B, S8A). From about 10 s onwards, there was a decrease in the pentose-P/RuBP ratio (Fig. 4B; Supplementary Figs. S4B, S8B), pointing to rapid activation of PRK. After a drop at 5-10 s, the FBP/F6P ratio rose until 30 s and then fell (Fig. 4B; Supplementary Figs. S4B, S8B) pointing to slightly delayed activation of FBPase. The RuBP/3PGA and RuBP/2PG ratios rose (Fig. 4B; Supplementary Figs. S4B, S8B), suggesting that Rubisco is becoming increasingly restrictive for CBC flux.

This rise of A_n was interrupted, slowing down by about 9 s and plateauing between about 11-15 s before starting to rise again (Fig. 2B; Supplementary Fig. S1G). A similar interruption was observed by Lee et al. (2022) after the shift to high light 120 s step fluctuating regime with 2 min for three C_4 species but not maize, and by Sales et al. (2025) for maize in fluctuating light with 300 s steps at 25°C, where the interruption lasted until almost 30 s. Indeed, in fluctuating regime with 30 s light steps, the interruption lasted until the end of the high light period (Sales et al. 2025).

In our LL-ML shift, the transient interruption between ~11-15 s was not accompanied by marked changes of metabolite levels, except for the preceding decrease in PEP and increase of the pyruvate/PEP ratio (see above), a decrease of the FBP/F6P ratio, a small increase of DHAP and pentose-P and a marked increase in ADPG (Fig. 4A; Supplementary Fig. S4B). Indeed, the 10 and 15 s time points, close to the start and end of the plateau, lay close to each other in the PC analysis (Fig. 2B).

Nevertheless, the metabolite data do allow exploration of some potential explanations for this transient interruption in the rise of A_n. These include i) a transient block on delivery of CO_2 by the CCM, and the need to increase this via changes in CCM intermediate pools and/or post-translational activation of enzyme or ii) a transient block on CO_2 assimilation in the CBC resulting in an increase of C_{BSC} and increased back-leakage of CO_2, and the need to increase CBC flux by building up CBC pools and/or activating CBC enzymes (see Introduction and above).

The decrease of PEP at 5-10 s is consistent with the idea that there is a shortfall in CO_2 pumping due to a delay in PPDK activation and restriction of PEPC by low PEP. This is, however, unlikely to be the main, let alone only, factor leading to the plateau. RuBP levels remained unchanged (Fig. 4A; Supplementary Fig. S4B) rather than rising as would be expected if there was a major shortfall of CO_2 in the BSC. The implication is that there is also a restriction on RuBP regeneration rather than use of RuBP for the carboxylation reaction. The FBP/F6P and SBP/S7P ratios rose from 10 s onwards, but the increase continued for the FBP/F6P ratio unit 30 s long after the end of the plateau, and the rise of the SBP/S7P ratio was not significant. As a side note, the rise in ADPG (Fig. 4A; Supplementary Fig. S4B) might point to a loss of poise between CBC metabolites and Pi due, for example, to slow activation of sucrose synthesis, with falling plastid Pi levels allosterically activating ADPG pyrophosphorylase (Ballicora et al. 2004). However, ADPGlc remained high at 30, 60 and 120 s, long after the plateau when A_n was rapidly increasing, making it unlikely that the reason for the plateau in A_n is directly linked to high ADPGlc (see below for an alternative explanation for the increase of ADPGlc). Altogether, these observations indicate that the plateau is not primarily due to a short-fall in CO_2-pumping by the CCM.

An alternative explanation for the plateau would be rapid back-leakage of CO_2 from the BSC to the MC, due to decarboxylation of C_4 acids transiently exceeding the rate of CO_2 assimilation by the CBC. Bursts of CO_2 release have been reported in C_4 species including maize following reillumination after a short time in darkness (Krall and Pearcy 1993; Laisk and Edwards 1998). In a recent study under fluctuating light, Lee et al. (2022) discussed the transient interruption of CO_2 uptake in terms of a CO_2 burst. In a study with fluctuating light, Sales et al (2025) observed a large increase in the number of electrons transferred per CO_2 fixed

immediately after an increase in light intensity. This was evident in a regime with 300 s steps between low and high light, and even more evident in regimes with faster fluctuations. This observation points to a loss of coupling between linear electron transport and CO_2 assimilation, and is consistent with over-pumping and back-leakage of CO_2.

Some aspects of our metabolite data are consistent with this idea, especially when interpreted in terms of possible differing responses of metabolites in the BSC and MC. In the first 15 s in ML, as already mentioned, 3PGA declined, there was a small non-significant increase in DHAP and an opposing upwards trend for FBP and SBP, as well as the FBP/F6P and SBP/S7P ratios (significant for the FBP/F6P ratio). The opposing changes of DHAP and FBP might be explained as follows: whilst in the MC the increased light intensity may immediately promote reduction of 3PGA to DHAP, this may not be the case for BSC where reduction of 3PGA to DHAP may be limited by availability of NADPH. As there is little PSII activity in the BSC, availability of NADPH is dependent on flux at NADP-ME and on an operational energy shuttle. Both may not be operating effectively in the first seconds after a switch to high irradiance (see later in the paragraph for discussion of how long it takes until the energy shuttle speeds up). Incidentally, a transient restriction on 3PGA reduction in the BSC chloroplast might explain the marked and significant rise of ADPG at 5-15 s (Fig 4A, Supplementary Fig. 4B). At the same time, the rate of carboxylation of RuBP to produce 3PGA may be restricted. On the one hand, low DHAP, FBP, SBP (Fig. 4A) and delayed activation of FBPase (as revealed by the significant increase in the RBP/Fru6P ratio, Fig. 4B, Supplementary Fig. 4B) may restrict RuBP regeneration: in agreement, RuBP levels were initially unaltered and only rose after 15 s (Fig. 4A, Supplementary Fig. 4B). Delayed activation of Rubisco may additionally contribute to a short fall in CO_2 assimilation and production of 3PGA in the CBC. Crucially, the marked increase in the overall pools of DHAP and 3PGA does not start until about 30 s after the shift to ML, indeed at 5-15 s the overall 3PGA pool is lower than in LL (Fig. 4A, 4C, Supplementary Fig. 4B). This delayed increase of the 3PGA and DHAP pools will restrict the size of their intercellular concentration gradients and, hence, operation of the energy shuttle that is required to compensate for the inherent NADPH deficit in the BSC. These factors could all contribute to restrict CO_2 assimilation in the BSC by the CBC, leading to leakage of CO_2 back to the MC and, if it is not all refixed by PEPC, to release of CO_2 from the leaf. Our metabolite data are indeed consistent with a temporary restriction on PEPC activity; for example, PEP decreases by up to 40% in the first 10 s in ML. This decline might partly reflect a restriction on flux at PPDK, as indicated by the small but significant rise in pyruvate at 5-15 s. However, the decline in PEP is probably buffered by net conversion of 3PGA to PEP, driven the by increased 3PGA/PEP ratio at 10-15 s (significant at 10 s, see Supplementary Text, Section 2.2)

One other aspect that might be noted is that the initial significant increase of 2PG at 5 s was reversed and that the RuBP/2PG ratio declined by 10 s; these are only trends but are consistent with the idea that an initial shortfall of CO_2 in the BSC 5 s after the shift to ML (see Supplementary Test Section 6 below) may be reversed by 10-15 s, possibly due to transient accumulation of CO_2 that is delivered by the CCM but is not yet being rapidly assimilated in the CBC

Overall, it is likely that multiple factors contribute to the transient interruption of the rise of A_n, partly masking the fingerprint of individual factors. It is also likely that leaf-to-leaf variance complicates the comparison of A_n

and metabolite levels in this short transient. There was considerable leaf-to-leaf variation in A_n during the plateau (Fig. 1B) due to different values of A_n in the plateau and to the plateau starting and ending at slightly different times (Supplementary Fig. S1M). This is presumably accompanied by leaf-to-leaf variation in metabolite levels. Further, the half time of photosynthetic intermediates, especially RuBP and those involved in RuBP regeneration in the CBC as well as PEP are of the order of one or a few seconds (Stitt et al. 1980; Arrivault et al. 2009). Probably, a better understanding of the reasons for the transient interruption would be aided by denser time sampling of leaf material in which A_n is being simultaneously monitored. That said, the most plausible explanation for the transient interruption in the rise of A_n is that there is a delay in the rise in flux in the CCM and in the rise in flux in the CBC, and that fluxes in the two cycles become transiently imbalanced after a transition from LL to ML. The extent of the one or the other factor will probably vary, depending on prehistory and perturbation.

Following the plateau, A_n rose rapidly until about 90 s. This rise is partly linked with an increase in CBC pools (see main Manuscript and beginning of this subsection). However, enzyme activation may also contribute. In a theoretical study, Wang et al. (2021) predicted that activation of Rubisco activase and the PPDK regulatory protein restrict photosynthesis after a dark to high light transition. Our analyses of metabolites provide experimental support for the idea that slow Rubisco activation may restrict A_n in the first phase of the response after a switch from low to moderate irradiance. RuBP increased until 30 s and then s was at fairly constant levels despite the further large rise in A_n (see Figs. 4A, 5C; Supplementary Fig. S8A). In earlier studies of a dark to high light transition, RuBP rose to a peak at 2 min before declining as A_n rose further (Usuda 1985), again pointing to delayed activation of Rubisco. Our data also support the idea that the rise of A_n is initially restricted by slow activation of PPDK. The marked decrease of PEP and slight but significant increase of pyruvate at 5-15 s after a sudden increase in irradiance (Figs. 4A, 5B; Supplementary Fig. S4B), points to consumption of PEP by PEPC outstripping delivery of PEP by PPDK. A decrease of PEP and increase of pyruvate was also seen 10 s after switching to high light in a 300 s frequency fluctuating regime (Sales et al. 2025). In earlier studies of a dark to high light transition, pyruvate initially declined, before starting to rise from about 2 min on (Furbank and Leegood 1984; Usuda 1985). This initial drop may reflect the difference between a transition starting from darkness and from low light, when PEPC and NADP-ME may already be slightly activated. The subsequent rise of pyruvate in these earlier studies started at about the same time as in our LL-ML transition.

- *Metabolic responses accompanying the gradual increase from 90 s onwards*

From about 90 s onwards, A_n continued to rise, although more slowly (Fig. 2B; Supplementary Fig. S1G). A similar biphasic response was observed in maize and several other NADP-ME subtypes (*Setatia viridis*, *Setaria italica*, *Sorghum bicolor, Andropogon gerardii* Vitma, *Miscanthus × giganteus*, *Saccharum spp*., *Purus frumentum*) as well as NAD-ME species (*Amaranthus mangostanus*, *Panicum virgatum* L.) under a fluctuating light regime with a high light phase of 120 s or longer by Li et al. (2022) and Lee et al. (2023). In a detailed study of maize in fluctuating light, Sales et al. (2025) observed a similar two-phase response of A_n in a 300 s fluctuating regime after switching to high light, and that in a 60 s fluctuating regime A_n only a rose to a value resembling the shoulder of the response (see above). On the other hand, a clear biphasic response was not

observed in the PEPCK species *Alloteropsis semialata* (Arce-Cubas et al. 2023b) and *Spartina pectinata* L (Lee et al. 2023). Thus, with a possible exception of PEPCK subtypes, this two-phase response to an increase in irradiance may be a general feature of C_4 photosynthesis and occurs both in single transitions and fluctuating light. It represents a second reason for loss of photosynthetic C gain after an increase in light intensity.

In the maize LL-ML transition, the gradual rose of A_n broadly corresponded to the third phase defined in the PC analysis (Fig. 2B). FBP fell and SBP, S7P (except at the very last times), pentose-P and RuBP remained unchanged (Figs. 4A, 5C; Supplementary Figs. S4B, S8A). The FBP/F6P ratio declined, the SBP/pentose-P ratio was fairly constant, the pentose-P/RuBP ratio rose, and the RuBP/3PGA and RuBP/2PG ratios did not show any consistent changes, pointing to small adjustments of poise within the CBC (Fig. 4B; Supplementary Fig. S4B). The most striking changes in this phase were a continuation of the rise of 3PGA and DHAP levels (Fig. 4A), and an increase in the levels of PEP, pyruvate and alanine (Fig. 4A; Supplementary Figs. S4B, S8A). This resulted in a marked increase in the summed pools involved in the energy shuttle and in the CCM (Figs. 4C, 6C; Supplementary Figs. S4B, S8C).

The gradual rise in A_n correlated strongly with the increase in the pool size of the CBC, energy shuttle and CCM (Figs. 6A, 6C; Supplementary Figs. S4B, S8C). The CBC contributed in the first part of the transient mostly as A_n rose from 50 to about 90 nmol CO_2 g^{-1} FW s^{-1}, and the energy shuttle and CCM metabolites in the second part as A_n rose from 90 to 130 nmol CO_2 g^{-1} FW s^{-1}. This provides strong correlative evidence that the second and slower part of the rise in A_n closely linked with the gradual build-up of the large metabolites pools that drive these intercellular shuttles. Overall, the rise in A_n was accompanied by an increase of summed C in the CBC, energy shuttle and CCM of about 7000 nmol C g^{-1} FW, which is equivalent to about 55 s of A_n in ML, and considerably more of the lower A_n that prevailed for the first part of the ML-LL transition.

4. Contribution of different decarboxylation routes

Although C_4 plants are often subdivided into NADP-ME, NAD-ME and PEPCK sub-types based on their main route for decarboxylation, in many species including maize different decarboxylases operate concomitantly (Furbank 2011; Bräutigam et al. 2014; Wang et al. 2014a). Maize has substantial expression (Furomoto et al. 1999; Pick et al, 2011; Wang et al. 2014c) and activity of PEPCK in the BSC (Walker et al. 1997). In this, maize resembles other NADP-ME subtypes. In maize bundle sheath preparations, decarboxylation of malate occurred via NADP-ME leading to formation of pyruvate, and decarboxylation of aspartate occurred via PEPCK leading to formation of alanine (Wingler et al. 1999)., Maize bundle sheath preparations also decarboxylate aspartate via an NAD-ME reaction (Chapman and Hatch, 1981). The proportion of CO_2 carried by aspartate-based routes was estimated from ^{14}C labeling as about 12% in maize leaves (Hatch 1971) and maize bundle sheath preparations (Chapman and Hatch 1981), as ~10% from analysis of $^{13}CO_2$ labelling kinetics in moderate light in wild-type maize leaves (Arrivault et al., 2017) and as high as 25% from a comparison of wild-type maize and the *dct2* mutant leaves (Weissmann et al. 2016).

The contribution of different decarboxylation routes may depend on irradiance. Analyses of steady state metabolite levels in maize in steady state low irradiance and high irradiance revealed with higher levels of aspartate and alanine relative to pyruvate under low irradiance, pointing to an increased contribution of PEPCK at low irradiance (Usuda 1987; Leegood and von Caemmerer 1989). Aspartate increased in maize leaves of maize and other C_4 species during adjustment to a large sudden decrease in irradiance (Doncaster et al. 1989). These observations point to an increased contribution of aspartate-based decarboxylation in low irradiance. A similar conclusion was reached from $^{13}CO_2$ labelling studies in maize at low and moderate irradiance, with substantial labelling of aspartate at low irradiance and lower labelling at moderate irradiance, whilst malate showed an opposite pattern (Medeiros et al. (2022). Expression and enzymatic activity of NADP-ME and PEPCK as well as malate and aspartate pool sizes and labelling kinetic vary depending on canopy position in maize, with higher NADP-ME activity and estimated flux relative to that of PEPCK in upper leaves and an increased PEPCK contribution in subtending leaves (Arp et al., 2021). This may reflect the more heterogenous light environment in lower leaves and a possible role for PEPCK decarboxylation route in coping with light fluctuations.

In our current study of irradiance transitions, aspartate showed a strikingly different pattern to most other metabolites (Fig. 5; Supplementary Figs. S6A-B). Aspartate levels rose significantly during the adjustment to low irradiance (ML-LL, Fig. 3A; Supplementary Figs. S3B, Fig. S7A), whereas most other metabolites declined. Aspartate levels decreased significantly during the adjustment to higher irradiance (LL-ML, Fig. 4A; Supplementary Figs. S4B, S8A), whereas most other metabolites increased. In both cases, the change in aspartate levels occurred in the later part of the response, from ~120 s onwards. Similar slow changes occurred between the start and end of the light phases in a 300 s fluctuating light regime (Sales et al. 2025). These observations indicate that the contribution of decarboxylation routes other than NADP-ME may increase in the later part of the adjustment to lower irradiance, and may decrease in the later part of the adjustment to higher irradiance.

One possible explanation for the increase of aspartate in low light is that any shortfall of NADPH in the MC would, via mass action, restrict conversion of OAA to malate, and favor conversion of OAA via aminotransferase to aspartate. A reciprocal scenario might occur after an increase in irradiance. Such effects would be expected to occur immediately after the change in irradiance. However, the changes of aspartate level occurred relatively slowly during the transitions (Figs. 3A, 4A, see also Sales et al. 2025). The slow response indicates either that redox-driven changes in metabolite levels do not play a large role, or that conversion of OAA to aspartate also depends on other factors, which change more slowly.

Another explanation may be post-translational regulation of NADP-MDH. Activation and inactivation of NADP-MDH requires up to 10 min after illuminating or darkening maize chloroplasts (Rebeille et al. 1986; Ashton et al. 1990). If the changes in activation and deactivation are similarly slow in maize leaves after changes in irradiance, this might contribute to the delayed response in the levels of aspartate in the ML-LL and LL-ML transitions (see also below for further discussion) although probably being less relevant in fluctuating light.

A further explanation might be the availability of amino donors. This explanation is supported by the observation that the 2OG/glutamate ratio and the pyruvate/alanine ratio were high in moderate irradiance and remained high in the first part of the adjustment to low irradiance and that both ratios were high in low irradiance and declined gradually after transfer to higher irradiance (Figs. 3B, 4B). Slow changes of alanine, glutamate and 2OG were also seen in 300 s fluctuating light (Sales et al. 2025).

To maintain nitrogen stoichiometry, movement of aspartate from the MC to the BSC must be coupled to movement of an amino acid back from the BSC to the MC. In the most parsimonious pathway, this would involve movement of alanine (Weissmann et al. 2016; Bräutigam et al. 2018). In the ML-LL transition the rise of aspartate was accompanied by a significant decrease of the pyruvate/alanine ratio (Fig. 3B; Supplementary Figs. S3B, S7B) mainly due to the decrease of pyruvate being larger than the decrease of alanine (Fig. 3A). In the LL-ML transition, the decline of aspartate was accompanied by a significant increase in the pyruvate/alanine ratio (Fig. 4B; Supplementary Figs. S4B, S8B), in this case mainly due to the increase of pyruvate being larger than the increase of alanine (Fig. 4A). In both irradiance transitions, for most of the time the levels of aspartate and alanine were correlated negatively rather than positively (see Fig. 3A, 4A, 5A; Supplementary Figs. S6A, S6B). Whilst there was a positive correlation between aspartate and alanine in the last part of the transition to low light, this was also accompanied by a rise in pyruvate and might just reflect a general increase in the pool of 3C metabolites (Fig. 3A, see also Discussion section 'Response of the CBC and CCM to a decrease in irradiance' and Supplementary Text Section 2.1). In studies of light-dark transitions in maize leaves, Furbank and Leegood (1984) reported that aspartate and alanine levels decline in the first 5 min after illumination, and Usuda (1985) reported that aspartate transiently declined and recovered whereas alanine transiently rose and then decreased. Taken together, these observations argue against operation of a tight obligatory intercellular shuttle between aspartate and alanine, as this would require parallel changes of both metabolite pools to drive a coordinated change in the intercellular movement of both metabolites.

An alternative scenario would be that some of the amino groups from aspartate return from the BSC to the MC as another amino acid than alanine. One possibility is that glutamate and 2OG might provide a secondary shuttle to maintain nitrogen stoichiometry (Mallmann et al. 2024; Medeiros et al. 2022). This idea is supported by the changes of metabolite profile during our light transitions.

It was striking that the levels of 2OG and glutamate were high and changed markedly in the ML-LL transition and LL-ML transition. During adjustment to low light, the 2OG/glutamate ratio decreased significantly (Fig. 3B) due to an upwards trend of glutamate and downwards trend of 2OG (Supplementary Fig S3A). Aspartate rose (Fig. 3A), correlating positively with glutamate (see Fig. 3A, Supplementary Fig. S6) and negatively with the 2OG/glutamate ratio (compare Fig 3A and Fig. 3C). During adjustment to high light, the 2OG/glutamate ratio increased significantly (Fig. 4B) due to a significant increase in 2OG (Supplementary Fig S3A). Aspartate declined (Fig. 4A), independently of glutamate but reciprocally to the 2OG/glutamate ratio (compare Fig 4A and 4B). As already mentioned, aspartate and alanine levels changed independently and often reciprocally in our ML-LL and LL-ML transitions. In their study of 300 s fluctuating light regime, Sales et al. (2025) observed similar changes of the aspartate, alanine, pyruvate, glutamate and 2OG. These observations suggest that whilst some of the amino groups carried by movement of aspartate from the BSC to the MC may

be returned by movement of alanine from the BSC to the MC, some are returned by a secondary shuttle involving glutamate and OG. In relative terms, the glutamate/2OG shuttle may make an especially large contribution in low irradiance, when the contribution of aspartate-based routes like PEPCK increases.

In the BSC, aspartate/2OG aminotransferase is located the chloroplasts, alanine/2OG and pyruvate/glutamate aminotransferase in the mitochondria, and aspartate/2OG aminotransferase in the cytosol (see e.g., Hatch and Mau 1973, Chapman and Hatch 1981; Orzechowski et al. 1999). In the MC, the largest part of the aspartate/2OG and pyruvate/glutamate aminotransferase activity is located in the chloroplast (Slack and Hatch 1969) (see also Majeran et al., 2010 for transcriptome data).

A contribution of secondary shuttles involving glutamate and 2OG was already proposed for metabolic transformations within the BSC in non NADP-ME species by Hatch and Mau (1973), based on labelling experiments in BSC preparations where provision of 2OG promoted aspartate decarboxylation. Chapman and Hatch (1981) made similar observations in studies of the ancillary aspartate-based decarboxylation route in BSC preparations from the NADP-ME species maize. Weissmann et al. 2016), in their study of the *dct2-1* maize mutant (deficient in the BSC chloroplast DICARBOXYLIC ACID TRANSPORTER2, which is required to import malate), proposed that the blocked malate import might be circumvented by routes involving aminotransferase reactions with 2OG and glutamate, albeit at very low rates. On the one hand, aspartate might be taken up into the chloroplast, converted by 2OG/aspartate aminotransferase to OAA that is then reduced to malate and decarboxylated by NADP-ME, with the resulting pyruvate then being converted by pyruvate/alanine aminotransferase to alanine before exiting the chloroplasts; this route might be upregulated in the malate transport-compromised *dct-2* mutant. A second route would be 2OG/aspartate aminotransferase-dependent conversion of aspartate in the cytosol or mitochondria to generate OAA for PEPCK. Such intracellular shuttles might also provide a possible explanation why aspartate and glutamate promote decarboxylation of malate in main bundle sheath preparations (see e.g., Boag and Jenkins 1986).

Our analyses of light transitions show that in addition to providing flexibility in the BSC, a intercellular glutamate/2OG shuttle contribute to maintenance of N stoichiometry between the MC and BSC. Secondary nitrogen shuttles might provide added flexibility by avoiding restrictions that a shortfall of one single metabolite might place on the rate of intercellular movement and thence the rate of photosynthesis, and by decreasing the required pool size for any individual metabolite (in analogy to the arguments why multiple decarboxylation route operate in parallel, see Furbank 2011; Wang et al. 2014a; Bellasio and Griffiths 2014a).

Interestingly, the levels of 2OG and glutamate increased during the evolution of C_4 photosynthesis in the *Flaveria* genus was that (Borghi et al. 2019; Tang et al. 2023). Although often classified as NADP-ME subtype, these species have an especially high contribution of PEPCK to decarboxylation (Meister et al. 1996). The most likely explanation for the increase of 2OG and glutamate is that, as discussed above, secondary shuttles involving 2OG and glutamate contribute to malate/aspartate and pyruvate/alanine interconversions within a given cell type, as well as to the maintenance of nitrogen stoichiometry between the MC ad BSC. Other potential explanations for the increase in 2OG and glutamate in C_4 *Flaveria* species would include a link to changed function of the GOGAT pathway. This seems unlikely, however, given that

the rate of photorespiration and hence of recycling of photorespiratory NH_4 is lower in C_4 than C_3 photosynthesis. This will greatly decrease total flux through the GOGAT pathway and would be expected to be accompanied by a decrease of 2OG and glutamate. Another possible explanation would be that the changes of 2OG and glutamate during the progression for C_3 to C_4 photosynthesis is unrelated to C_4 photosynthesis *per se*. This cannot be excluded but seems unlikely in view of the marked changes of 2OG and glutamate during light transitions in maize.

The question also arises, why contribution of aspartate-based routes like PEPCK to decarboxylation increases in low irradiance, both in steady state and during a transition to low light. Several factors might contribute. On the one hand, operation of the NADP-ME route may be constrained in low light (see also Medeiros et al. 2022). This could be for several reasons. In the MC they may include any restriction on conversion of OAA to malate due to low availability of NADPH or incomplete activation of NADP-MDH. Post-translational activation of NADP-MDH by thioredoxin is favored by a rising NADPH/NADP ratio as the light intensity rises in C_3 plants (Scheibe and Stitt 1988; Scheibe 1990; Knuesting and Scheibe 2018) and C_4 plants (Ashton and Hatch 1983; Rebeille et al. 1986; Ashton et al. 1990). As a result, high rates of OAA reduction in C_4 plants can only occur at high NADPH/NADP ratios (Ashton and Hatch 1983; Rebeille and Hatch 1986; Ashton et al. 1990). Incidentally, it was shown recently using Arabidopsis lines expressing constitutively active NADP-MDH that inactivation of NADP-MDH in low irradiance is important for photosynthetic performance of C_3 plants under fluctuating light (Yokochi et al. 2021). By extrapolation, inactivation and activation of NADP-MDH may also be important during light transitions in C_4 photosynthesis. Irrespective of the reasons, any restriction on synthesis of malate in low irradiance will presumably hinder the generation of a high concentration of malate in the MC, slowing down diffusion of malate to the BSC. In the BSC, several factors may restrict decarboxylation by NADP-ME in low irradiance, including a lack of demand for the products of NADP-ME (Hatch and Kagawa 1976; Bräutigam et al. 2018), incomplete light-activation of NADP-ME (Bovdilova et al. 2019) and/or poor transport of pyruvate back to the MC (see Medeiros et al. 2022 for discussion). On the other hand, operation of the PEPCK route may be favored at low irradiance compared to high irradiance. Maize PEPCK is not subject to post-translational light activation and is active in the dark (Wingler et al. 2019; Walker et al. 2002) and is presumably as active under low irradiance as in high irradiance. Indeed, moderate to high light may even lead to phosphorylation and inactivation of maize PEPCK (Chao et al. 2014).

A further factor that may allow a larger contribution of PEPCK under low irradiance relates to the route by which the PEP produced by PEPCK in the BSC is used to regenerate PEP in the MC, where it is needed to act as a substrate for PEPC. It is an open question how this occurs in PEPCK subtypes (Braütigam et al. 2018) and analogous issues arise when PEPCK is operating in parallel with for example, NADP-ME. The most parsimonious route would be via movement of PEP itself. However, this will be restricted by the relatively small size of the PEP pool (see Figs 3A, 4A), which restricts the size of the intercellular concentration gradient that can be generated to drive diffusion of PEP from the BSC to the MC. Further, movement by this route will also be constrained by the need to maintain high enough PEP levels in the MC to support rapid flux at PEPC. The relatively low concentration of PEP reflects the thermodynamics of the enolase and phosphoglycerate

mutase reactions, which favor formation of 3PGA and result, at equilibrium, in an approximately 3-fold excess of 3PGA over PEP (see also Supplementary Text, Section 2 above). In principle, a thermodynamically more favorable route to return PEP to the MC would be to convert PEP to 3PGA in the BSC, followed by diffusion of 3PGA down its larger concentration gradient to the MC and conversion of 3PGA back to PEP in the MC. However, this route would require activities of enolase and phosphoglycerate mutase that are well in excess of flux at PEPCK in order to maintain near-equilibrium concentrations of PEP and 3PGA in the BSC and in the MC. As discussed in above (Supplementary Text, Section 2), flux between 3PGA and PEP in maize is high enough to allow gradual rebalancing of pool sizes in the CBC and CCM, with about 10% of the C in these pools being able to move in about half a minute. It is however, not commensurate with carrying a net flux that exceeds flux at PEPCK, or even a fraction of this in a scenario like that in maize, where PEPCK makes a small contribution to the CCM. Further this route would require enolase and enolase and phosphoglycerate mutase in both the BSC and the MC, rather than an asymmetric distribution with the majority in the MC as is the case in maize (Furbank and Leegood 1984). Incidentally, in a study of the PEPCK subtype *Spartina anglica,* Hubb, Smith and Woolhouse (1983) concluded that the activities or enolase and phosphoglycerate mutase were insufficient to support interconversion of PEP and 3PGA at the required rate in either the BSC or the MC. Thus, a relatively low concentration of PEP and a low capacity for interconversion of PEP and 3PGA probably place an upper limit on flux that can be supported by a PEPCK carboxylation cycle in high light (see also Medeiros et al. 2022 for discussion). Under low irradiation, they could support a considerably larger contribution of PEPCK to the CCM.

5. Perturbation and adjustment of C_{BSC}

As discussed in the main text, changes in irradiance might result in a temporary imbalance between CO_2 concentration by the CCM and CO_2 utilization by Rubisco (Furbank et al. 1990; von Caemmerer 2000; Kromdijk et al. 2014). An excess of CO_2 influx over CO_2 consumption will lead to an increase in C_{BSC} and might increase back-leakage of CO_2 to the MC whilst an excess of CO_2 consumption over CO_2 influx will lead to a decrease in C_{BSC} and an increase in the rate of RuBP oxygenation relative to RuBP carboxylation. Both increased photorespiration and increased CO_2 back-leakage will decrease photosynthetic efficiency and could contribute to loss of photosynthetic efficiency after a change in irradiance.

Measurements of the rate of photorespiration and of C_{BSC} are complicated and challenging (Ubierna et al. 2011; Sage 2014; Kromdijk et al. 2014), especially in non-steady state conditions. Kubacek et al. (2013) reported that growth in fluctuating light led to increased back-leakage but could not determine in which part(s) of the fluctuating regime this occurred. Recently Wang et al. (2022) used a tunable diode laser absorption spectroscope to monitor delta^{13}C in combination with gas exchange to track back-leakage during a dark-light transition in maize and sorghum. For technical reasons, the measurements were performed at about 800 ppm CO_2 After illuminating maize, estimated leakiness increased progressively to a maximum at about 150 s before declining 2-fold to a steady state value over the next 1200 s. A slower increase in leakiness followed by a slow decline were reported for maize. This was qualitatively consistent with earlier modelling of leakiness during induction (Wang et al. 2021). However, whilst the time resolution was about 10 s, the error associated

with determination of delta^{13}C was >50% in the first 100 s after illumination indicating that the measurements in this time may have been masked by instrument error. Sales et al. (2025) investigated the relation between photosystem (PS) II quantum yield and instantaneous quantum yield of CO_2 assimilation, a measure of the degree of coupling between linear electron transport and CO_2 assimilation, in maize in fluctuating light. They found that the ratio was substantially increased in the first seconds after a switch to high light, consistent with light energy is being used to pump CO_2 that is not being assimilated but is instead leaking back to the atmosphere.

We asked whether our metabolite data might allow detection of changes in C_{BSC} with shorter time resolution. Measurements of 2PG provide a qualitative proxy for the rate of RuBP oxygenation, and the relationship between the RuBP/2PG ratio and the RuBP/3PGA ratio provides a qualitative proxy for the relative rates of RuBP oxygenation and RuBP carboxylation. Thus, these metabolic traits provide proxy information about C_{BSC}. However, it should be stressed that they do not provide unambiguous information because the levels of these metabolites are also affected by other events. The level of 2PG will also depend on how quickly it is degraded by 2-phosphoglycolate phosphatase, and the level of 3PGA (and hence the RuBP/3PGA ratio) will also depend on the rate of 3PGA reduction, which in turn depends on the level of triose-P and on the availability of ATP and NADPH.

Immediately following a decrease in irradiance, there was a rapid and significant >2-fold decrease in the level of 2PG (Fig. 3A; Supplementary Fig. S3B), whilst the RuBP/3PGA ratio declined and the RuBP/2PG ratio rose. Conversely, following an increase in irradiance, there was a significant increase of 2PG (Fig. 4A; Supplementary Fig. S4B), and a non-significant increase of the RuBP/3PGA ratio and decline of the RuBP/2PG ratio (Fig. 4B; Supplementary Fig. S4B). Similar changes in 2PG levels were observed in a study with fluctuating light (Sales et al. 2025), in which 2PG increased 30 s after shifting to high light, and decreased 10 and 30 s after shifting to low light

These rapid responses of 2PG point to an increase in carboxylation relative to oxygenation immediately after a decrease in irradiance, and a transient decrease in carboxylation relative to oxygenation immediately after an increase in irradiance. The former may transiently enhance and the latter transiently decrease photosynthetic efficiency, although the impact of the latter may be small due to the relatively low rate of oxygenation relative to carboxylation in C_4 photosynthesis.

The observed significant changes in 2PG and opposed trends of the RuBP and RuBP/2PG ratios are also indicative of an increase of C_{BSC} after a decrease in irradiance, and an increase of C_{BSC} after a decrease in irradiance. This would result in transiently increased and decreased back-leakage of CO_2, respectively.

Back-leakage cannot be directly measured but is instead modelled in various ways, each involving several assumptions (Ubierna et al. 2011; Sage 2014; Kromdijk et al. 2014). Estimated values of back-leakage (expressed as phi) are usually in the range of 0.2-0.3 of the C fixed by PEPC (Hatch et al.1995; Kromdijk et al. 2014; Wang et al. 2024) but depend on the conditions, for example, may increase in low irradiance (Evans et al. 1986; Kromdijk et al. 2014), and can be as high as 0.6-0.9 after a sudden decrease in irradiance (Cousins et al. 2006, 2008; Tazoe et al. 2006, 2008; Kromdijk et al. 2008, 2010, 2014; Pengelly et al. 2010).

In a recent analysis of $^{13}CO_2$ labelling patterns in stable low irradiance, Medeiros et al. (2022) estimated that flux at PEPC exceeded that at Rubisco, providing independent support for substantial back-leakage in low irradiance. Our metabolite analyses are consistent with the idea that back-leakage increases transiently after a sudden decrease in irradiance and decreases transiently after a sudden increase in irradiance, and the metabolite analyses of Sales et al. (2025) provide evidence for similar responses under fluctuating light.

We also asked if our metabolite analyses provide information about how quickly these initial changes of C_{BSC} are reversed. However, later in the ML-LL transition, the 2PG level did not show consistent changes, the RuBP/3PGA ratio rose and then declined and the RuBP/2PG ratio tended to decline. In the LL-ML transition, 2PG levels and the RuBP/3PGA and RuBP/2PG ratios did not show strong or consistent changes. This unclear picture in both transitions may reflect the likelihood that these metabolic traits are influenced by other factors during the gradual adjustment to a change in irradiance (see above).

Another approach to obtain information about C_{BSC} is to compare A_n with RuBP levels, under the assumption that they should correlate unless further factors are influencing Rubisco activity. Such factors would include Rubisco activation (see above) but also C_{BSC}. After a shift to lower irradiance, the 7% recovery of A_n in the recovery phase from about 250 s onwards was accompanied by a large and significant decline in RuBP levels (Figs. 3A, 5B; Supplementary Figs. S3B, S7A). This recovery of A_n and decline of RuBP was accompanied by a significant increase in the level of CCM metabolites like pyruvate and alanine as well as summed CCM metabolites (Figs. 3A, 3C, 5B; Supplementary Figs. S3B, S7C see also Discussion section of the manuscript). These observations are consistent with a decline of C_{BSC} at the trough of A_n at about 250 s, followed by a rise of C_{BSC} as CCM operation is optimized. After a shift to higher irradiance, from about 120 s onwards A_n rose (from about 86 to 140 nmol CO_2 g^{-1} FW s^{-1}) without any further increase in RuBP levels (Figs. 4A, 5C; Supplementary Figs. S4B, S8A), consistent with a gradual increase in C_{BSC}. This explanation is supported by the observation that the increase of A_n was accompanied by a rise in the levels of individual CCM metabolites including PEP, pyruvate, alanine and aspartate and the summed pool size of CCM metabolites (Figs. 4A, 4C, 6C; Supplementary Figs. S4B, S8C). Incidentally, this predicted slow increase in C_{BSC} from about 120 s on contrasts with the decline in back-leakage (and by inference C_{BSC}) after 150 s reported by Wang et al. (2024) after a dark-light transition (see above). This may reflect differences in the speed of activation of the CCM and CBC in these different transitions, or the use of elevated CO_2 in the study of Wang et al. (2024)

6. Sequestration or recycling of C from metabolite pools in the photorespiratory pathway does not make a large contribution in light transients

In C_3 photosynthesis, temporary sequestration of C in the large pools of photorespiratory intermediates and delayed release of CO_2 by glycine decarboxylation (CO_2 bursts and gulps) make a large contribution to the response of A_n after an increase or decrease in irradiance. We inspected the response do metabolite pols in maize to learn if this is also the casein C_4 photosynthesis.

The glycine pool showed marked changes in transitions to lower or to higher irradiance, but there were no consistent changes in serine or glycerate (Figs. 3A, 4A). In the ML-LL transition, glycine decreased by 125 nmol g^{-1} FW between 60 and 600 s (Fig. 3A), equivalent to additional release of CO_2 of about 62 nmol g^{-1}

FW, or about 1.5 s of photosynthesis at the rate sustained in LL. In the LL-ML transition, glycine increased by about 150 nmol g^{-1} FW between 60 and 1200 s (Fig. 4A). equivalent to a reduction in CO_2 release in the photorespiratory pathway of about 75 nmol g^{-1} FW, or only 0.5 s of photosynthesis in ML. Thus, in both cases, changes in the glycine pool can only make a very minor contribution to the delay in reaching the final A_n. The same holds for NADH released during glycine decarboxylation as a potential source of energy.

Incidentally, in C_3 plants some C may exit the photorespiration pathway as glycine or, especially, serine (Fu et al. 2023). If this occurs in C_4 plants, this might further dampen any impact of photorespiratory metabolism during light transitions. However, any impact of photorespiration on transition in C_4 plants is small. Arce-Cubas et al. (2023a) measured response in phylogenetically coupled pairs of C_3 and C_4 species to step-changes from darkness to ML or HL in 21% and 2% O_2, and Arce Cubas et al (2023b) measured responses to fluctuations in 21% and 2% O_2. In all cases, low O_2 had a large impact on the response in C_3 species, in particular by decreasing photorespiration and the associated post-illumination burst of CO_2, but only a small impact in C_4. species.

Additional references

Arp, J., Shrikaar Kambhampati, Kevin L. Chu, Somnath Koley, Lauren M. Jenkins, Todd C. Mockler, and Doug K. Allen (2021) Developmental Effects on Relative Use of PEPCK and NADP-ME Pathways of C4 Photosynthesis in Maize. bioRxiv doi: https://doi.org/10.1101/2021.06.25.449949

Ballicora, M.A., Iglesias, A.A., Preiss, J. (2004) ADP-glucose pyrophosphorylase; a regulatory enzyme for plant starch synthesis. Photosynth Res 79, 1-24. https://doi.org/ 10.1023/B:PRES.0000011916.67519.58

Boag, S., Jenkins, C.L.D. (1986). The involvement of aspartate and glutamate in the decarboxylation of malate by isolated bundle sheath chloroplasts from Zea mays. Plant Physiol. 81: 115–119.

Braütigam, A., Schlüter, U., Lundgren, M.R., et al. (2018) Biochemical mechanisms driving rapid fluxes in C4 photosynthesis. bioRxiv 387431; doi: https://doi.org/10.1101/387431

Chao, Q., Liu, X.Y., Mei, Y.C., Gao, Z.-F., Chen, Y.B., Qian, C.R., Hao, Y.B., Wang, B.C. (2014) Light-regulated phosphorylation of maize phosphoenolpyruvate carboxykinase plays a vital role in its activity. Plant Mol Biol 85, 95–105 https://doi.org/10.1007/s11103-014-0171-3

Cousins, A.B., Badger, M.R., von Caemmerer, S. (2006) Carbonic anhydrase and its influence on carbon isotope discrimination during C_4 photosynthesis. Insights from antisense RNA in *Flaveria bidentis*. Plant Physiol 141, 232-242. https://doi.org/10.1104/pp.106.077776

Cousins, A.B., Badger, M.R., von Caemmerer, S. (2008) C_4 photosynthetic isotope exchange in NAD-ME- and NADP-ME-type grasses. J Exp Bot 59, 1695-1703. https://doi.org/10.1093/jxb/ern001

Evans, J.R., Sharkey, T.D., Berry, J.A., Farquhar, G.D. (1986) Carbon isotope discrimination measured concurrently with gas exchange to investigate CO_2 diffusion in leaves of higher plants. Aust J Plant Physiol 13, 281-292. https://doi.org/10.1071/PP9860281

Fukuyama, H., Masumoto, C., Taniguchi, Y., Baba-Kasai, A., Katoh, Y., Ohkawa, H., Mitsue Miyao,M. (2015) Characterization and expression analyses of two plastidic enolase genes in rice. Biosci Biotechnol Bioche. 79, 402-409. https://doi.org/10.1080/09168451.2014.980219

Furumoto T, Hata S, Izui K (1999) cDNA cloning and characterization of maize phosphoenolpyruvate carboxykinase, a bundle sheath cell-specific enzyme. Plant Mol Biol 41(3):301–311. doi.org/10.1023/A:1006317120460

Hatch, M.D., Kagawa, T. (1976) Photosynthetic activities of isolated bundle sheath cells in relation to differing mechanisms of C4 pathway photosynthesis. Arch Biochem Biophys 175: 39–53. DOI: 10.1016/0003-9861(76)90483-5

Hatch, M.D. (1971) The C_4-pathway of photosynthesis. Evidence for an intermediate pool of carbon dioxide and the identity of the donor C_4-dicarboxylic acid. Biochem J. 125, 425-432. https:// doi: 10.1042/bj1250425

Kromdijk, J., Schepers, H.E., Albanito, F., et al. (2008) Bundle sheath leakiness and light limitation during C_4 leaf and canopy CO_2 uptake. Plant Physiol 148, 2144-2155. https://doi.org/10.1104/pp.108.129890

Kromdijk, J., Griffiths, H., Schepers, H.E. (2010) Can the progressive increase of C_4 bundle sheath leakiness at low PFD be explained by incomplete suppression of photorespiration? Plant Cell Environ 33, 1935-1948. https://doi.org/10.1111/j.1365-3040.2010.02196.x

Ku, S.B., Edwards, G.E. (1975) Photosynthesis in mesophyll protoplasts and bundle sheath cells of various types of CA plants. IV. Enzymes of respiratory metabolism and energy utilising enzymes of photosynthetic pathways. Z. Pflanzenphysiol. 77, 16-32. https://doi.org/10.1016/S0044-328X(75)80122-X

Majeran, W., Friso, G., Ponnala, L., Connolly, B., Huang, M., Reidel, E., Zhang, C., Asakura, Y., Bhuiyan, N.H., Sun, Q., Turgeon, R., and van Wijk, K.J. (2010). Structural and metabolic transitions of C_4 leaf development and differentiation defined by microscopy and quantitative proteomics in maize. Plant Cell 22: 3509–3542.

Meister, M., Agostino, A., Hatch, M.D. (1996) The roles of malate and aspartate in C4 photosynthetic metabolism of Flaveria bidentis (L.). Planta 199: 262–269. https:// doi: 10.1007/BF00196567

Orzechowski S., Socha-Hanc J. and Paszkowski A. 1999. Subcellular distribution of alanine aminotransferase activity in maize (Zea mays L.) leaves. Actae Physiologiae Plantarum 21, 331.334

Pick, T.R., Brautigam, A., Schluter, U., Denton, A.K., Colmsee, C., Scholz, U., Fahnenstich, H., Pieruschka, R., Rascher, U., Sonnewald, U., and Weber, A.P. (2011). Systems analysis of a maize leaf developmental gradient redefines the current C_4 model and provides candidates for regulation. Plant Cell 23, 4208-4220. DOI: 10.1105/tpc.111.090324

Portis, A.R. Jr., Li, C., Wang, D., Salvucci, M.E. (2008) Regulation of Rubisco activase and its interaction with Rubisco. J Exp Bot 59, 1597-1604. https://doi.org/10.1093/jxb/erm240

Portis, A.R. Jr., Parry, M.A. (2007) Discoveries in Rubisco (ribulose 1,5-bisphosphate carboxylase/oxygenase): a historical perspective. Photosynth Res 94, 121-143. https://doi.org/10.1007/s11120-007-9225-6

Sage, R.F. (2014) Stopping the leaks: new insights into C_4 photosynthesis at low light. Plant Cell Environ 37, 1037–1041. https://doi.org/10.1111/pce.12246

Scheibe, R., aStitt, M. (1988) Comparison of NADP-malate dehydrogenase activation, QA reduction and O_2 evolution in spinach leaves. Plant Physiol Biochem 26, 473-481. https://api.semanticscholar.org/CorpusID:82024560

Slack, C.R., Hatch, M.G, Goodchild, D.J.(1969) Distribution of enzymes in mesophyll and parenchyma-sheath chloroplasts of maize leaves in relation to the C4-dicarboxylic acid pathway of photosynthesisBiochem. J. 114, 489-500. https://doi.org/10.1042/bj1140489

Smith, A., Woolhouse, H. W. (1983) Metabolism of phosphoenolpyruvate in the C4 cycle during photosynthesis in the phosphoenolpyruvate-carboxykinase C4 grass Spartina anglica Hubb. Planta 159, 570.578.DOI: 10.1007/BF00409147.

Stitt, M., ap Rees, T.A. (1980) Carbohydrate breakdown by chloroplasts of *Pisum sativum*. Biochim. Biophys. Acta 627, 131-143. https://doi.org/10.1016/0304-4165(80)90315-3

Stryer, L. (1990) Biochemistry. Springer, Heidelberg

Szecowka, M., Heise, R., Tohge, T., et al. (2013) Metabolic fluxes of an illuminated *Arabidopsis thaliana* rosette. Plant Cell 25, 694-714. https://doi.org/10.1105/tpc.112.106989

Tazoe, Y., Noguchi, K., Terashima, I. (2006) Effects of growth light and nitrogen nutrition on the organization of the photosynthetic apparatus in leaves of a C_4 plant, *Amaranthus cruentus*. Plant Cell Environ 29, 691-700. https://doi.org/10.1111/j.1365-3040.2005.01453.x

Ubierna, N., Sun, W., Cousins, A.B. (2011) The efficiency of C_4 photosynthesis under low light conditions: assumptions and calculations with CO_2 isotope discrimination. J Exp Bot 62, 3119-3134. https://doi.org/10.1093/jxb/err073

Wang, L., Czedik-Eysenberg, A., Mertz, R.A., et al. (2014c). Comparative analyses of C_4 and C_3 photosynthesis in developing leaves of maize and rice. Nature Biotechnology 32, 1158-1165. doi.org/10.1038/nbt.3019

Yokochi, Y., Yoshida, K., Hahn, F., Miyagi, A., Wakabayashi, K.I., Kawai-Yamada, M., Weber, A.P.M., Hisabori, T. (2021) Redox regulation of NADP-malate dehydrogenase is vital for land plants under fluctuating light environment. Proc Natl Acad Sci U S A. 118: e2016903118. doi: 10.1073/pnas.2016903118
